# A Decoupled Cycling Architecture of Asymmetric Zinc‐Air Battery Unlocks Stable Catalyst Strategy and pH‐Dynamic Influence

**DOI:** 10.1002/anie.8502321

**Published:** 2026-05-19

**Authors:** Yeshu Tan, Ruinan Wang, Jiawen Huang, Runzhe Chen, Jiaxin Yuan, Siyuan Zhao, Meng Ni

**Affiliations:** ^1^ Department of Building Environment and Energy Engineering Research Institute for Sustainable Urban Development (RISUD) and Research Institute For Smart Energy (RISE) The Hong Kong Polytechnic University Kowloon Hong Kong SAR China; ^2^ College of Materials and Chemical Engineering Minjiang University Fuzhou Fujian China; ^3^ Department of Life Sciences Faculty of Science and Technology Beijing Normal‐Hong Kong Baptist University Zhuhai Guangdong China

**Keywords:** asymmetric zinc‐air battery, decoupled cycling, membrane, stable catalyst strategy, pH‐dynamic influence

## Abstract

An asymmetric zinc–air battery (AZAB) employing membrane‐separated acidic and alkaline electrolytes exhibits high output voltage and superior energy density. However, the bifunctional electrode suffers from severe catalyst degradation during cycling. Furthermore, the influence of pH dynamics on battery performance is challenging to investigate due to catalyst instability. The introduction of a pH gradient provides additional energy to the battery, but its impact on battery efficiency remains unexplored. Round‐trip efficiency (RtE) is an effective indicator of battery efficiency. For fully enclosed batteries, RtE reflects the full capacity. While in half‐open systems, RtE represents only partial capacity and is affected by pH dynamics. Therefore, a decoupled AZAB (DAZAB) featuring separated charge/discharge architectures is developed, which ensures catalyst stability and enables subsequent investigation of pH‐dynamic influences. The contribution of the acid‐base difference in the pH‐decoupled system is described by a universal equation that defines a modified pH‐dynamic RtE for standard comparison, making it suitable for half‐open batteries operating on partial capacity. Moreover, an innovative, low‐cost membrane and a carbon‐based catalyst are fabricated for the battery. The well‐designed DAZAB reveals the influence of pH dynamics on battery performance, offering an innovative pathway for efficient utilization of the acid‐base gradient to achieve high battery efficiency.

## Graphical abstract

A decoupled cycling architecture along with cost‐effective membrane and efficient catalyst is developed for asymmetric zinc‐air battery. The decoupled design ensures stable operation of catalyst and the pH‐dynamic influence on battery performance is explored, which provides paths for efficient utilization of pH‐decoupling electrolytes.

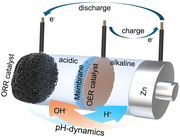

## 1 Introduction

Renewable energy sources are critical for achieving global carbon neutrality and sustainability [1, 2]. The fluctuating nature of wind, solar, and tidal energy requires effective energy storage technologies and conversion strategies to enable their efficient integration into the power grid [3, 4, 5]. Among various energy storage approaches, rechargeable zinc‐air batteries (ZABs) offer outstanding advantages, including a high energy density of 1086 Wh kg^−1^, cost‐effectiveness, environmental friendliness, and intrinsic safety [6, 7, 8].

A conventional ZAB consists of a zinc electrode, an alkaline electrolyte, and an air electrode. The oxygen reduction reaction (ORR) and oxygen evolution reaction (OER) occur at the air electrode during the battery's discharge and charge processes, respectively. The theoretical cell voltage of the alkaline ZAB is 1.66 V, as determined by the reactions on the two electrodes described in Equations (1) and (2) [9].

(1) $$\mathrm{Zn}+2OH^{-}=\mathrm{ZnO}+H_{2}O+2e^{-}\left(E_{0}=-1.256V,\;\;\mathrm{pH}=14\right)$$

(2) $$O_{2}+2H_{2}O+4e^{\text{--}}=4{\text{OH}}^{\text{--}}\left(E_{0}=0.404V,\text{pH}=14\right)$$

The sluggish kinetics of ORR and OER result in a low discharge voltage and a high charge voltage during operation, leading to a comparatively low round‐trip efficiency (RtE) of 60%. Furthermore, adverse conditions, such as formation of insoluble carbonates in alkaline electrolytes, can compromise the air electrode, further degrading battery performance and lifetime [6]. Additionally, the bifunctional catalyst for facilitating both ORR and OER on a single electrode, is prone to clogging and disintegration, resulting in poor stability [10].

In recent years, asymmetric zinc–air batteries (AZABs) with a membrane‐separated dual‐electrolyte configuration have attracted increasing research interest [11, 12]. Owing to the distinct thermodynamic kinetics compared with alkaline media, an acidic environment offers a higher reduction potential for ORR as shown in Equation (3) [12].

(3) $$O_{2}+4H^{+}+4e^{\text{--}}=2H_{2}O\left(E_{0}=1.23V,\text{pH}=0\right)$$

Combined with zinc electrode reaction in an alkaline environment (Equation 1), the AZAB exhibits a theoretical voltage of 2.486 V, which is substantially higher than 1.66 V (alkaline ZAB). Under operating conditions, an even higher RtE of about 70% is attained. For example, Lee's group fabricated a unique membrane tailored for Zn^2+^ transport in an AZAB [13]. With the atomically dispersed Co electrocatalyst as bifunctional catalyst, the AZAB achieved a high discharge potential of 1.96 V at 0.5 mA cm^−2^, along with a high RtE of 76.6% and stable operation exceeding 100 h. In another work, Li applied an LTAP (Li_1+x+y_Ti_2‐x_Al_x_P_3‐y_Si_y_O_12_) membrane to separate an alkaline electrolyte of LiOH and an acidic electrolyte of H_3_PO_4_ [14]. Using an air electrode spray‐coated with a mixture of commercial Pt/C and IrO_2_ catalysts, the AZAB delivered an open‐circuit potential (OCP) of 2.1 V and an RtE of 63.7% at a current density of 0.5 mA cm^−2^. Moreover, Zhang and co‐workers extended the application scope of AZABs by demonstrating a quasi‐solid‐state configuration [15]. The battery exhibited a potential of 1.8 V and an average RtE of 72% at a current density of 3 mA cm^−2^. Despite these advances, AZABs in quasi‐solid‐state form suffered from limited cycle life, which remains a significant barrier to practical implementation.

While asymmetric electrolyte configurations have been demonstrated to elevate the open‐circuit potential (OCP) of Zn–air batteries beyond 2 V and increase RtE to over 70%, the underlying contributions of this acid–base differential to the observed efficiency gains remain largely underexplored. However, the introduction of acid‐base difference imparts additional energy to the battery system, yet its contribution to RtE has rarely been discussed. On the other hand, the influence of pH dynamics on battery performance constitutes a critical factor governing performance. However, the influence of these pH‐dynamic effects on the stability and efficiency of asymmetric Zn‐air batteries has received limited research attention.

Besides the electrolytes, the bipolar membrane commonly employed to separate asymmetric electrolytes presents a significant economic barrier, with cost reaching up to $1300 per square meter [16]. Meanwhile, noble‐metal‐based catalysts remain indispensable for facilitating ORR and OER in an acidic environment. The air electrode is subjected to repeated cycling between OER and ORR conditions, which highly restrict the stability of catalyst [17]. To mitigate this operational instability, several studies have reported strategies involving the chemical or physical decoupling of the OER and ORR processes [18, 19, 20]. Dai's group designed a bifunctional electrode with separated active sites for OER and ORR, which extended the operational stability beyond 300 h in an alkaline environment [18]. This decoupling strategy effectively alleviated oxidative corrosion at the ORR site. Similarly, Ivey's group demonstrated enhanced cycling durability in an alkaline ZAB through the physical separation of the OER and ORR processes [19]. However, efficient strategies for stable catalysts in AZAB remain underexplored, especially in acidic electrolyte.

In summary, the widespread deployment of AZAB is currently impeded by the high cost of both the membrane and the catalyst. Moreover, severe catalyst degradation during prolonged cyclic redox operation significantly limits stability and research into pH‐dynamic influence. More importantly, the contribution of acid‐base difference on battery efficiency needs to be further explored.

Herein, a decoupled AZAB (DAZAB) is designed with a separated charge/discharge architecture during cycling to unlock the stable catalyst strategy for long‐term operation. The DAZAB separates the ORR and OER processes by utilizing asymmetric cell for discharge and alkaline cell for charge independently, which is novelly reported in the AZAB system. Based on the stable catalyst strategy, this work reports the effects of pH dynamics on battery cyclic performance. Furthermore, we present a general equation to quantify the contribution of the acid‐base potential differential to overall battery efficiency, thereby establishing a basis for standardized performance comparison. At the same time, the low‐cost catalyst and membrane are developed for the DAZAB. A membrane is prepared based on polyethersulfone (PES) polymers and sodium alginate (SA) crosslinking channels (PES/SA membrane). The unique channels with abundant functional groups allow efficient transportation of different ions [21, 22]. In addition, cobalt nanoparticles embedded in N and S doped carbon framework (Co/NS‐C) are fabricated for acidic ORR, which become the potential cost‐effective candidate to replace Pt/C. The commercial stainless‐steel (SS) mesh is activated for alkaline OER. Overall, an innovative design of DAZAB with separated charge/discharge architecture is developed. The DAZAB achieves an OCP of 2.16 V and a high energy density of 1322 Wh kg_Zn_^−1^ at a current density of 2 mA cm^−2^. The decoupled design unlocks the stable catalyst strategy, and the study of pH‐dynamic influence indicates the efficient pH gradient range. The DAZAB can achieve the lifespan over 400 h with high discharge voltage under the electrolyte refresh method.

## 2 Results and Discussion

### 2.1 RtE Behind Zinc‐Air Battery

**FIGURE 1:**
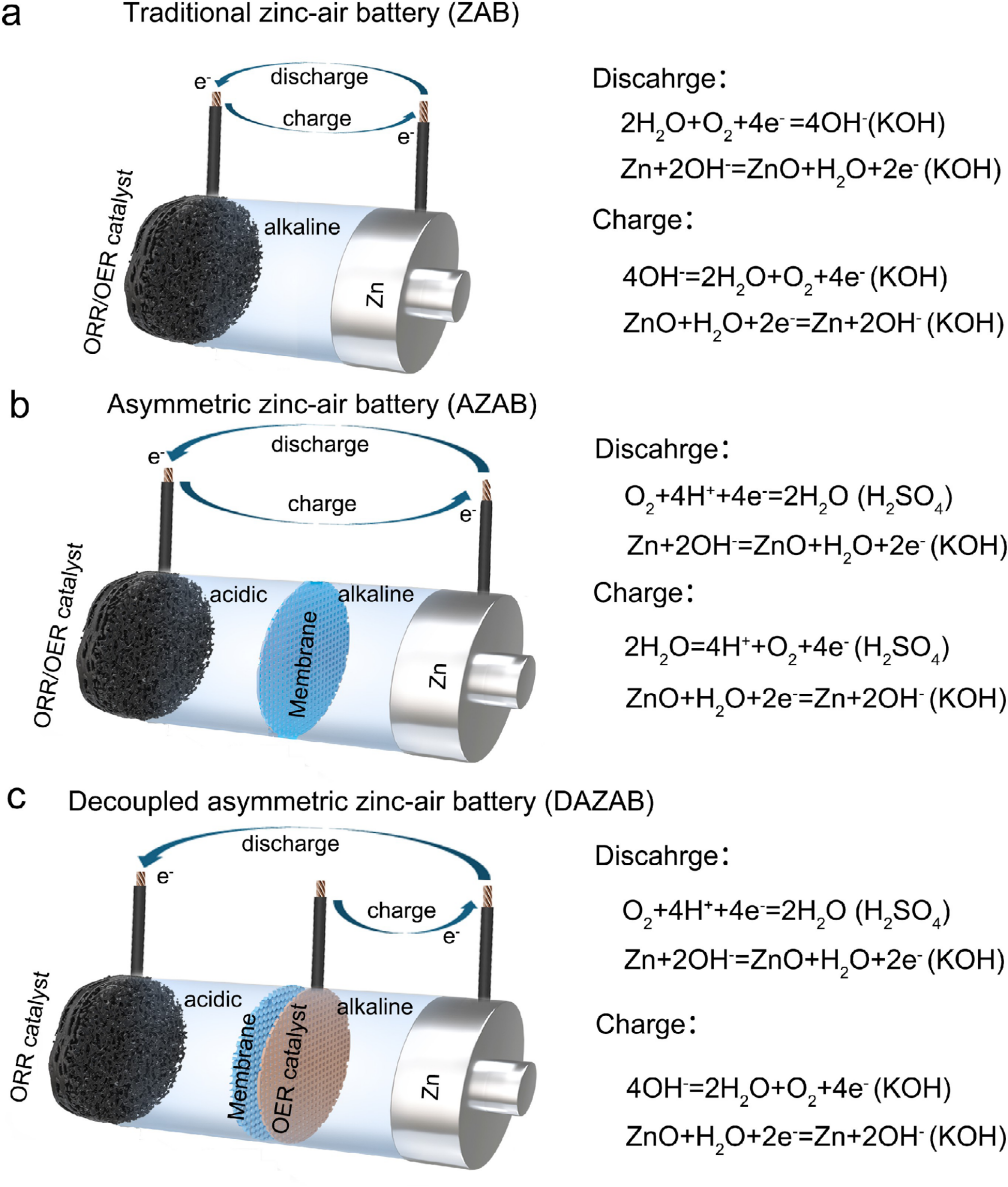
Diagram of different types of zinc‐air batteries.

The zinc‐air battery utilizes pH‐decoupling electrolytes can improve discharge voltage and energy density due to the introduction of acid‐base difference. The diagrams of traditional ZAB and AZAB are listed in Figure 1a,b. Our DAZAB design separates the ORR and OER process in different cells, avoiding the catalyst degradation caused by redox corrosion (Figure 1c). Based on these three kinds of zinc‐air batteries, the RtE is further explored by equations to reveal the contribution from acid‐base difference.

For discharge (ORR) and charge (OER) process, the thermotical potential could be described by Nernst Equation [23, 24]:

(4) $$E=E^{0}\;-\frac{RT}{nF}\;\ln Q=E^{0}\;-\frac{RT}{nF}\ln\frac{a_{Red}}{a_{Ox}}$$

*E* is the electrode potential under given conditions. *E* ^0^ represents the standard electrode potential. *R* is the ideal gas constant, and *T* is the temperature in Kelvins. *n* is the number of electrons transferred, while *F* is the Faraday constant. *Q* is the reaction quotient. *a*_red_ is the activity of the reduced form and *a*_Ox_ is the activity of the oxidized form.

Combined with Equation (3) and standard electrode potential of ORR. The final equation is described as below (where at room temperature, T is 298.15 K, *R* is 8.314 J mol^−1^ K^−1^, *F* is 96485 C mol^−1^, *n* is 4, [H_2_O] is 1, PO_2_ is 1 atm.):

(5) $$\begin{array}{cc} E_{ORR} & =E^{0}-\frac{RT}{nF}\;\ln\frac{{\left[H_{2}O\right]}^{2}}{{\left[H^{+}\right]}^{4}\left(PO_{2}\right)}\\ & =E^{0}\;-\frac{2.303RT}{F}pH=1.23-0.059\times pH \end{array}$$

The relationship between electrode potential of ORR/OER and pH values is illustrated in Figure S1. Meantime, the reaction on zinc electrode can also be described based on Equations (1) and (4) and standard electrode potential of Zn:

(6) $$\;E_{Zn}=-0.43-0.059\times pH$$

Energy‐efficiency analyses for batteries are slightly different in closed and open systems. For a battery in closed system, RtE can be described by a total discharge process (set discharge cut‐off voltage) and a charge process (set charge termination voltage). Summarily, the RtE describes the energy‐efficiency based on total capacity of the battery.

However, all kinds of zinc‐air batteries are half‐open systems, O_2_ is infinite in the air, leading to a stable discharge platform. Same as the stable charge platform with reaction of OER. If the battery is totally discharged, the zinc electrode will be completely consumed, leading to no charge process. For the rechargeable process of zinc‐air batteries, setting consistent discharge, charge currents, and same time duration would be a method to evaluate the cyclic performance. Due to the stable discharge and charge platforms of zinc‐air batteries, the RtE here, indicated by discharge voltage and charge voltage, can describe one cycle energy‐efficiency of battery on partial capacity accurately. In the discussion, we assume that the reaction on Zn electrode is theoretical.

For all kinds of zinc‐air batteries, the discharge voltage and charge voltage are related to the potentials of two electrode, overpotential (related to activity of catalyst), internal resistance, and other consumed potential. Thus, the RtE of different zinc‐air batteries in one cycle is listed.

For ZAB:

(7) $$\begin{array}{cc} \mathrm{RtE} & =\frac{\mathrm{Disc}\mathrm{harge}\;\mathrm{volt}\mathrm{ag}e_{\mathrm{test}}}{\mathrm{Char}\mathrm{ge}\;\mathrm{volt}\mathrm{ag}e_{\mathrm{test}}}=\frac{\left(E_{\mathrm{ORR}}^{\mathrm{base}}-\eta_{\mathrm{ORR}}^{\mathrm{base}}\right)-E_{\mathrm{Zn}}-IR_{d}}{\left(E_{\mathrm{OER}}^{\mathrm{base}}+\eta_{\mathrm{OER}}^{\mathrm{base}}\right)-E_{\mathrm{Zn}}+IR_{c}}\\ & =\frac{1.66-\eta_{\mathrm{ORR}}^{\mathrm{base}}-IR_{d}+pH_{\mathrm{diff}}^{\mathrm{disch}\arg e}\times 0.059}{1.66+\eta_{\mathrm{OER}}^{\mathrm{base}}+IR_{c}+pH_{\mathrm{diff}}^{\mathrm{ch}\arg e}\times 0.059} \end{array}$$

For AZAB:

(8) $$\begin{array}{cc} RtE & =\frac{Discharge\;voltage_{test}}{Charge\;voltage_{test}}\\ & =\frac{\left(E_{ORR}^{acid}-\eta_{ORR}^{acid}\right)-E_{Zn}-IR_{d}-\eta_{mem}}{\left(E_{OER}^{acid}+\eta_{OER}^{acid}\right)-E_{Zn}+IR_{c}+\eta_{mem}}\\ & =\frac{1.66-\eta_{ORR}^{acid}-IR_{d}-\eta_{mem}+pH_{diff}^{disch\arg e}\times 0.059}{1.66+\eta_{OER}^{acid}+IR_{c}+\eta_{mem}+pH_{diff}^{ch\arg e}\times 0.059} \end{array}$$

For DAZAB:

(9) $$\begin{array}{cc} RtE & =\frac{Discharge\;voltage_{test}}{Charge\;voltage_{test}}\\ & =\frac{\left(E_{ORR}^{acid}-\eta_{ORR}^{acid}\right)-E_{Zn}-IR_{d}-\eta_{mem}}{\left(E_{OER}^{base}+\eta_{OER}^{base}\right)-E_{Zn}+IR_{c}}\\ & =\frac{1.66-\eta_{ORR}^{acid}-IR_{d}-\eta_{mem}+pH_{diff}^{disch\arg e}\times 0.059}{1.66+\eta_{OER}^{base}+IR_{c}+pH_{diff}^{ch\arg e}\times 0.059} \end{array}$$

where *η*_ORR_ represents the overpotential of ORR while *η*_OER_ represents the overpotential of OER. *R*_d_ represents the internal resistance during discharge process and *R*_c_ represents the internal resistance during charge process. *I* represents the current and *η*_mem_ represents the extra potential related to membrane. $pH_{diff}^{discharge}$ represents the pH difference value of two electrodes in discharge cell. $pH_{diff}^{charge}$ represents the pH difference value of two electrodes in charge cell. Both values are positive.

During the discharge process, the overpotential must be subtracted to obtain the effective voltage; conversely, the overpotential should be added for actual voltage during the charge part. Furthermore, additional voltages associated with internal resistance and membrane should also be taken into account. For a ZAB, the pH difference is 0, while for an AZAB and a DAZAB, pH difference reflects the contribution from acid‐base difference. During battery operation with crossover of H^+^ and OH^−^ through membrane, the pH difference is dynamic. Therefore, for standard comparison of RtE, the equation should be modified related to pH‐dynamics as follows:

(10) $$\;RtE_{modified}=\frac{Discharge\;voltage_{test}\;-\;pH_{diff}^{discharge}\;\times \;0.059}{Charge\;voltage_{test}\;-\;pH_{diff}^{charge}\;\times \;0.059}$$

The detailed discussion of RtE is described in Supporting Information. The modified equation considers the pH‐dynamic influence and the contribution from acid‐base difference, which is applicable for standard comparisons of different types of zinc‐air batteries that introduce pH gradient.

### 2.2 Synthetic Approach and Characterization of PES/SA Membrane

The PES and PES/SA membrane are synthesized based on solvent‐nonsolvent exchange strategy, which induces phase separation and forms a porous structure [25]. The PES first dissolves in the N‐methyl‐2‐pyrrolidone (NMP) to form the homogeneous solution. Then, the solution is poured into the customed module and immersed into deionized (DI) water sequentially for 24 h. The water molecules diffuse into the solution and greatly decrease the solubility of PES, leading to the separation of PES. The as‐prepared PES membrane is washed by DI water several times and dried under 60°C for 24 h. For synthesis of PES/SA membrane, SA molecules are added in the PES solution with physical grinding by ball milling machine. Detailed approaches are listed in the Experimental Section of the Supporting Information. The mixture solution is poured into the module and immersed in the 0.1 M ZnSO_4_ solution following the same procedure. The PES membrane is formed with combination of SA crosslinking chain of Zn^2+^ simultaneously. SA consists of blocks that alternate between mannuronic and guluronic acid. The creation of SA channels in an aqueous solution is caused by ionic crosslinks between guluronic acid units in various alginate chains and Zn ions [26]. To further verify the morphology of membranes, scanning electron microscopy (SEM) is conducted. Figure s2 illustrates the morphology of PES membrane, indicating the dense and small porous structure. As the preparation of PES/SA membrane is under solvent‐nonsolvent exchange strategy, which induces phase separation and forms a smooth surface on one side (Figure S3a) and porous surface on the other side (Figure S3b). The morphology of large porosity is filled with excess SA. PES can fix the SA channels physically and SA channels can ensure the transportation of different ions. Thus PES/SA membrane is fabricated with low cost and simple strategy to transport ions with unique structure.

**FIGURE 2:**
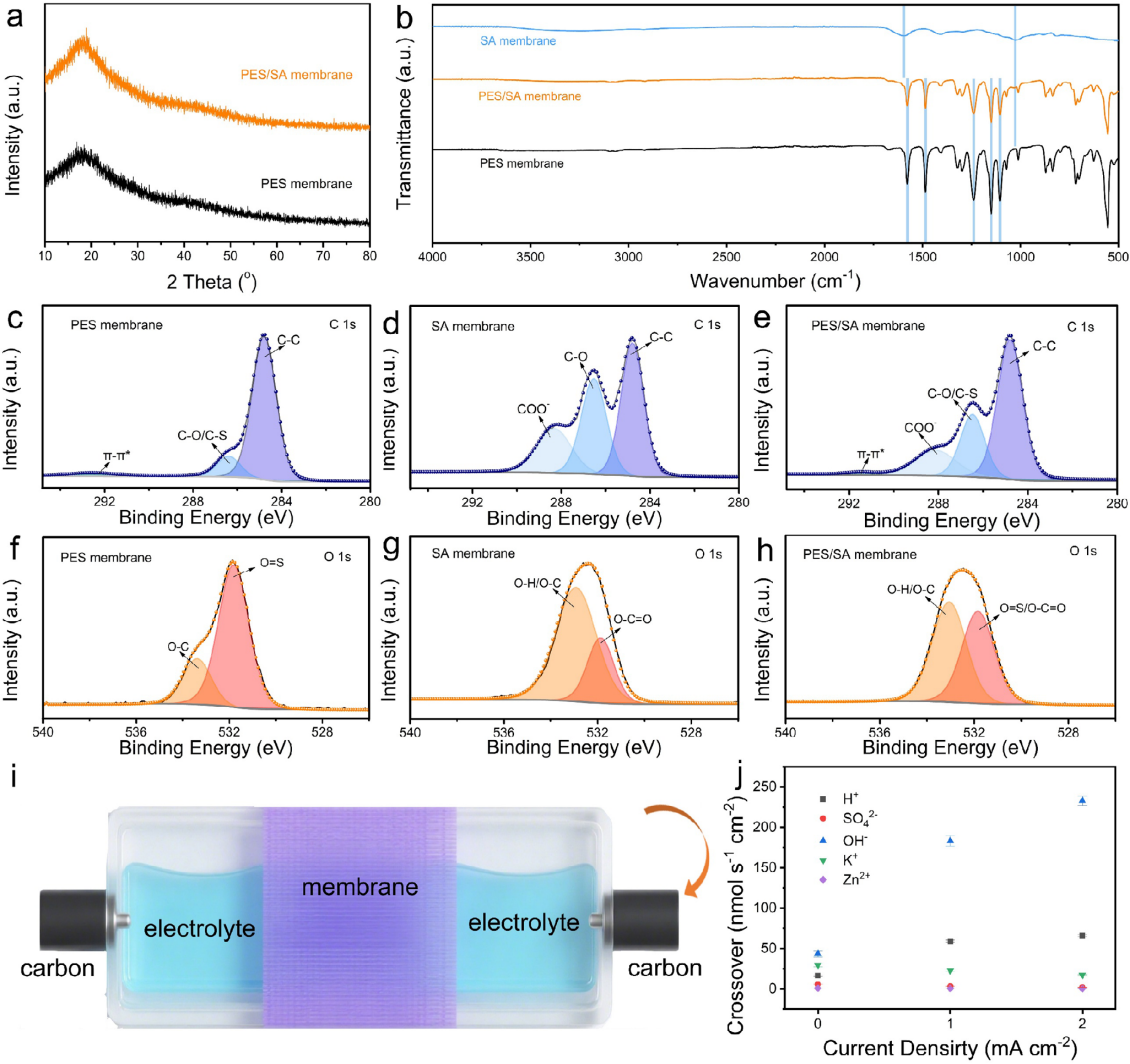
Characteristics of PES and PES/SA membranes. (a) XRD patterns of PES and PES/SA membranes. (b) FTIR spectra of SA, PES and PES/SA membranes. XPS spectra of C 1s for (c) PES, (d) SA and (e) PES/SA membrane. XPS spectra of O 1s for (f) PES, (g) SA and (h) PES/SA membrane. (i) Diagram of test cell. Arrow represents the direction of applied current. (j) Crossover of different ions through PES/SA membrane under different currents.

The as‐prepared membranes are further characterized by x‐ray diffraction (XRD), as shown in Figure 2a. The broad reflection observed from XRD for PES and PES/SA membrane reveals that most regions of these polymer membranes are in an amorphous state [27]. Fourier transform infrared spectroscopy (FTIR) diagrams are presented (Figure 2b). Peaks at 1595 cm^−1^ (SA) are assigned to C = O asymmetric stretching in COOH. PES and PES/SA membranes show identical absorption peaks at 1577 cm^−1^ and 1485 cm^−1^, suggesting the C = C stretching vibration of the benzene ring. Another peak located at 1239 cm^−1^ (PES, PES/SA) represents either linkage between phenyl groups [28]. Meanwhile, peaks at 1149 and 1104 cm^−1^ (PES, PES/SA) indicate sulfone groups in PES structure. The peak near 1025 cm^−1^ (PES/SA and SA) represents the C‐O.

X‐ray photoelectron spectroscopy (XPS) is used to investigate the detailed chemical environment signals. The comprehensive deconvoluted XPS spectra of C 1s for different membranes are illustrated (Figure 2c–e). Peaks located around 292 eV (PES, PES/SA) represent the π‐π^*^ bond [29]. And peak at 286.5 eV indicates the C‐O for SA membrane. While peaks at 286.4 eV (PES) and 286.5 eV (PES/SA) contribute to both C‐O and C‐S. SA and PES/SA membranes have new peaks at 288.3 and 288.2 eV, which are ascribed to COOH [30]. Deconvoluted XPS spectra of O 1s is further illustrated (Figure 2f–h). Peak at 533.4 eV (PES) represents O‐C, while peaks at 532.9 eV (SA) and 533.0 eV (PES/SA) indicate both O‐C and O‐H. At the same time, peaks located at 531.8 eV (PES) and 531.9 eV (SA) contribute to O = S and COO^−^, respectively [31, 32]. For PES/SA membrane, the peak at 531.8 eV represents both group signals. The XPS of S 2p is also revealed (Figure S4). For basic PES structure, peaks located both at 169.0 eV (PES, PES/SA) are attributed to O = S = O. Additionally, peaks at 167.8 eV (PES, PES/SA) represent C‐S = O [33]. Due to the crosslinking mechanism of SA. The Zn 2p XPS spectra is also presented (Figure S5). The observed peaks reveal the integration of Zn^2+^ for PES/SA and SA membranes [34].

To verify the crossover value of different ions under battery condition, 6 M KOH with 0.2 M Zn(OAc)_2_ or 3 M H_2_SO_4_ solution is filled in the asymmetric cell, as shown in Figure 2i. The calculation equation of crossover is discussed in Supporting Information. The arrow represents the direction of current. The applied currents are 0, 1 and 2 mA cm^−2^ for continuous 1 h to simulate the internal electric field during battery working. The concentration of H^+^ and OH^−^ are tested by pH meter, while Zn^2+^ and K^+^ are evaluated by inductively coupled plasma (ICP). SO_4_^2−^ ions are tested by ion chromatography (IC). To calculate the crossover of H^+^ and SO_4_^2−^, 8 mL of 3 M H_2_SO_4_ is added in the right cell and 8 mL of DI water is added in the left cell. Five PES/SA membranes are used to evaluate the average ions crossover. pH values and concentrations of SO_4_^2−^ ions in DI water after transportation for 1 h under different currents are listed in Table S4. The H^+^ crossover has average values of 16.42 (no current), 59.01 (1 mA cm^−2^) and 65.84 (2 mA cm^−2^) nmol s^−1^ cm^−2^. The SO_4_^2−^ ions have average crossover values of 5.61 (no current), 3.39 (1 mA cm^−2^), and 2.13 (2 mA cm^−2^) nmol s^−1^ cm^−2^. The crossover values show the fast transfer of H^+^ in acid cell.

The crossovers of OH^−^, K^+^ and Zn^2+^ are also calculated. Eight milliliters of 6 M KOH with 0.2 M Zn(OAc)_2_ are added in the left cell and 8 mL of DI water is added in the right cell. Table S5 lists the pH value and concentrations of K^+^ and Zn^2+^ ions in DI water after 1 h transportation under different currents. The OH^−^ crossover has average values of 43.66 (no current), 182.96 (1 mA cm^−2^), and 232.78 (2 mA cm^−2^) nmol s^−1^ cm^−2^. The values are much larger than that of H^+^, which is due to the OH^−^ environment would make carboxyl groups in channels become negatively charged carboxylate ions (‐COO^−^). They began to repel each other and make polymer chains to stretch out and relax, leading more space for transportation of ions [35]. The Zn^2+^ ions have average crossover values of 0.94 (no current), 0.65 (1 mA cm^−2^), and 0.36 (2 mA cm^−2^) nmol s^−1^ cm^−2^. While K^+^ ions have average crossover values of 29.44 (no current), 22.76 (1 mA cm^−2^), and 17.17 (2 mA cm^−2^) nmol s^−1^ cm^−2^. Figure 2j illustrates the average crossover value of different ions under various current densities. At static state (no current), the crossover of OH^−^ has the highest value, followed by K^+^. This is due to the OH^−^ expands the transportation channels. After applying currents, OH^−^ still has the highest value of crossover and followed by H^+^. This is due to the internal electric field accelerates the transportation of OH^−^ and H^+^. While K^+^, Zn^2+^ and SO_4_^2−^ have decreased transmitting rate, which is hindered by the electric field. Regarding to the water permeation, the transport water is collected after 24 h with amount of 0.843 mL. The water permeation value is 479 nmol s^−1^ cm^−2^ at static state.

The importance of SA crosslinking channels rather than PES porous structure is also confirmed by using a PES membrane in the test cell. The current verse voltage curve in both 0.5 M H_2_SO_4_ electrolytes is illustrated (Figure S6). A new membrane based on PES/SA mixture without Zn^2+^ crosslinking during preparation process is also tested. The curves illustrate that both membranes are almost in open circuit condition, indicating no transportation of ions. The mechanical stability of PES/SA membrane is illustrated by bending test. The membrane is bent with 180° each time until the membrane is cracked, as shown in Figure S7a. The number of bends is accounted to evaluate the mechanical stability. Figure S7b illustrates the average amount of bends at 56.7 with a standard deviation of 4.76 among ten membranes.

### 2.3 N‐ and S‐Doped Carbon With Embedded Co Nanocrystals

**FIGURE 3:**
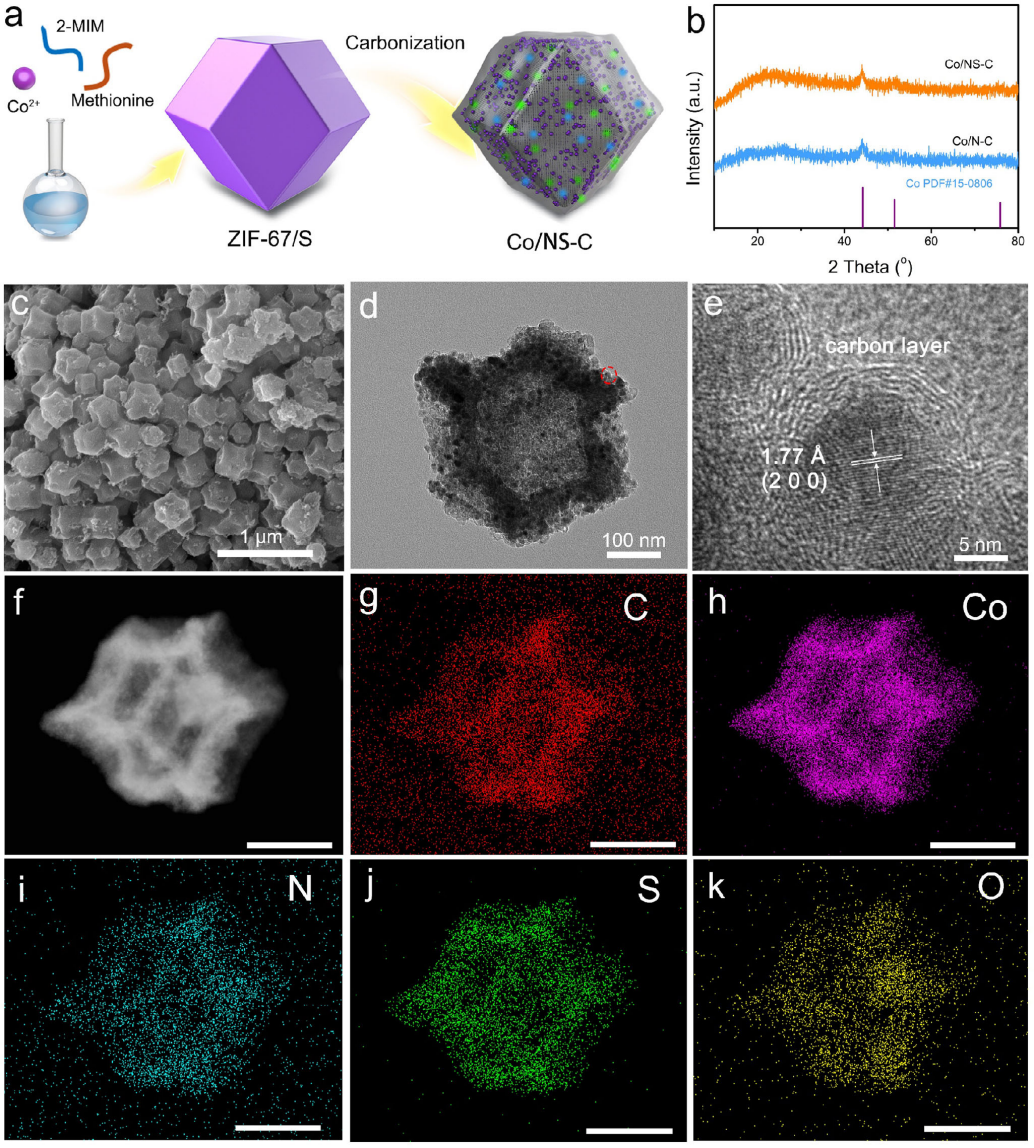
Synthetic approaches and morphologic characteristics of catalysts. (a) Synthetic diagram of Co/NS‐C. (b) XRD patterns of Co/N‐C and Co/NS‐C. (c) SEM image, (d) TEM image, and (e) HRTEM image of Co/NS‐C. (f) HAADF image of Co/NS‐C. Corresponding EDX mapping image of element of (g) C, (h) Co, (i) N, (j) S, and (k) O. Scale bar is 200 nm.

To fabricate N and S doped carbon with embedded cobalt nanocrystals (Co/NS‐C), ZIF‐67/S is first prepared. Figure 3a illustrates the synthetic approach of Co/NS‐C. Co^2+^, 2‐methylimidazole (2‐MIM), and methionine are dissolved in the mixture solution of water and methanol. The detailed process is described in the Experimental Section of the Supporting Information. After stirring at room temperature for 2 h, the growing ZIF‐67/S nanoparticles are collected by centrifugation and washed by methanol several times. After drying under 60°C, the purple powder is prepared for carbonization in the joule heating machine with filled N_2_ for 10 s at 800°C. For normal cobalt nanocrystals embedded in N carbon (Co/N‐C), ZIF‐67 is fabricated under the same procedure except adding methionine (Figure S8). XRD and SEM are utilized to explore the materials. Both as‐prepared ZIF‐67 and ZIF‐67/S show the standard XRD patterns of ZIF‐67 (Figure S9) [36]. The corresponding SEM images (Figure S10) illustrate a rhombic dodecahedron morphology, implying that the addition of methionine does not change the shape growth of nanoparticles. After carbonization, these two catalysts are under acid treatment to remove the surface Co nanoparticles. Both Co/N‐C and Co/NS‐C are verified by XRD patterns (Figure 3b). Obvious peak located at 44.1° represents the (111) phase of metallic Co. Due to the quick decomposition of organic ligands and stress difference between the inner and outer layers during pyrolysis, the SEM images (Figures S11 and 3c) display a contracted and roughened shape of carbonized nanoparticles [37]. Co/N‐C and Co/NS‐C are further explored by transmission electron microscope (TEM). TEM image (Figure 3d) clearly shows that small nanoparticles are embedded in the carbon framework of Co/NS‐C. The chosen area with red cycle (Figure 3e) is enlarged as high resolution‐TEM (HR‐TEM). The lattice spaces of 1.77 Å is contributed to (200) phase of metallic Co. Meantime, carbon layers are clearly visible surrounding the Co nanoparticles, confirming the embedded structure. The high angle annular dark field (HAADF) image is illustrated (Figure 3f). The energy dispersive x‐ray spectroscopy (EDX) mapping images are shown with corresponding elements of C, Co, N, S, and O, respectively (Figure 3g–k). The uniform distribution of each element indicates the homogeneous fabrication of Co/NS‐C. Figure S12 shows the TEM image and HRTEM image of Co/N‐C. The corresponding lattice space of the small nanoparticles is 2.05 Å, which is assumed to (111) phase of metallic Co. The HAADF image is illustrated along with the corresponding EDX mapping image (Figure S13). The element C, Co, N, and O are distributed uniformly on the surface for Co/N‐C.

**FIGURE 4:**
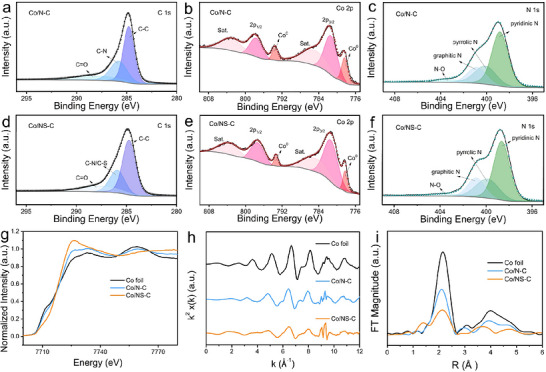
Chemical environmental characteristics of catalysts. XPS spectra of (a) C 1s, (b) Co 2p, and (c) N 1s for Co/N‐C. XPS spectra of (d) C 1s, (e) Co 2p, and (f) N 1s for Co/NS‐C. (g) K‐edge XANES spectrums of Co foil, Co/N‐C, and Co/NS‐C. (h) *k* ^2^‐weighted extracted EXAFS signals. (i) Fourier‐transformed EXAFS spectrums.

XPS is conducted to characterize the local environment and study the influence of S element in as‐prepared materials. The deconvoluted XPS spectra of C 1s for Co/N‐C and Co/NS‐C are revealed (Figure 4a,d). Peak at 285.7 eV (Co/N‐C) is associated with C‐N, while peak at 285.9 eV (Co/NS‐C) contributes C‐N and C‐S [38]. The positive shift indicates the electron decrease around C. In addition, the deconvoluted XPS spectra of Co 2p for Co/N‐C and Co/NS‐C are illustrated (Figure 4b,e). Peaks located at 778.4 eV (Co/N‐C) and 778.2 eV (Co/NS‐C) are ascribed to Co^0^ 2p_3/2_. The interaction between Co^0^ and N in Co/N‐C increases the binding energy of Co^0^ 2p_3/2_ because the electron adsorption ability of N [39]. After doping the other element of S, the binding energy of Co^0^ 2p_3/2_ has a negative shift of 0.2 eV, confirming the electrons enhancement around Co. This is because S doping can weaken the interaction between N and Co by interacting with N [37]. In addition, peaks at 781.4 eV (Co/N‐C) and 781.6 eV (Co/NS‐C) are ascribed to Co‐O 2p_3/2_. The positive shift of 0.2 eV indicates that the Co moves to higher oxidation state after S doping. Meanwhile, the deconvoluted XPS spectra of N 1s for Co/N‐C and Co/NS‐C are presented (Figure 4c,f), respectively. Peaks located at 398.8 eV (Co/N‐C) and 398.6 eV (Co/NS‐C) are ascribed to pyridinic N. While peaks at 400.1 eV (Co/N‐C) and 399.9 eV (Co/NS‐C) contribute to pyrrolic N. Moreover, peaks located at 400.9 eV (Co/N‐C) and 400.8 eV (Co/NS‐C) contribute to graphitic N [38]. After S doping, all the relevant N binding energies have a negative shift, which implies the electron enhancement around N. S site becomes the extra electron donor for N by interacting with N directly, leading the electrons change around both N and C sites. On the other hand, the XPS spectra of O 1s spectra is illustrated (Figure S14). Peaks at 533.2 eV (Co/N‐C) and 533.1 eV (Co/NS‐C) correspond to O‐C. Additionally, the XPS spectra of S 2p for Co/NS‐C (Figure S15) indicate that peaks located at 161.8 and 163.1 eV are ascribed to S 2p_3/2_ and S 2p_1/2_ [40].

X‐ray absorption fine structure (XAFS) is utilized to further explore the chemical state and coordination structure of Co/N‐C and Co/NS‐C. The Co K‐edge x‐ray absorption near edge structure (XANES) spectrums of Co foil, Co/N‐C, and Co/NS‐C are presented (Figure 4g). The absorption edge of Co/N‐C is located close to Co foil, and the absorption edge of Co/NS‐C is little beyond Co foil, indicating the average valence state of Co in Co/N‐C is close to Co (0), while Co in Co/NS‐C has more oxidation state, which is consistent with XPS results [41]. *k* ^2^‐weighted extracted EXAFS signals (Figure 4h) and the Fourier‐transformed extended x‐ray absorption fine structure (EXAFS) spectrums are illustrated (Figure 4i). A prominent peak at around 2.14 Å is associated with the Co‐Co path of Co foil, which is also observed in Co/N‐C and Co/NS‐C, presenting the existence of metallic Co nanoparticles [41]. All the characterization results reveal that Co mainly exists as metallic nanoparticles, which are embedded in carbon materials. Moreover, the S element is interacted obviously with N. The structure is formed due to joule‐heating strategy for ultra‐fast heating and cooling, leading the unusual interaction. The interaction between S and N alters the interaction of N site with both C and Co, synergistically affecting the local electron environment.

### 2.4 ORR Performance and DFT Calculations

**FIGURE 5:**
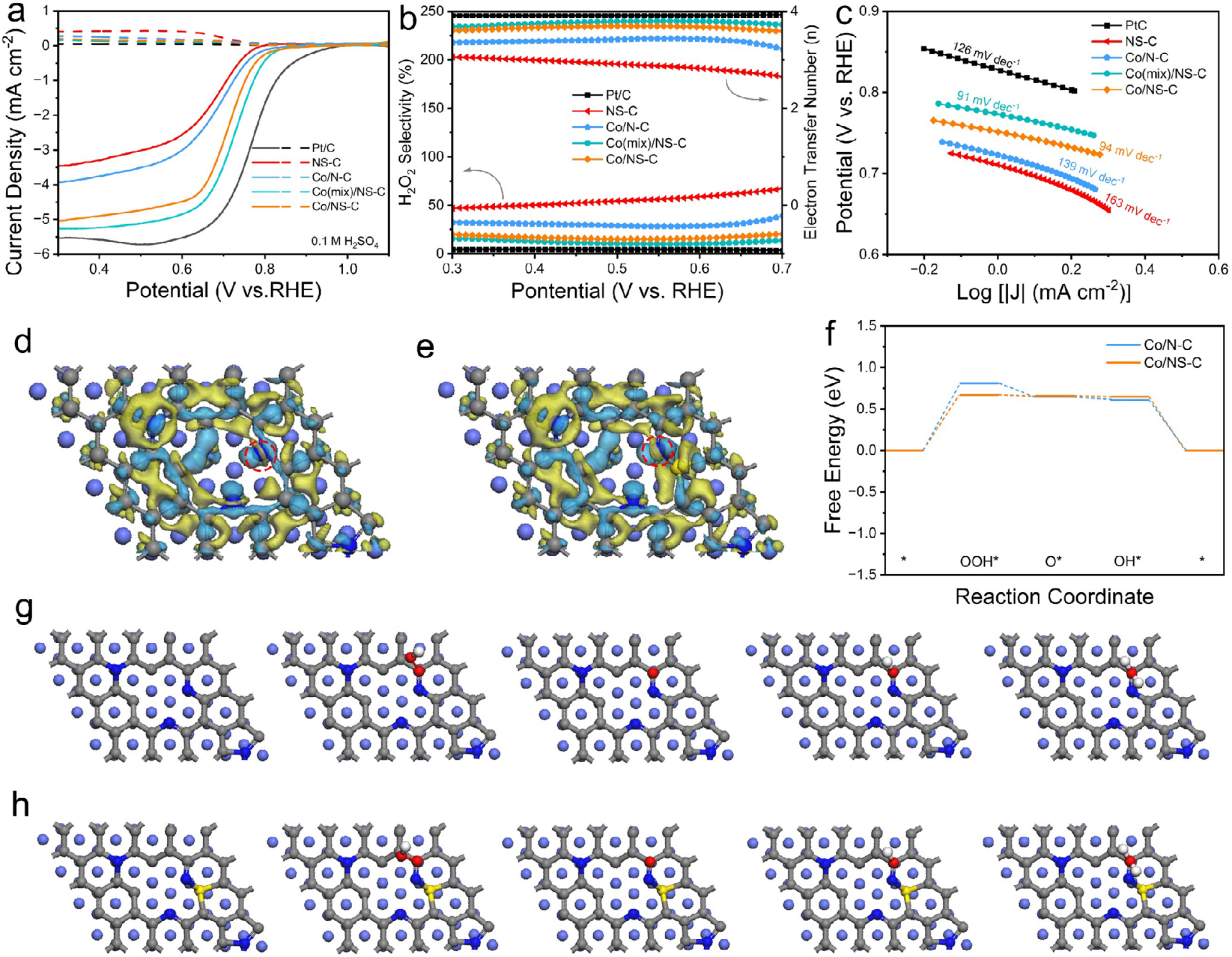
ORR performance and DFT calculations. (a) ORR polarization curves of disk (solid line) and ring (dash line) current density of NS‐C, Pt/C, Co/N‐C, Co(mix)/NS‐C, and Co/NS‐C in O_2_ saturated 0.1 M H_2_SO_4_. (b) Calculated selectivity of H_2_O_2_ and electron transfer number. (c) Tafel plots of corresponding catalysts. Charge density difference of (d) Co/N‐C and (e) Co/NS‐C. Yellow and cyan represent area of charge depletion and accumulation. (f) Free energy diagram of ORR process for Co/N‐C and Co/NS‐C (*U* = 1.23 V). Schematic diagrams of optimized intermediate models for ORR process of (g) Co/N‐C and (h) Co/NS‐C. The indigo, blue, gray, yellow, red, and white balls represent cobalt, nitrogen, carbon, sulfur, oxygen, and hydrogen, respectively.

The as‐prepared materials are used for ORR in acidic medium. To make a thoroughly comparison of catalysts. Pt/C, N and S doped carbon (NS‐C), cobalt nanoparticles embedded in N doped carbon (Co/N‐C), cobalt nanoparticles disperse on surface and embedded in framework of N, S doped carbon (Co(mix)/NS‐C) and cobalt nanoparticles embedded in N, S doped carbon (Co/NS‐C) are fabricated for ORR in acidic medium. Co(mix)/NS‐C is directly used without acid treatment. SEM images illustrate the catalyst of NS‐C and Co(mix)/NS‐C (Figure S16). The catalyst powder is casted on rotating ring disk electrode (RRDE). A graphite electrode and an Ag/AgCl electrode are utilized as counter and reference electrode. The ORR activities of as‐prepared catalysts are evaluated by linear sweep voltammetry (LSV) curves in O_2_ saturated 0.1 M H_2_SO_4_ under 1600 rpm of RRDE (Figure 5a). The Pt/C catalyst presents remarkable ORR activity with a half‐wave potential (*E*_1/2_) of 0.78 V. The NS‐C catalyst shows obvious ring current, indicating the reaction of 2 electrons. It illustrates poor 4 electron activity with a *E*_1/2_ of 0.67 V. The Co/N‐C catalyst shows better performance than NS‐C with a *E*_1/2_ of 0.68 V. With extra S doping, the Co/NS‐C illustrates the improved ORR performance with a *E*_1/2_ of 0.71 V. The Co nanocrystals on surface in Co(mix)/NS‐C as metallic catalyst further improves the catalytic performance with a *E*_1/2_ of 0.73 V. High catalytic selectivity is a significant describer for an ORR catalyst in addition to strong catalytic activity. The H_2_O_2_ selectivity and electron transfer number are calculated in Figure 5b. The calculation equations are discussed in Supporting Information. The NS‐C materials show highest H_2_O_2_ selectivity, reaching 50%. With the introduction of Co nanoparticles, the H_2_O_2_ selectivity decreases with an increasing electron transfer number. Co/N‐C still achieves a H_2_O_2_ selectivity of 30% and an electron transfer number in the range of 3.2–3.4. With extra S doping, the H_2_O_2_ selectivity further decreases to 16% and 10% for Co/NS‐C and Co(mix)/NS‐C, respectively. The electron transfer numbers both increase to the range of 3.6–3.8, implying a preference on 4 electron reaction of the ORR process. Pt/C shows the lowest H_2_O_2_ selectivity around 5% and the highest electron transfer number around 3.9. Tafel slopes of 126 mV dec^−1^ (Pt/C), 163 mV dec^−1^ (NS‐C), 139 mV dec^−1^ (Co/NS‐C), 91 mV dec^−1^ (Co(mix)/NS‐C), and 94 mV dec^−1^ (Co/NS‐C) are illustrated in Figure 5c.

The Co(mix)/NS‐C shows remarkable ORR performance, and the stability is further evaluated by long‐term cycling. The LSV curves before and after 3000 cycles in acidic medium are illustrated (Figure S17). The Co(mix)/NS‐C shows a large decrease in catalytic performance after 3000 cycles, which is due to the heavy corrosion of exposed Co on the surface in acidic medium. The Co leaching happens severely during long‐term acidic ORR. The leaching amount of Co is evaluated by ICP with a value of 0.492 ppm. Thus, the Co(mix)/NS‐C is not a suitable choice for long‐term rechargeable battery. Then, the Co/NS‐C catalyst is further evaluated by long‐term cycling in an acidic medium, as shown in (Figure S17b). The Co/NS‐C catalysts show fabulous stability in acidic medium. The leaching amount of Co is also evaluated by ICP with a value of 0.063 ppm. The TEM image after 3000 cycles test is presented to verify the morphology change (Figure S18). The Co/NS‐C leaves small Co nanoparticles in the carbon framework, ensuring a stable structure for ORR. Additionally, ORR reaction of Co/NS‐C under different spin speeds is evaluated (Figure S19). With an increase in spin speed of RRDE from 400 to 2500 rpm, the current density of the platform changes from −2.6 to −5.6 mA cm^−2^.

The effect of S doping on the Co/N‐C structure is investigated theoretically using density functional theory (DFT). The structures of Co/N‐C and Co/NS‐C are optimized based on analytic information from XPS, TEM, and XAFS. Co nanoparticles are embedded into the carbon framework and the dominant C‐N interaction is pyridinic N. S is interacted with N due to the unique fabricating strategy (Figure S20). The diagram showing the charge density difference provides a visual representation of the electron transport following S doping. From the top view of model for Co/N‐C, the transfer of electrons between C and N (red circle) is clearly observed (Figure 5d). While the rearrangement of electrons shows different trends in Co/NS‐C structure that a charge accumulation appears obviously around the N (red circle) bonded to S and C, which is due to the strong electron donating from S (Figure 5e). For Co/N‐C, two C sites near N show the symmetric electron environment, while S doping breaks the balance and builds an asymmetric electron environment for Co/NS‐C. Due to the embedded structure, the intermediates are absorbed on priority on the carbon layer, making the C site the main active site [42, 43]. The C site next to N becomes the effective site in both Co/N‐C and Co/NS‐C for optimized models. The Gibbs free energy of intermediates for adsorption on Co/N‐C and Co/NS‐Cis calculated in order to obtain a deep understanding of the critical role that S doped Co/N‐C structure plays in the ORR process (Figure 5f). ORR takes place on the catalyst surface by producing OOH* from adsorbed O_2_ and then further reduction to O* and OH* (Figure 5g,h). At the equilibrium potential of 1.23 V versus RHE, the rate‐determining step (RDS) is defined by the minimized energy barrier [44]. For Co/N‐C and Co/NS‐C, the hydrogenation of O_2_ to OOH* is the RDS, which requires a barrier of 0.81 and 0.67 eV, respectively. The lower energy barrier is due to the S doping, breaking the symmetric accumulation of electrons around N, which induces less accumulation of electrons around the C site next to N, which is consistent with XPS results. Thus, the C site shows high activity for ORR intermediates.

Meanwhile, to verify the importance of Co nanoparticles in the carbon framework, pure carbon layer N‐C and NS‐C structures are also calculated, and the free energy diagrams are presented (Figure S21). The RDS requires 0.93 and 1.05 eV for N‐C and NS‐C, respectively. The Co also interacts with the carbon layer to tune the adsorption energy of intermediates for ORR. The reaction paths of intermediates on N‐C and NS‐C models are illustrated in Figure S22. In summary, the S doping in Co/NS‐C interacts with N, breaking the symmetric electron distribution around the N site and leading electron depletion of the C site next to N with lower energy barriers of RDS, which accelerates the efficient ORR process. Meanwhile, the embedded Co nanoparticles also interact with the carbon layer to tune the electron distributions.

### 2.5 Performance of DAZAB

The performances between DAZAB system and AZAB system with commercial Pt/C catalyst for ORR are compared. The AZAB system uses a mixture of commercial Pt/C and RuO_2_ as a bifunctional air electrode for ORR and OER. The DAZAB system uses Pt/C as air electrode for ORR and activated SS mesh as OER electrode. Both batteries utilize a PES/SA membrane. For AZAB system, the long‐term battery performance is shown in Figure S23a. The electrolytes used in asymmetric battery are 6 M KOH with 0.2 M Zn(OAc)_2_ and 3 M H_2_SO_4_. The discharge voltage decreases sharply at around 10 h along with an increment of charge voltage. This is due to catalyst degradation caused by oxidation corrosion. The electrolyte in the inserted figure indicates the oxidation corrosion of carbon materials, which is the main problem of the bifunctional electrode. Figure S23b illustrates the enlarged cycles around 40 h; the discharge voltage decreases to 1.45 V, and the charge voltage increases to around 2.8 V. In contrast, DAZAB with Pt/C as catalyst shows remarkable cyclic performance for 80 h in Figure S23c. The inserted figure shows the clear electrolyte (ORR cell) after 80 h, indicating the advantage of the decoupled configuration to separate the OER and ORR in different cells. It avoids the redox corrosion, especially for carbon materials. The enlarged battery performance of DAZAB in Figure S23d illustrates that the discharge platform is around 1.99 V, and the charge platform is around 2.01 V.

By employing the separated charge/discharge architecture, the decoupled battery can ensure the stability of catalysts. The as‐prepared catalysts are used on an air electrode for ORR during discharge process, and an SS mesh is used for OER during charge process. The SS mesh has fabulous OER activity after activation by cyclic voltammetry (CV) process (Figure S24) [45]. After activation, the SS mesh shows quite different activity for OER (Figure S25a). LSV curves of SS mesh before and after activation present a difference overpotential of 53 mV at a current density of 10 mA cm^−2^. The activated SS mesh is also performed in 6 M KOH and 6 M KOH with 0.2 M Zn(OAc)_2_, respectively. The LSV curves are illustrated in Figure S25b. The high concentration of KOH accelerates the OER kinetics. The overpotential of activated SS has a difference of 4 mV at a current density of 50 mA cm^−2^ with addition of zinc salts.

**FIGURE 6:**
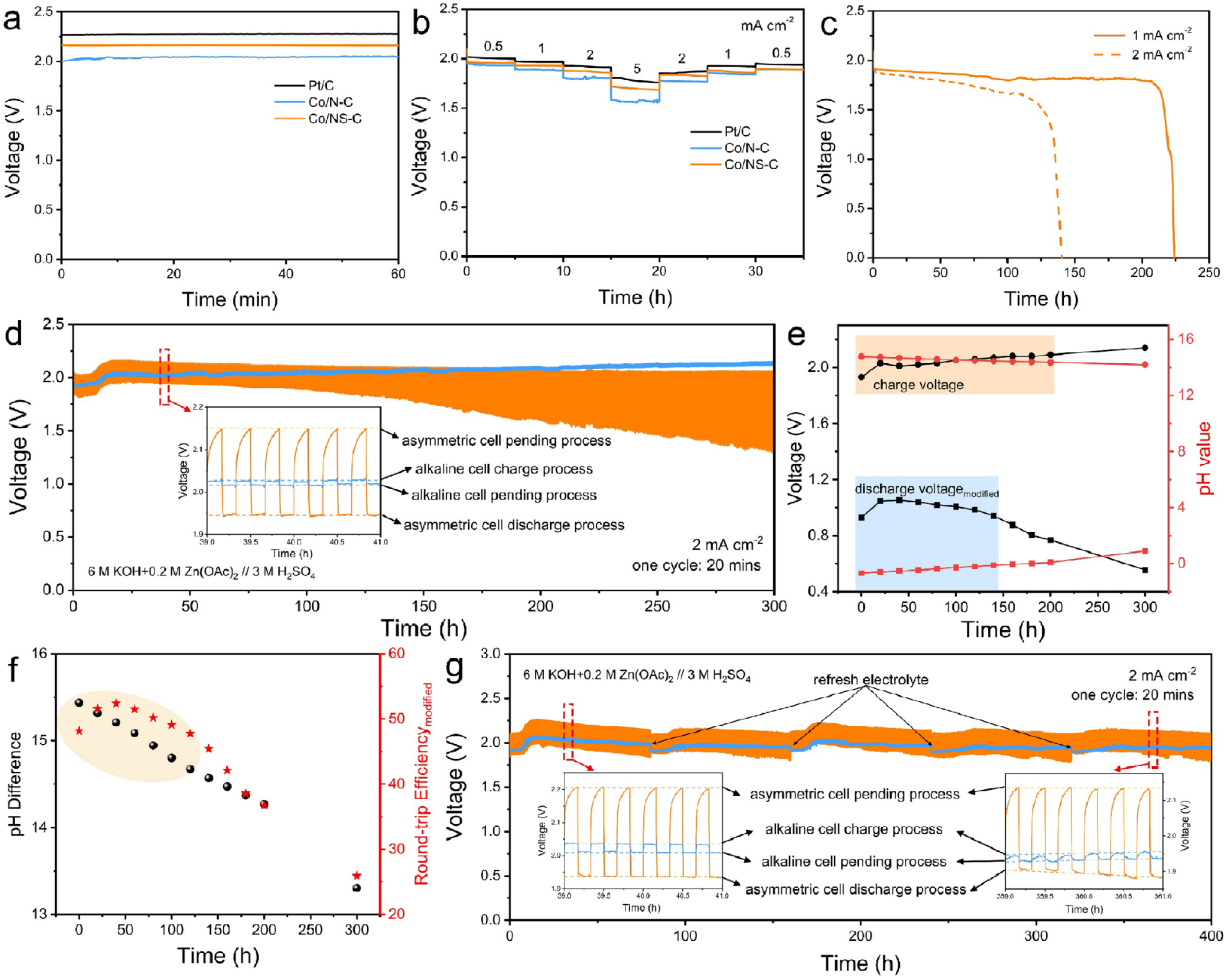
Performance of DAZAB. (a) Open circuit potential, (b) step discharge curves of DAZAB based on different air electrodes. (c) Continuous discharge curves of DAZAB based on Co/NS‐C. (d) Cyclic performance of DAZAB with Co/NS‐C as air electrode for 300 h. (e) Relationship between pH value and charge and discharge voltages (modified). (f) Relationship between pH difference and modified round‐trip efficiency. (g) Cyclic performance of DAZAB based on Co/NS‐C with refresh of electrolyte.

All DAZABs use a PES/SA membrane for tests. The OCP curves based on DAZAB with Pt/C, Co/N‐C and Co/NS‐C as air electrode are shown in Figure 6a. The electrolytes are 6 M KOH with 0.2 M Zn(OAc)_2_ and 3 M H_2_SO_4_. Due to the sluggish ORR reaction of Co/N‐C, it only delivers an OCP of 2.03 V. With state‐of‐the‐art performance of Pt/C in ORR, DAZAB delivers an OCP of 2.27 V. DAZAB based on Co/NS‐C has a comparable OCP of 2.16 V. OCP here is the foundational indicator of a battery's thermodynamic potential. Its importance lies in the fact that a higher OCP directly correlates with high energy capabilities. The values in DAZAB reflect the catalytic performance of different materials.

The Nyquist plot curves (Figure S26) show that DAZAB based on Pt/C, Co/N‐C, and Co/NS‐C has an obvious difference of internal and charge transfer resistances. The difference indicates that Co/NS‐C has a better path for charge transfer than Co/N‐C on the surface. The step discharge curves of DAZAB based on different catalysts are illustrated (Figure 6b). DAZAB based on Pt/C has the highest discharge platform under current densities of 0.5, 1, 2, and 5 mA cm^−2^. The recovery performance is also remarkable. The DAZAB based on Co/NS‐C shows comparable discharge performance, which is better than Co/N‐C. The DAZAB with Co/N‐C as air electrode has the lowest step discharge platform. Additionally, the continuous discharge under certain current densities is performed (Figure 6c). DAZAB based on Co/NS‐C shows a long‐term discharge voltage from 1.93 to 1.79 V under 1 mA cm^−2^, reaching near 210 h. When increasing the current density to 2 mA cm^−2^, the discharge voltage experiences a decrease from 1.91 to 1.68 V, and the battery can operate for 125 h. The capacity and energy density are calculated. The detailed calculation steps are described in Supporting Information. The calculated value of capacity is 778.7 mAh g_Zn_^−1^ for 2 mA. While the capacity calculated from 1 mA is 627.17 mAh g_Zn_^−1^. The difference is caused by the side reaction and long‐term influence caused by pH‐dynamics. For 2 mA discharge curve, it has a high energy density of 1322 Wh kg_Zn_^−1^. While for the 1 mA discharge curve, it has an energy density of 1132 Wh kg_Zn_^−1^.

To verify the rechargeable performance of DAZAB, the asymmetric cell is used for the discharge process, and the alkaline cell is carrying out the charge process. The long‐term cyclic performance of DAZAB based on Co/NS‐C and SS mesh for 300 h is illustrated in Figure 6d. The inserted figure presents the cycles around 40 h. The vibration of first 10 h was attributed to the establishment of electrolyte equilibrium. The ORR and OER reactions are separated based on the decoupled design, which ensures the stable operation of catalyst during charge and discharge. However, the discharge voltage has a huge difference after long‐term operation and decreases to 1.34 V (300 h). The charge voltage slightly increases and reaches around 2.14 V (300 h). Thus, we consider the pH‐dynamic influence to be the main factor affecting the battery performance. The ORR and OER reaction kinetics under various pHs differ considerably. The crossover of H^+^ and OH^−^ would cause the pH‐dynamic changes during battery operation, leading to the influence of battery performance. The pH values are recorded through the holes on cell at 0, 20, 40, 60, 80, 100, 120, 140, 160, 180, 200, and 300 h to verify the efficient pH range for battery performance, as listed in Table S6. The related discharge and charge voltages are also recorded. The RtE is calculated by modified equation, which excludes the acid‐base difference.

As discussed in the equation part: $\mathrm{Rt}E_{\mathrm{modi}\mathrm{fied}}=\frac{\mathrm{Disc}\mathrm{harge}\;\mathrm{volt}\mathrm{ag}e_{\mathrm{modi}\mathrm{fied}}}{\mathrm{Char}\mathrm{ge}\;\mathrm{volt}\mathrm{ag}e_{\mathrm{modi}\mathrm{fied}}}=\frac{\mathrm{Disc}\mathrm{harge}\;\mathrm{volt}\mathrm{ag}e_{\mathrm{test}}\;-\;pH_{\mathrm{diff}}^{\mathrm{disc}\mathrm{harge}}\;\times \;0.059}{\mathrm{Char}\mathrm{ge}\;\mathrm{volt}\mathrm{ag}e_{\mathrm{test}}\;-\;pH_{\mathrm{diff}}^{\mathrm{char}\mathrm{ge}}\;\times \;0.059}=\frac{1.66\;-\;\eta_{\mathrm{ORR}}^{\mathrm{acid}}\;-\;IR_{d}\;-\;\eta_{\mathrm{mem}}}{1.66+\eta_{\mathrm{OER}}^{\mathrm{base}}+IR_{c}}$. The discharge voltage is related to the overpotential of ORR in acid medium, the internal resistance, and potential on membrane. The charge voltage is related to the overpotential of OER in alkaline medium and the internal resistance. The conductivities of electrolytes before and after test are characterized by a resistivity tester. The conductivities of pristine alkaline and acidic electrolytes are 0.53 and 0.82 S cm^−1^, respectively. After a 300 h test, the conductivities are 0.22 S cm^−1^ (alkaline cell) and 0.31 S cm^−1^ (acidic cell). The internal resistance after 300 h is characterized by EIS, as shown in Figure S27. The internal resistance increases from 13.81 Ω (Figure S26) to 28.45 Ω, which is not the significant proportion for voltages at the current density of 2 mA cm^−2^. The charge transfer resistance increases a lot, indicating the sluggish kinietics for ORR. This is caused by the severe crossover of H^+^ and OH^−^ after 300 h. Figure 6e shows the relationship between pH values in different cells with their corresponding charge and discharge voltage_modified_. The charge voltage increases slowly with the decreased pH value in the range of 14.771 (0 h) to 14.362 (200 h). Meantime, the modified discharge voltage decreases slowly in the pH range of −0.665 (0 h) to −0.121 (140 h). Then, the ORR in acidic medium faces slow kinetics with increased pH value from −0.121. The overpotentials sharply increases, which is reflected by the decreased discharge voltage.

The decoupled design ensures the stability of the catalyst, when excluding the acid‐base difference, the RtE still shows huge difference. Figure 6f illustrates the relationship between pH difference and RtE calculated by modified equation. The RtE in the range of pH difference between 14.673 and 15.436 is over 47%, indicating the efficient usage of acid‐base difference. The efficient ORR and OER kinetics should be considered during long‐term operation, which are related to pH values in acidic and alkaline cells. Then, the pH difference, as a describer, can help us find the range of acid‐base difference for efficient battery performance, maintaining the asymmetric battery with high RtE. To confirm the efficient range of pH difference, two combinations of electrolytes are used in the DAZAB after 300 h test, as listed in Table S7. The discharge and charge voltages are also listed along with the modified RtE. A refreshment of electrolytes in the efficient range of pH difference could quickly recover the battery performance with a high discharge voltage and a modified RtE over 49%, as shown in Figure S28. The zinc amounts before test and after 300 h test are also characterized by ICP, as shown in Table S8. The Zn^2+^ amount in the whole electrolytes decreases, which is due to the decreased concentration of OH^−^ in alkaline cell. Zn^2+^ then precipitates as zinc oxide, which is also observed in the bottom of the alkaline cell. The morphology of PES/SA membrane after 300 h test is characterized by SEM. The PES/SA membrane shows the little cracking morphology on the alkaline side (Figure S29a), and the acid side still shows the porous structure (Figure S29b), indicating the remarkable chemical stability against acid and alkaline electrolyte.

Based on the pH‐dynamic influence, changing the electrolyte every regular time would be a suitable strategy to make the battery operate with high discharge voltage and efficiency. Electrolytes are refreshed by 6 M KOH with 0.2 M Zn(OAc)_2_ and 3 M H_2_SO_4_ every 80 h to maintain the efficient acid‐base difference with the pumps (Figure S30). The pump only operates for 1 min each time when refresh the electrolyte. The cycle curves of DAZAB of Co/NS‐C as air electrode at current density of 2 mA cm^−2^ are illustrated along with the inserted figures of chosen cycles around 40 and 360 h (Figure 6g). The DAZAB by utilizing the strategy of electrolyte refresh can maintain high discharge voltage over 400 h. The DAZAB with Co/NS‐C as air electrode also undergoes the cycles at a current density of 1 mA cm^−2^ and maintain high discharge voltage over 500 h (Figure S31). The electrolytes are refreshed every 100 h. The detailed parameters of battery configuration and measurements are listed in Table S9. Many reported works related to AZAB have also been summarized in Table S10. However, the parameters related to contribution from acid‐base difference is not included. The pH‐dynamic influence is quite important, which should be considered, and the modified equation of RtE can be used for standard comparison.

## 3 Conclusions

In summary, a DAZAB is developed based on a separated charge/discharge architecture. The separated ORR and OER reactions in different electrolytes guarantee the stability of catalyst. Leveraging this decoupled design, the influence of pH dynamics on battery performance is systematically investigated. Furthermore, the contribution of acid‐base difference in battery performance is also explored, and a modified RtE equation is developed, which is suitable for battery at partial capacity in a half‐open system with stable discharge and charge platforms. A novel membrane enabling efficient ion transportation is fabricated using a PES structure and SA crosslinking channels via simple, low‐cost strategies. Additionally, a non‐noble metal catalyst comprising Co nanocrystals embedded in N and S doped carbon framework is prepared and used in an acidic medium, becoming the potential candidate of Pt/C for ORR. The S doping in the catalyst changes the electrons distribution of active sites synergistically. The DAZAB unlocks the stable catalyst strategy and can achieve a high energy density of 1322 Wh kg_Zn_^−1^ at current density of 2 mA cm^−2^ with the as‐prepared catalyst and membrane. The DAZAB can maintain a high discharge platform over 400 h at a current density of 2 mA cm^−2^ with a strategy of electrolyte refreshment based on the study of pH‐dynamic influence. The innovative design of the decoupled battery, the synthesis of low‐cost membrane and Co/NS‐C catalyst, and the discussion of pH‐dynamic influence open new avenues for the future development of high‐efficiency and cost‐effective energy conversion and storage devices.

## Author Contributions

**Yeshu Tan**: conceptualization, data curation, methodology, investigation, validation, formal analysis, visualization, writing – original draft. **Ruinan Wang**: data curation, formal analysis, visualization, methodology, software, validation, investigation. **Jiawen Huang**: formal analysis, visualization, investigation. **Runzhe Chen**: resources, formal analysis, investigation. **Jiaxin Yuan**: formal analysis, visualization, writing – review and editing. **Siyuan Zhao**: formal analysis, investigation, writing – review and editing. **Meng Ni**: conceptualization, writing – review and editing, project administration, supervision, resources, funding acquisition, formal analysis.

## Conflicts of Interest

The authors declare no conflicts of interest.

## Supporting information




**Supporting File 1**: anie72745‐sup‐0001‐SuppMat.docx.

## Data Availability

Supporting Information is available. All data or materials are available from the corresponding author upon reasonable request.
