# Supplementary material for: A Decoupled Cycling Architecture of Asymmetric Zinc‐Air Battery Unlocks Stable Catalyst Strategy and pH‐Dynamic Influence
Source: Angew Chem Int Ed Engl. 2026 May 19;65(29):e8502321. doi: 10.1002/anie.8502321 (PMC13360798; doi:10.1002/anie.8502321)
Supplement: Supplementary file 1 — Supporting File 1: anie72745‐sup‐0001‐SuppMat.docx. [file ANIE-65-e8502321-s001.docx]

Supplementary Information

**A Decoupled Cycling Architecture of Asymmetric Zinc-air Battery Unlocks Stable Catalyst Strategy and pH-dynamic Influence**

Yeshu Tan^1^, Ruinan Wang^1^, Jiawen Huang^1^, Runzhe Chen^2^, Jiaxin Yuan^1^, Siyuan Zhao^3^, Meng Ni^1^*

^1^Department of Building Environment and Energy Engineering, Research Institute for Sustainable Urban Development (RISUD) and Research Institute for Smart Energy (RISE), The Hong Kong Polytechnic University, Hung Hom, Kowloon, Hong Kong, 999077 China

^2^College of Materials and Chemical Engineering, Minjiang University, Fuzhou, Fujian, China

^3^Department of Life Sciences, Faculty of Science and Technology, Beijing Normal-Hong Kong Baptist University, Zhuhai, Guangdong 519087, China

**Discussion of round-trip efficiency**

The modified pH-dynamic round-trip efficiency regarding to the different types of zinc-air batteries was developed as standard benchmark for pH-decoupling battery system. The pH-dynamic influence and the voltage caused by acid-base difference were considered. The equation was suitable for battery on partial capacity in half-open system with stable discharge and charge platforms.

For discharge (ORR) and charge (OER) process, the thermotical potential could be described by Nernst Equation:^[1-2]^

$E=E^{0}-\frac{RT}{nF}\ln Q=E^{0}-\frac{RT}{nF}\ln\frac{a_{Red}}{a_{Ox}}$ (1)

E is the electrode potential under given conditions.

E^0^ is the standard electrode potential.

R is the ideal gas constant.

T is the temperature in kelvins.

n is the number of electrons transferred.

F is the Faraday constant.

Q is the reaction quotient.

a_red_ is the activity of the reduced form.

a_Ox_ is the activity of the oxidized form.

For the ORR/OER process in acidic medium, the reaction is listed as below:

O_2_ + 4H^+^ + 4e^−^ = 2H_2_O (2)

By using the Nernst equation:

$E=E^{0}-\frac{RT}{nF}\ln\frac{\left[ H_{2}O \right]^{2}}{\left[ H^{+} \right]^{4}\left( PO_{2} \right)}$ (3)

Where at room temperature, K is 298.15 K, R is 8.314 J/(mol⋅K), F is 96485 C/mol, n is 4, [H_2_O] is 1, PO_2_ is 1 atm. Then the Nernst equation is transformed to equation with pH value included:

$E=E^{0}-\frac{2.303RT}{F}pH=E^{0}-0.059\times pH$ (4)

E^0^ represents the standard electrode potential, which is 1.23 V (vs. SHE). Then, we can get the final equation:

$E_{ORR}=1.23-0.059\times pH$ (5)

The electrode potential of OER/ORR is highly relied on the pH value. The relationship between electrode potential (OER/ORR) and pH value is illustrated in Fig. S1.

The zinc electrode is used in alkaline medium and the reaction is listed as below:

ZnO+H_2_O+2e^−^ = Zn+2OH^−^ (6)

Use the Nernst equation and standard electrode potential, we can calculate the electrode potential on zinc electrode as listed below:

$E_{Zn}=-0.43-0.059\times pH$ (7)

This is fixed electrode and is engaged with both discharge and charge process in alkaline medium for all batteries of ZAB, AZAB and DAZAB.

Then, we can calculate the theorical cell voltage with two half reactions on cathode and anode. The whole cell voltages include discharge and charge process for ZAB, AZAB and DAZAB are listed in Table S1, Table S2, Table S3, respectively. The tested and modified round-trip efficiencies are also indicated by theoretical equations and it assumes that reactions on zinc electrode are theoretical. Under the same discharge and charge current and duration time, the tested round-trip efficiency can be described as $\frac{{Discharge voltage}_{test}}{{Charge voltage}_{test}}$.

Table S1. Parameters and equations of traditional zinc-air battery (ZAB)

|  | Traditional zinc-air battery (ZAB) |
| --- | --- |
| Zn electrode | Discharge/Charge  Zn+2OH^-^=ZnO+H_2_O+2e^-^ (KOH, pH=14) (E_0_=-0.43-14×0.059=-1.256V (vs. SHE)) |
| ORR/OER electrode | Discharge/Charge  2H_2_O+O_2_+4e^-^ =4OH^-^ (KOH, pH=14) (E_0_=1.23-14×0.059=0.404 V (vs. SHE)) |
| Cell voltage | Discharge/Charge  E_ORR/OER_ - Ez_n_ = 1.66 V (Zn electrode: pH=14; ORR/OER electrode: pH=14) |
| Round-trip efficiency (tested) | Discharge and charge process under same current density and time (assume the pH values are consistent, Zn electrode: pH=14; ORR/OER electrode: pH=14)  Round-trip efficiency (tested) =$\frac{{Discharge voltage}_{test}}{{Charge voltage}_{test}}$  =$\frac{(E_{ORR}^{base}-\eta_{ORR}^{base})-E_{Zn}-IR_{d}}{{(E}_{OER}^{base}+\eta_{OER}^{base})-E_{Zn}+IR_{c}}$=$\frac{1.66-\eta_{ORR}^{base}-IR_{d}}{1.66+\eta_{OER}^{base}+IR_{c}}$ |
|  | Discharge and charge process under same current density and time (pH values are dynamic and the acid-base difference is indicated)  Utilize equation (5) and (7)  Round-trip efficiency (tested) =$\frac{{Discharge voltage}_{test}}{{Charge voltage}_{test}}$  =$\frac{(E_{ORR}^{base}-\eta_{ORR}^{base})-E_{Zn}-IR_{d}}{{(E}_{OER}^{base}+\eta_{OER}^{base})-E_{Zn}+IR_{c}}$=$\frac{1.66-\eta_{ORR}^{base}-IR_{d}+{pH}_{diff}^{discharge}\times0.059}{1.66+\eta_{OER}^{base}+IR_{c}{+pH}_{diff}^{charge}\times0.059}$  The charge and discharge were taken in the same electrolyte, no difference of pH, then  Round-trip efficiency (tested)= $\frac{1.66-\eta_{ORR}^{base}-IR_{d}}{1.66+\eta_{OER}^{base}+IR_{c}}$*.* It was the same formula as described above. |

Table S2. Parameters and equations of asymmetric zinc-air battery (AZAB)

|  | Asymmetric zinc-air battery (AZAB) |
| --- | --- |
| Zn electrode | Discharge/Charge  Zn+2OH^-^=ZnO+H_2_O+2e^-^ (KOH, pH=14) (E_0_=-0.43-14×0.059=-1.256V (vs. SHE)) |
| ORR/OER electrode | Discharge/Charge  2H_2_O=4H^+^+O_2_+4e^-^ (H_2_SO_4_, pH=0) (E_0_=1.23-0×0.059=1.23 V (vs. SHE)) |
| Cell voltage | Discharge/Charge  E_ORR/OER_ - Ez_n_ = 2.486 V (Zn electrode: pH=14; ORR/OER electrode: pH=0) |
| Round-trip efficiency (tested) | Discharge and charge process under same current density and time (assume the pH values are consistent,  Discharge/charge: Zn electrode: pH=14; ORR/OER electrode: pH=0)  Round-trip efficiency(tested) =$\frac{{Discharge voltage}_{test}}{{Charge voltage}_{test}}$  =$\frac{\left( E_{ORR}^{acid}-\eta_{ORR}^{acid} \right)-E_{Zn}-IR_{d}-\eta_{mem}}{{(E}_{OER}^{acid}+\eta_{OER}^{acid})-E_{Zn}+IR_{c}+\eta_{mem}}$=$\frac{2.486-\eta_{ORR}^{acid}-IR_{d}-\eta_{mem}}{2.486+\eta_{OER}^{acid}+IR_{c}+\eta_{mem}}$ |
| pH-dynamic round-trip efficiency (modified) | Discharge and charge process under same current density and time (pH values are dynamic and the acid-base difference is indicated)  Utilize equation (5) and (7)  Round-trip efficiency (tested)=$\frac{{Discharge voltage}_{test}}{{Charge voltage}_{test}}$ =$\frac{\left( E_{ORR}^{acid}-\eta_{ORR}^{acid} \right)-E_{Zn}-IR_{d}-\eta_{mem}}{{(E}_{OER}^{acid}+\eta_{OER}^{acid})-E_{Zn}+IR_{c}+\eta_{mem}}$=$\frac{1.66-\eta_{ORR}^{acid}-IR_{d}-\eta_{mem}+{pH}_{diff}^{discharge}\times0.059}{1.66+\eta_{OER}^{acid}+IR_{c}+\eta_{mem}+{pH}_{diff}^{charge}\times0.059}$  pH_diff_ indicates the extra voltage caused by acid-base difference, which should be excluded for modified round-trip efficiency  Round-trip efficiency (modified)=$\frac{{Discharge voltage}_{modified}}{{Charge voltage}_{modified}}= \frac{{Discharge voltage}_{test}-{pH}_{diff}^{discharge}\times0.059}{{Charge voltage}_{test}-{pH}_{diff}^{charge}\times0.059}$ |

Table S3. Parameters and equations of decoupled asymmetric zinc-air battery (DAZAB)

|  | Decoupled asymmetric zinc-air battery (DAZAB) |
| --- | --- |
| Zn electrode | Discharge/Charge  Zn+2OH^-^=ZnO+H_2_O+2e^-^ (KOH, pH=14) (E_0_=-0.43-14×0.059=-1.256V (vs. SHE)) |
| ORR electrode | Discharge  H_2_O=4H^+^+O_2_+4e^-^ (H_2_SO_4_, pH=0) (E_0_=1.23-0×0.059=1.23 V (vs. SHE)) |
| OER electrode | Charge  4OH^-^ = 2H_2_O+O_2_+4e^-^ (KOH, pH=14) (E_0_=1.23-14×0.059=0.404 V (vs. SHE)) |
| Cell voltage | Discharge  E_ORR_ - Ez_n_ = 2.486 V (Zn electrode: pH=14; ORR electrode: pH=0)  Charge  E_OER_ - Ez_n_ = 1.66 V (Zn electrode: pH=14; OER electrode: pH=14) |
| Round-trip efficiency  (tested) | Discharge and charge process under same current density and time (assume the pH values are consistent;  discharge: Zn electrode: pH=14; ORR electrode: pH=0;  charge: Zn electrode: pH=14; OER electrode: pH=14)  Round-trip efficiency (tested) =$\frac{{Discharge voltage}_{test}}{{Charge voltage}_{test}}$  =$\frac{\left( E_{ORR}^{acid}-\eta_{ORR}^{acid} \right)-E_{Zn}-IR_{d}-\eta_{mem}}{{(E}_{OER}^{base}+\eta_{OER}^{base})-E_{Zn}+IR_{c}}$=$\frac{2.486-\eta_{ORR}^{acid}-IR_{d}-\eta_{mem}}{1.66+\eta_{OER}^{base}+IR_{c}}$ |
| pH-dynamic round-trip efficiency (modified) | Discharge and charge process under same current density and time (pH values are dynamic and the acid-base difference is considered)  Utilize equation (5) and (7)  Round-trip efficiency (tested)=$\frac{{Discharge voltage}_{test}}{{Charge voltage}_{test}}$  =$\frac{\left( E_{ORR}^{acid}-\eta_{ORR}^{acid} \right)-E_{Zn}-IR_{d}-\eta_{mem}}{{(E}_{OER}^{base}+\eta_{OER}^{base})-E_{Zn}+IR_{c}}$=$\frac{1.66-\eta_{ORR}^{acid}-IR_{d}-\eta_{mem}+{pH}_{diff}^{discharge}\times0.059}{1.66+\eta_{OER}^{base}+IR_{c}+{pH}_{diff}^{charge}\times0.059}$  pH_diff_ indicates the extra voltage caused by acid-base difference, which should be excluded for modified round-trip efficiency.  Round-trip efficiency (modified)=$\frac{{Discharge voltage}_{modified}}{{Charge voltage}_{modified}}= \frac{{Discharge voltage}_{test}-{pH}_{diff}^{discharge}\times0.059}{{Charge voltage}_{test}-{pH}_{diff}^{charge}\times0.059}$  Due to the charge process is under the same electrolyte, no pH difference.  Round-trip efficiency (modified)=$\frac{{Discharge voltage}_{modified}}{{Charge voltage}_{modified}}= \frac{{Discharge voltage}_{test}-{pH}_{diff}^{discharge}\times0.059}{{Charge voltage}_{test}}$ |

η_ORR_ represents the overpotential of ORR, related to activity of catalyst.

η_OER_ represents the overpotential of OER, related to activity of catalyst.

R_d_ represents the internal resistance of whole system during discharge process.

R_c_ represents the internal resistance of whole system during charge process.

I represents the current.

η_mem_ represents the extra potential related to membrane.

${pH}_{diff}^{discharge}$ represents the pH difference value of two electrodes in discharge cell. The value is positive.

${pH}_{diff}^{charge}$ represents the pH difference value of two electrodes in charge cell. The value is positive.

For rechargeable process of all kinds of zinc-air batteries, the discharge and charge processes are under the same current density and time to ensure the balanced powers. Thus, the round-trip efficiency at one cycle can be described by the discharge potential divided by charge potential. In the discussion, we assume that the reaction on Zn electrode is theoretical.

For traditional zinc-air battery (ZAB), the whole cell voltage is 1.66 V for both discharge and charge process. The round-trip efficiency is indicated by equation: $\frac{{Discharge voltage}_{test}}{{Charge voltage}_{test}}=\frac{1.66-\eta_{ORR}^{base}-IR_{d}}{1.66+\eta_{OER}^{base}+IR_{c}}$. η_ORR_ and η_OER_ represent the overpotential during reaction. During discharge, the overpotential must be removed for effective voltage, and for charge part, the overpotential should be added for real voltage. So do the extra voltages reflected by internal resistance, indicated by IR. The practical discharge and charge voltage rely on the activities of catalysts for OER and ORR. The internal resistance is also important. The tested round-trip efficiency at one point for ZAB is:$\frac{{Discharge voltage}_{test}}{{Charge voltage}_{test}}$.

While for asymmetric zinc-air battery (AZAB), the whole cell voltage is 2.486 V for both discharge and charge process. The round-trip efficiency is indicated by$\frac{{Discharge voltage}_{test}}{{Charge voltage}_{test}}= \frac{2.486-\eta_{ORR}^{acid}-IR_{d}-\eta_{mem}}{2.486+\eta_{OER}^{acid}+IR_{c}+\eta_{mem}}$, which assumes that pH values in different electrolytes are consistent (Zn electrode: pH=14; ORR/OER electrode: pH=0). The increased discharge voltage is due to the introduction of chemical energy (acid-base difference) from pH 14 to pH 0. There is a voltage difference of 14×0.059=0.826 V. Meantime, the charge voltage is also increased due to the acid-base difference. The introduction of acid-base difference both increase the discharge and charge voltages; the round-trip efficiency is still limited by the activities of catalysts for OER and ORR. Meantime, extra potential is needed for membrane (η_mem_). Most importantly, the equation of round-trip efficiency above does not consider that the pH values are dynamic in the system and does not describe the voltage related to pH (acid-base difference). So, the round-trip efficiency for this kind of asymmetric system by utilizing pH decoupling electrolytes is indicated by equation:$\frac{{Discharge voltage}_{test}}{{Charge voltage}_{test}}=\frac{\left( E_{ORR}^{acid}-\eta_{ORR}^{acid} \right)-E_{Zn}-IR_{d}-\eta_{mem}}{{(E}_{OER}^{acid}+\eta_{OER}^{acid})-E_{Zn}+IR_{c}+\eta_{mem}}$=$\frac{1.66-\eta_{ORR}^{acid}-IR_{d}-\eta_{mem}+{pH}_{diff}^{discharge}\times0.059}{1.66+\eta_{OER}^{acid}+IR_{c}+\eta_{mem}+{pH}_{diff}^{charge}\times0.059}$. The pH difference indicates the extra voltage caused by acid-base difference. The tested round-trip efficiency for AZAB should exclude the voltage caused by acid-base difference as standard benchmark. The modified pH-dynamic round-trip efficiency at one point for AZAB is:$\frac{{Discharge voltage}_{modified}}{{Charge voltage}_{modified}}= \frac{{Discharge voltage}_{test}-{pH}_{diff}^{discharge}\times0.059}{{Charge voltage}_{test}-{pH}_{diff}^{charge}\times0.059}$.

For decoupled asymmetric zinc-air battery (DAZAB), the whole cell voltages are 2.486 V and 1.66 V for discharge and charge, respectively. The tested round-trip efficiency is indicated by equation: $\frac{{Discharge voltage}_{test}}{{Charge voltage}_{test}}=\frac{2.486-\eta_{ORR}^{acid}-IR_{d}-\eta_{mem}}{1.66+\eta_{OER}^{base}+IR_{c}}$ (assume the pH values are consistent; discharge: Zn electrode: pH=14; ORR electrode: pH=0; charge: Zn electrode: pH=14; OER electrode: pH=14).

From the equation, we can observe that tested round-trip efficiency can be over 100%, because the decoupled discharge (acid) and charge (alkaline) process brings the increased discharge voltage and ensures normal charge voltage. For standard comparison of round-trip efficiency, the extra voltage caused by acid-base difference should be excluded. The round-trip efficiency for this kind of asymmetric system by utilizing pH decoupling electrolytes is further illustrated: $\frac{{Discharge voltage}_{test}}{{Charge voltage}_{test}}=\frac{\left( E_{ORR}^{acid}-\eta_{ORR}^{acid} \right)-E_{Zn}-IR_{d}-\eta_{mem}}{{(E}_{OER}^{base}+\eta_{OER}^{base})-E_{Zn}+IR_{c}}=\frac{1.66-\eta_{ORR}^{acid}-IR_{d}-\eta_{mem}+{pH}_{diff}^{discharge}\times0.059}{1.66+\eta_{OER}^{base}+IR_{c}+{pH}_{diff}^{charge}\times0.059}$*.* The pH difference indicates the extra voltage caused by acid-base difference. The modified pH-dynamic round-trip efficiency at one point for DAZAB is:$\frac{{Discharge voltage}_{modified}}{{Charge voltage}_{modified}}= \frac{{Discharge voltage}_{test}-{pH}_{diff}^{discharge}\times0.059}{{Charge voltage}_{test}-{pH}_{diff}^{charge}\times0.059}$. Because two electrodes during charge process are in the same electrolyte, no pH difference is included in charge process, the equation for DAZAB can be simplified as :$\frac{{Discharge voltage}_{modified}}{{Charge voltage}_{modified}}=\frac{{Discharge voltage}_{test}-{pH}_{diff}^{discharge}\times0.059}{{Charge voltage}_{test}}$.

For AZAB and DAZAB utilizing pH-decoupling electrolytes, the internal parameters related to round-trip efficiency are listed:

AZAB, Round-trip efficiency (tested): $\frac{1.66-\eta_{ORR}^{acid}-IR_{d}-\eta_{mem}+{pH}_{diff}^{discharge}\times0.059}{1.66+\eta_{OER}^{acid}+IR_{c}+\eta_{mem}+{pH}_{diff}^{charge}\times0.059}$

DAZAB, Round-trip efficiency (tested): $\frac{1.66-\eta_{ORR}^{acid}-IR_{d}-\eta_{mem}+{pH}_{diff}^{discharge}\times0.059}{1.66+\eta_{OER}^{base}+IR_{c}+{pH}_{diff}^{charge}\times0.059}$

From the discharge part, we can observe the same formulas. However, in charge part. No extra potential on membrane is needed for DAZAB. Meantime, the overpotentials of OER in acid and alkaline electrolytes are also different. The reaction dynamic in alkaline for OER is more favorite, which indicating lower overpotential.^[3-4]^ Then considering the same pH-decoupling electrolytes, the charge part for AZAB will go through higher voltage than that for DAZAB. The DAZAB has lower charge voltage under the same standards benchmark (exclude acid-base difference).

For the battery system utilizing pH-decoupling electrolytes, the introduction of acid-base difference leads to higher practical discharge voltage. However, for comparison of the round-trip efficiency, the pH-dynamic property and the extra voltage caused by acid-base difference should be considered. Herein, we developed the modified pH-dynamic round-trip efficiency with universal equation to describe the decoupled battery system in a standard benchmark, as indicated by equation:$\frac{{Discharge voltage}_{modified}}{{Charge voltage}_{modified}}= \frac{{Discharge voltage}_{test}-{pH}_{diff}^{discharge}\times0.059}{{Charge voltage}_{test}-{pH}_{diff}^{charge}\times0.059}$.

**Experimental section**

**Materials:**

Polyethersulfone (PES, 99%), methylimidazole (2-MIM, 98%) and sodium alginate (SA) were purchased from Macklin. L- methionine (99%) was purchased from Aladdin. Potassium hydroxide (KOH, 90%), cobalt nitrate hexahydrate (Co(NO_3_)_2_·6H_2_O, 99%), zinc sulfate (ZnSO_4_, 99%) and zinc acetate (Zn(OAc)_2_, 99%) were purchased from Macklin. Commercial platinum/carbon (20% Pt/C), methanol (99%) and N-methyl-2-pyrrolidone (NMP) were purchased from Alfa Aesar. Polyvinyl pyrrolidone (molecular weight 220000, PVP) was purchased from Macklin. RuO_2_ (99%) and Nafion 117 solution were purchased from sigma. All chemical reagents were used in the experiments directly without further treatment.

**Preparation of PES membrane:**

PES membrane was fabricated based on solvent-nonsolvent exchange strategy, which induces phase separation and forms a porous structure. Firstly, 5 g PES was dissolved in 50 g NMP. The solution was stirred for 24 h to form the homogeneous solution. Secondly, the solution was poured into the costumed module with thickness of 1 mm. Thirdly, the module was put into the DI water quickly and remained for 24 h. Finally, the prepared PES membrane was washed by water several times and dried in oven at 60 ^o^C for 24 h.

**Preparation of PES/SA membrane**:

PES/SA membrane was fabricated under the same strategy. 10 g of the homogeneous solution was mixed with 3 g of sodium alginate powder. Then the mixture was grinded by ball milling machine for 3 h. The mixed solution became turbid and was poured into the module immediately. Finally, the module was put int the 0.1 M ZnSO_4_ solution for 24 h. Then the PES/SA membrane was collected and washed, finally dried at 60 ^o^C for 24 h. The PES/SA membrane without Zn^2+^ crosslinking was fabricated under the same procedure by replacing the 0.1 M ZnSO_4_ solution with DI water.

**Preparation of SA membrane**:

SA membrane was fabricated by dissolving 3 g SA into 10 g of DI water. Then 1 ml of 0.1 M ZnSO_4_ was added into the solution for stirring of 1 h. The precipitation was collected by centrifugation and then pressed on the glass. Finally, the flat membrane was dried in oven under 60 ^o^C for 24 h. The SA membrane was fragile which needs careful operation.

**Preparation of ZIF-67 and Co/N-C:**

Typically, for synthesis of ZIF-67, cobalt nitrate hexahydrate (Co(NO_3_)_2_·6H_2_O) (1.164 g) was dissolved in 40 ml of methanol as solution A with 600 mg PVP, then 2.627 g 2-methylimidazole (2-MIM) was dissolved in 40 ml DI water as solution B. Then, solution B was poured into solution A slowly under severe stirring conditions. The mixture was stirred for 2 h at room temperature. The ZIF-67 precipitation was collected by centrifugation and washed with methanol three times. The purple powder was dried in oven at 60 ^o^C for 24 h. Then the dried ZIF-67 powder was put into graphite boat fixed in the joule heating machine (CIS-JH4.0 joule heating device). The chamber was fulfilled with nitrogen and heated to 800 ^o^C. The temperature increasing process lasted less than 3 s and kept at 800 ^o^C for 10 s. The black powder was collected and put into 2M HCl under ultrasound for 30 mins and placed for 4 h for acid treating to remove those Co nanoparticles on the carbon surface. Finally, Co/N-C was washed and collected by centrifugation and dried in oven at 60 ^o^C for 24 h.

**Preparation of ZIF-67/S and Co/NS-C**:

ZIF-67/S was fabricated under the same procedures with extra adding of 0.597 g methionine in solution B. The pH of solution B was tuned by 1M KOH and matched the value in the solution B in synthesis of ZIF-67. Then ZIF-67/S was prepared by mixing solution A and solution B for 2 h under stirring at room temperature. The purple precipitation was collected by centrifugation and washed with methanol three times. The sample was dried in oven at 60 ^o^C for 24 h. For synthesis of Co/NS-C, the carbonization procedure was the same in joule heating machine. Finally, the sample was also put into 2M HCl for acid treatment to get the Co embedded in N and S doped carbon. Co/NS-C was collected and washed several times and dried in oven at 60 ^o^C for 24 h.

**Preparation of Co(mix)/NS-C**:

The Co(mix)/NS-C powder was prepared based on the Co/NS-C without acid treatment.

**Preparation of NS-C:**

2.627 g of 2-methylimidazole and 0.597 g methionine were mixed by ball milling. Then the powder was carbonized by joule heating machine. Then the black powder was collected as NS-C.

**Preparation of catalyst on RRDE.**

Typically, 5 mg commercial 20% Pt/C powder, 5 mg Co/N-C, or 5 mg Co/NS-C was dispersed in mixed solution with 350 μl DI water, 135 μl ethanol and 15 μl Nafion solution. Then the solution was under ultrasonication for 1 h. 15 μl of solution was dropped on the disk area of rotating ring-disk electrode (RRDE) with an area of 0.2475 cm² and dried under 50 ^o^C with slow rotation.

**Preparation of air electrode of Pt/C, Co/N-C and Co/NS-C**:

In a typical preparation process, 20 mg commercial Pt/C (20% Pt) powder was dispersed in solution with 2 mL of DI water, 7.8 mL of ethanol and 200 μL Nafion solution, then 1 mL of the mixed solution was sprayed and dried on 1 cm^2^ of carbon paper to fabricate the Pt/C air electrode. The loading was weighted before and after spraying and drying. The loading was about 1.2 mg cm^-2^. At the same time, Co/N-C or Co/NS-C was prepared under the same procedure to get the air electrode.

**Preparation of bifunctional air electrode:**

In a typical preparation process, 20 mg commercial Pt/C (20% Pt) powder was mixed with 6 mg RuO_2_ in solution with 2 mL of DI water, 7.8 mL of ethanol and 200 μl Nafion solution, then 1 mL of the mixed solution was sprayed and dried on 1 cm^2^ of carbon paper to fabricate the Pt/C air electrode. The loading was about 1.5 mg cm^-2^.

**Activation of stainless-steel (SS) electrode:**

A piece of SS electrode was firstly cleaned by ethanol and DI water and then used as a working electrode, while a Hg/HgO electrode and a carbon rod were used as the reference and the counter electrode, respectively. 1 M KOH was used as an electrolyte solution. CHI760E electrochemical workstation was used to perform cyclic voltammetry (CV) activation. The potential window was from -1 V to 1 V (Hg/HgO) with scan rate of 50 mV s^-1^; The CV cycles were performed for ten times.

**Assemble of decoupled asymmetric zinc-air battery (DAZAB):**

The DAZAB was assembled by combined a zinc electrode, alkaline electrolyte, a piece of activated SS mesh, a PES/SA membrane, acidic electrolyte and an air electrode subsequently. A zinc electrode, alkaline electrolyte, a PES/SA membrane, acidic electrolyte and an air electrode were asymmetric discharge cell. A zinc electrode, alkaline electrolyte and a piece of activated SS mesh were alkaline charge cell. 6 M KOH mixed with 0.2 M Zn(OAc)_2_ solution was used as alkaline electrolyte and 3 M H_2_SO_4_ solution was used as acidic electrolyte.

**Calculations for crossover of ions:**

The crossover of ions is indicated by equation as below:^[5]^

$$J=\frac{dc}{dt}\times\frac{V}{A}$$

Where dc/dt represents the concentration difference per unit time, V represents the volume of the cell, which is 8 mL, A is the area of membrane, which is 1.13 cm^2^.

**Calculations H_2_O_2_ selectivity and electron transfer number:**^[6]^

The H_2_O_2_ selectivity is determined based on equation: H_2_O_2_ %=$200\times\frac{Iring/N}{IDisk+IRing/N}$

The electron transfer number is followed by equation: n=$4\times\frac{I_{disk}}{I_{disk}+I_{ring}/N}$

where I_Ring_ is the ring current, I_Disk_ is the disk current and N is the collection efficiency. The experimental N is determined according to the reported work, which is 0.375.^[7]^

**Calculations of capacity and energy density for battery:**

DAZAB is a half-open system, the utilization of O_2_ is not included into the calculation of capacity, because it is almost infinite in the air. Thus, the capacity of the DAZAB is relied on the zinc electrode. The reacted zinc on electrode was about 0.357 g (Assume that the zinc on electrode in working area was totally reacted). the capacity can be calculated from followed equation:^[8]^ $Q=\frac{I\times t}{m}$.

I represents the current (mA), t is the time (h), m is the weight of zinc.

The calculate value of capacity was 778.7 mAh g_Zn_^-1^ for 2 mA. While the capacity calculated from 1 mA was 627.17 mAh g_Zn_^-1^.

Based on the continuous discharge curves in Fig. 6c, the energy density was calculated through integration and the weight of zinc was considered as followed by equation:^[8]^ $E=\frac{I\times\int_{0}^{t} V\left( t \right)dt}{m}$.

V represents the discharge voltage (V). For 2 mA discharge curve, it had a high energy density of 1322 Wh kg_Zn_^-1^. While for 1 mA discharge curve, it had an energy density of 1132 Wh kg_Zn_^-1^.

**Characterization**:

X-ray diffraction (XRD) patterns were obtained by a X-ray diffractometer (Rigaku SmartLab 9kW) with a detected angular range of 5° < 2θ < 80° with 9 kW rotating anode X-ray source (λ~1.54Å) coupling with high-quality semiconductor detector. The surface morphology and elemental dispersion of the as-prepared precursors and samples were characterized by scanning electron microscope (SEM, Tescan MIRA and Tescan VEGA3) and transmission electron microscopy (TEM JEOL and JEM-2100). The chemical states of different elements were evaluated by X-ray photoelectron spectroscopy (XPS, Shimadzu/Kratos AXIS SUPRA+) analysis. Date analysis of XPS results were processed by Casa XPS with the calibration of C 1s main peak at 284.8 eV. The chemical group signals were acquired by Fourier transform infrared spectrophotometer (FTIR, Thermo Fisher Scientific Nicolet iN10). X-ray absorption fine structure (XAFS) spectroscopy was carried out using the RapidXAFS 2M (Anhui Absorption Spectroscopy Analysis Instrument Co., Ltd.) by transmission mode at 20 kV and 40 mA, the Si (533) spherically bent crystal analyzer with a radius of curvature of 500 mm was used for Co. The samples were prepared by mixing and grinding with cellulose to pallets under compression. The obtained data were processed with the ATHENA software, and the energy shift was calibrated based on the feature of incident beam current (I_0_). The concentration of H^+^ and OH^-^ are tested by pH meter (thermos scientific PH111), while Co^2+^, Zn^2+^ and K^+^ are evaluated by Inductively Coupled Plasma (ICP-OES, Agilent 5110). SO_4_^2-^ ions are tested by Ion Chromatography (IC, DIONEX AQUION RFIC). The conductivity of electrolyte was characterized by Resistivity Tester (ST2242).

**Electrochemical Performance**:

Battery performance tests were characterized by NEWARE battery testing systems for cycling stability and step discharge curves. For cycles test of DAZAB, alkaline cell of a zinc electrode and a SS electrode was tested for charge. The charge process was set by cycling of 10 mins pending process and 10 mins charging process. Asymmetric cell of a zinc electrode and an air electrode was tested for discharge. The discharge process was set by cycling of 10 mins discharge process and 10 mins pending process. Electrochemical performances such as cyclic voltammetry (CV), linear sweep voltammetry (LSV) and electrochemical impedance spectroscopy (EIS) were measured by a CHI760F electrochemical workstation. EIS was performed with frequencies from 0.1 to 100,000 Hz with an amplitude of 5 mV. Carbon paper was used as electrodes in the EIS test. In a three-electrode test for ORR, a Ag/AgCl electrode and a graphite electrode were used as standard and counter electrode, respectively. RRDE was used as working electrode and N_2_ or O_2_ saturated 0.1 M H_2_SO_4_ was used as electrolyte. ORR test was carried out in the N_2_ or O_2_ saturated electrolyte between 0 V and 1 V vs. Ag/AgCl at a scan rate of 10 mV s^−1^ under 1600 rpm. The ring electrode was set to 1.1 V vs. Ag/AgCl. All potentials were measured based on a Ag/AgCl electrode and were converted to the reversible hydrogen electrode into the potential (RHE) according to the equation E_RHE_ = E_Ag/AgCl_ + 0.197 + 0.059pH. Tafel slopes were determined by fitting the linear regions of the Tafel plots according to the Tafel equation (η = b log(j) + a) by replotting the polarization curves. Durability tests were evaluated by CV measurement. A Hg/HgO electrode was used for OER test and potentials were converted to the reversible hydrogen electrode by equation of E_RHE_ = E_Hg/HgO_ + 0.098 + 0.059pH. All LSV measurements were illustrated without iR compensation.

**Computation methods**

All calculations for systems were based on density functional theory (DFT). The exchange-function was treated based on the same Generalized Gradient Approximation (GGA) in the form of Perdew-Burke-Ernzerhof (PBE), in combination with the DFT-D correction.^[9-10]^ For the optimization of the geometry and lattice size of all structures, the cut-off energy of the plane-wave basis was set to 500 eV and the Brillouin zone integration is performed using 2×2×1 Monkhorts-Pack k-point sampling. And the convergence criteria for the ionic relaxation and electronic self-consistent calculation were set to 0.05 eV Å-1 and 2×10^-5^ eV, respectively.

In this work, the supercell of Co was established and the slice (111) was constructed for the surface and a vacuum of 15 Å was added along the vertical direction perpendicular to the surface. Then a carbon layer doped with N or N/S was loaded on the (111) surface of Co, serving as the base structure for the calculation. And a pure carbon structure with the same size and surface doping as the sample was established as a control group for the calculation.

With the reaction formula of O_2_ + 2H_2_ → 2H_2_O, the standard reaction energy is -4.92 eV. The DFT elementary reactions are listed as below:^[11-12]^

** + O_2_ — *OO*

**OO + H+ +e- — *OOH*

**OOH + H+ +e- — *O + H_2_O (g)*

**O + H+ +e- — *OH*

**OH + H+ +e- — * + H_2_O (g)*

The corresponding energy calculation formula are listed:

*△E_*_ = 0 eV*

*△E_*OO_ = E_*OO_ - E_*_ - E_O2_*

*△E_*OO_ = E_*OOH_ - E_*_ - E_O2_ - E_H+_*

*△E_*O_ = E_*O_ + E_H2O_ - E_*_ - E_O2_ - 2E_H+_*

*△E_*OH_ = E_*OH_ + E_H2O_ - E_*_ - E_O2_ - 3E_H+_*

*△E_*+H2O_ = 2E_H2O_ - E_O2_ - 4E_H+_ =-4.92 eV*


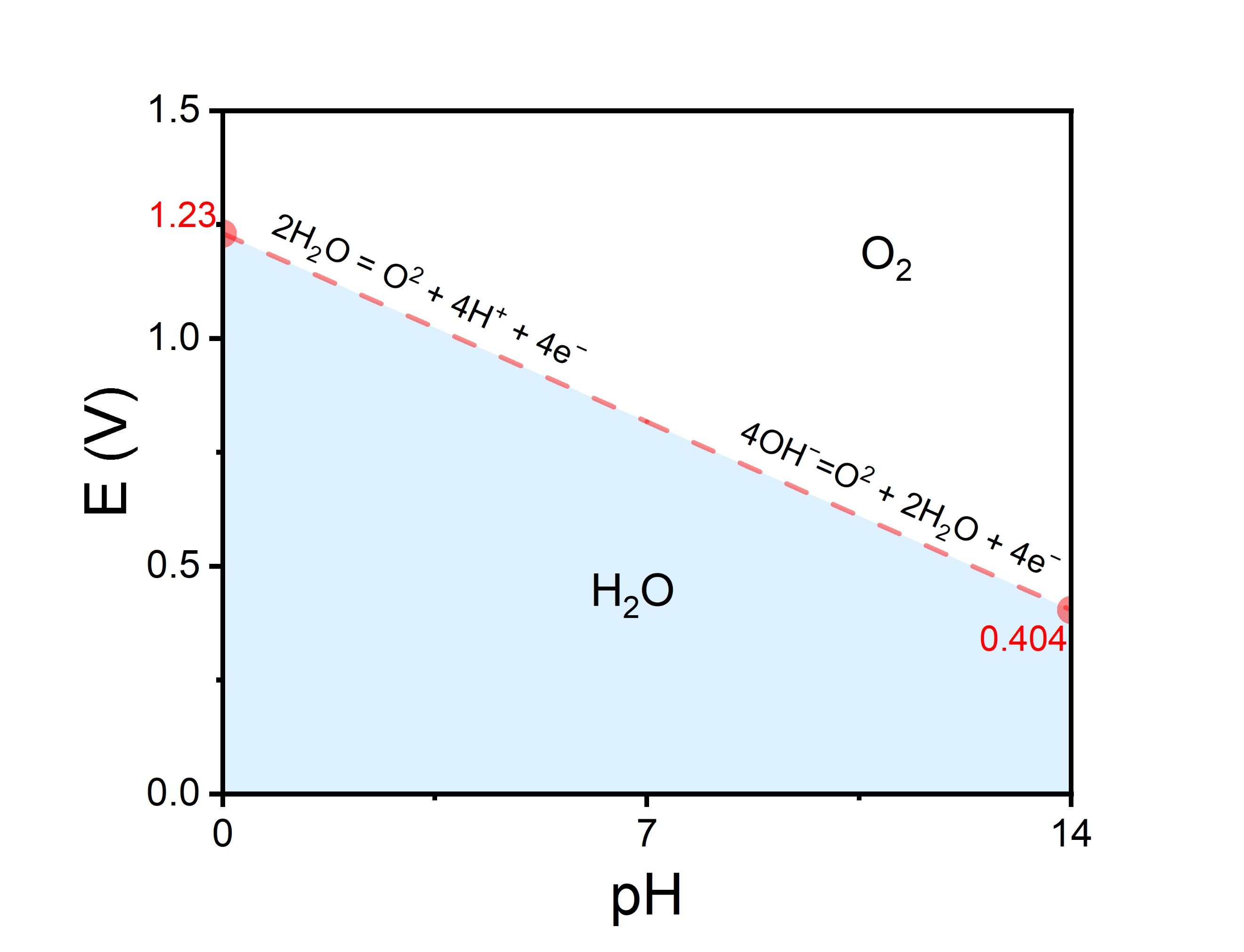


Fig. S1. Relationship between electrode potential of ORR/OER and pH value.


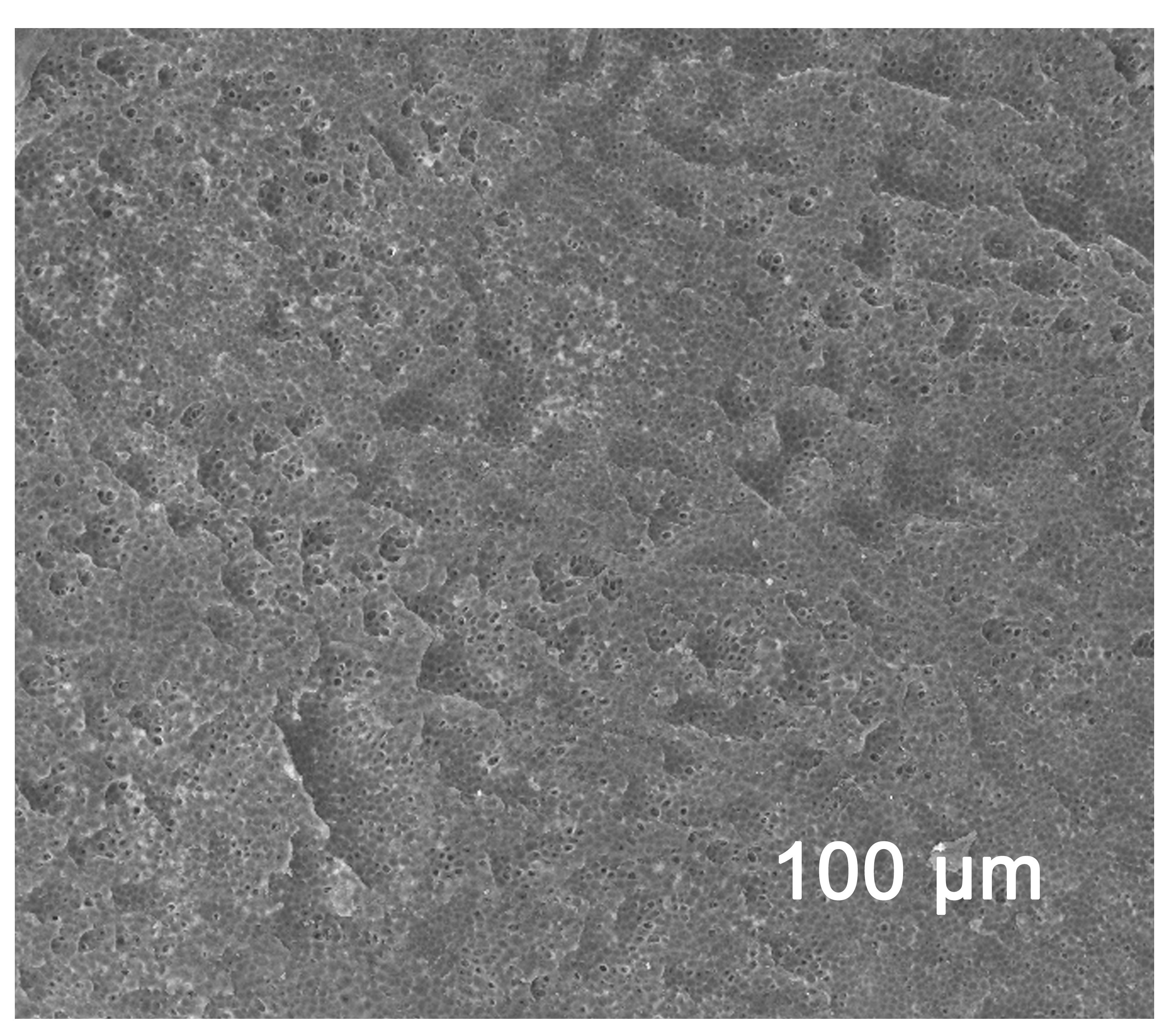


Fig. S2. SEM image of PES membrane.


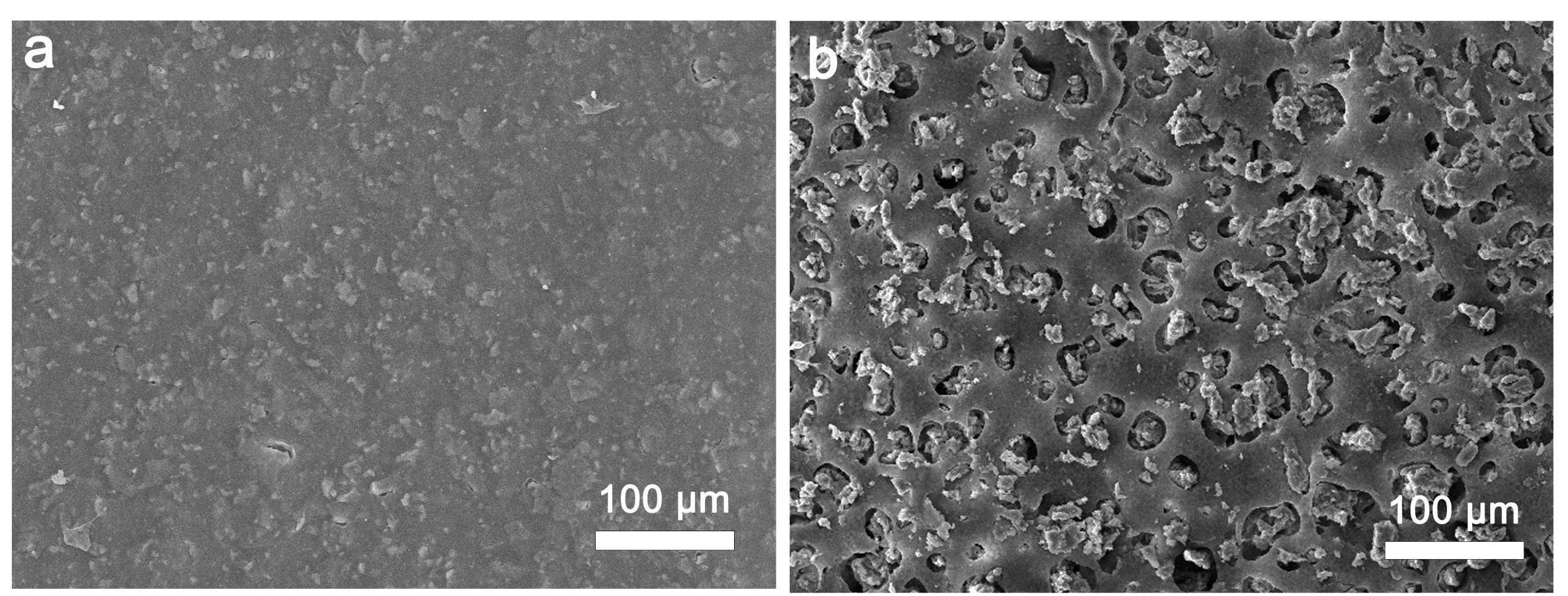


Fig. S3. SEM images of two sides for PES/SA membrane.


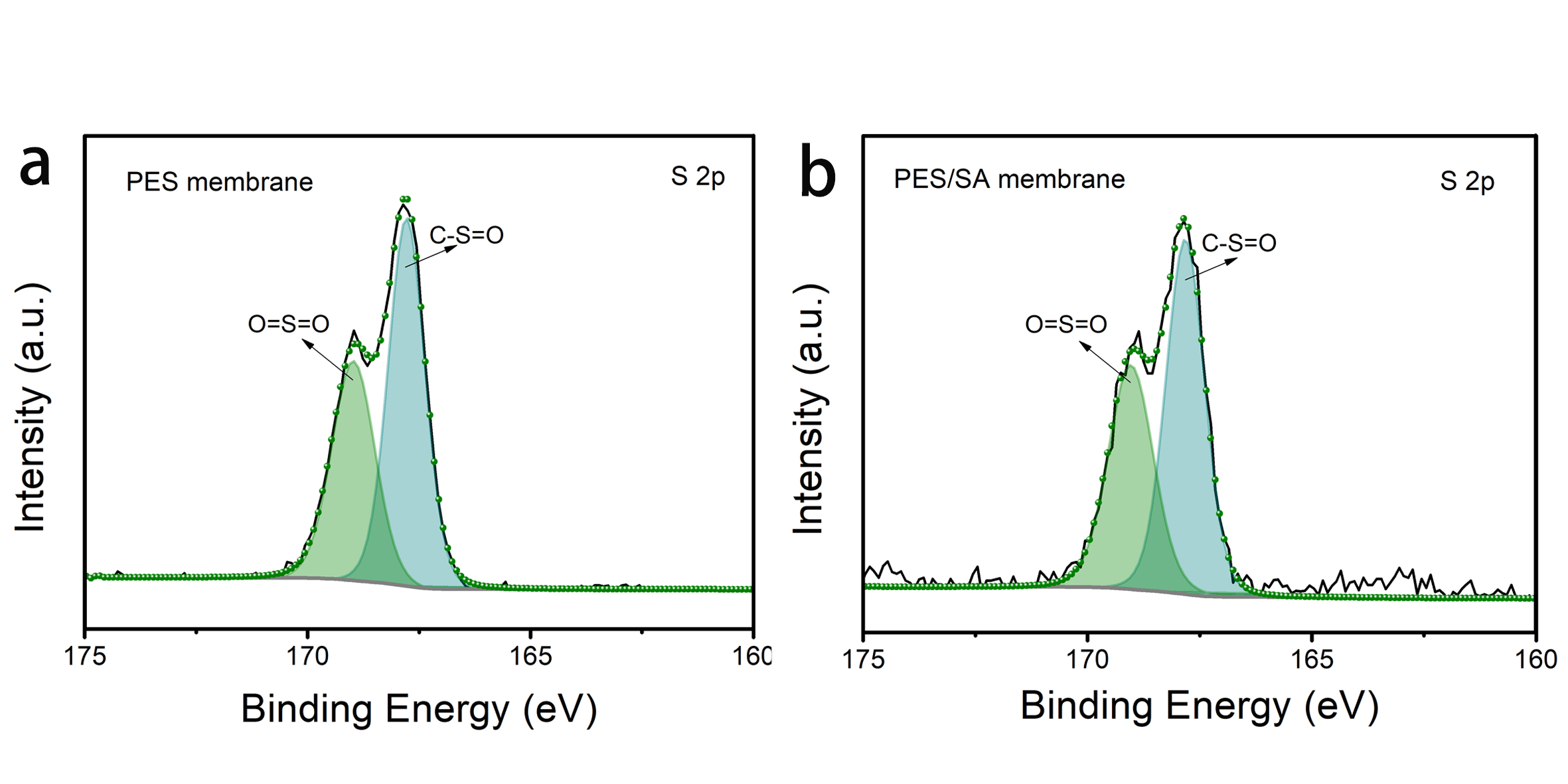


Fig. S4. XPS spectra of S 2p of (a) PES membrane and (b) PES/SA membrane.


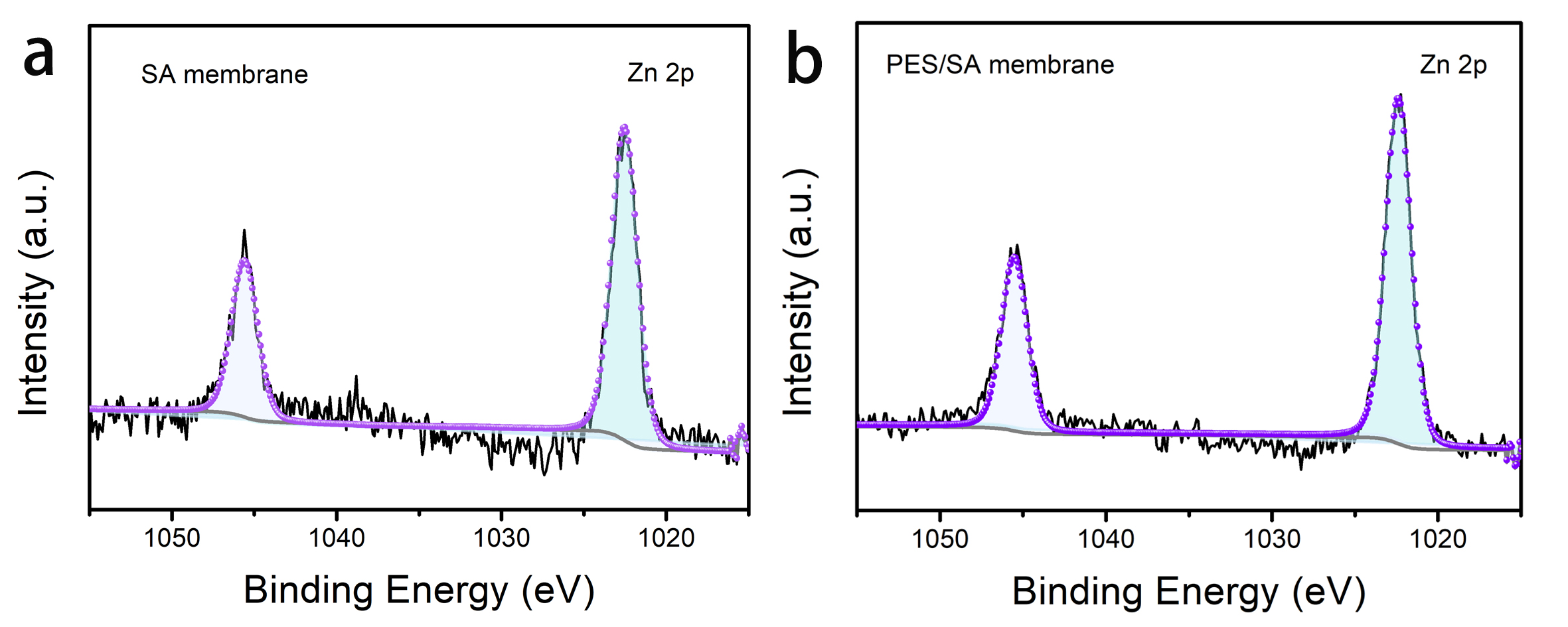


Fig. S5. XPS spectra of Zn 2p of (a) SA membrane and (b) PES/SA membrane.


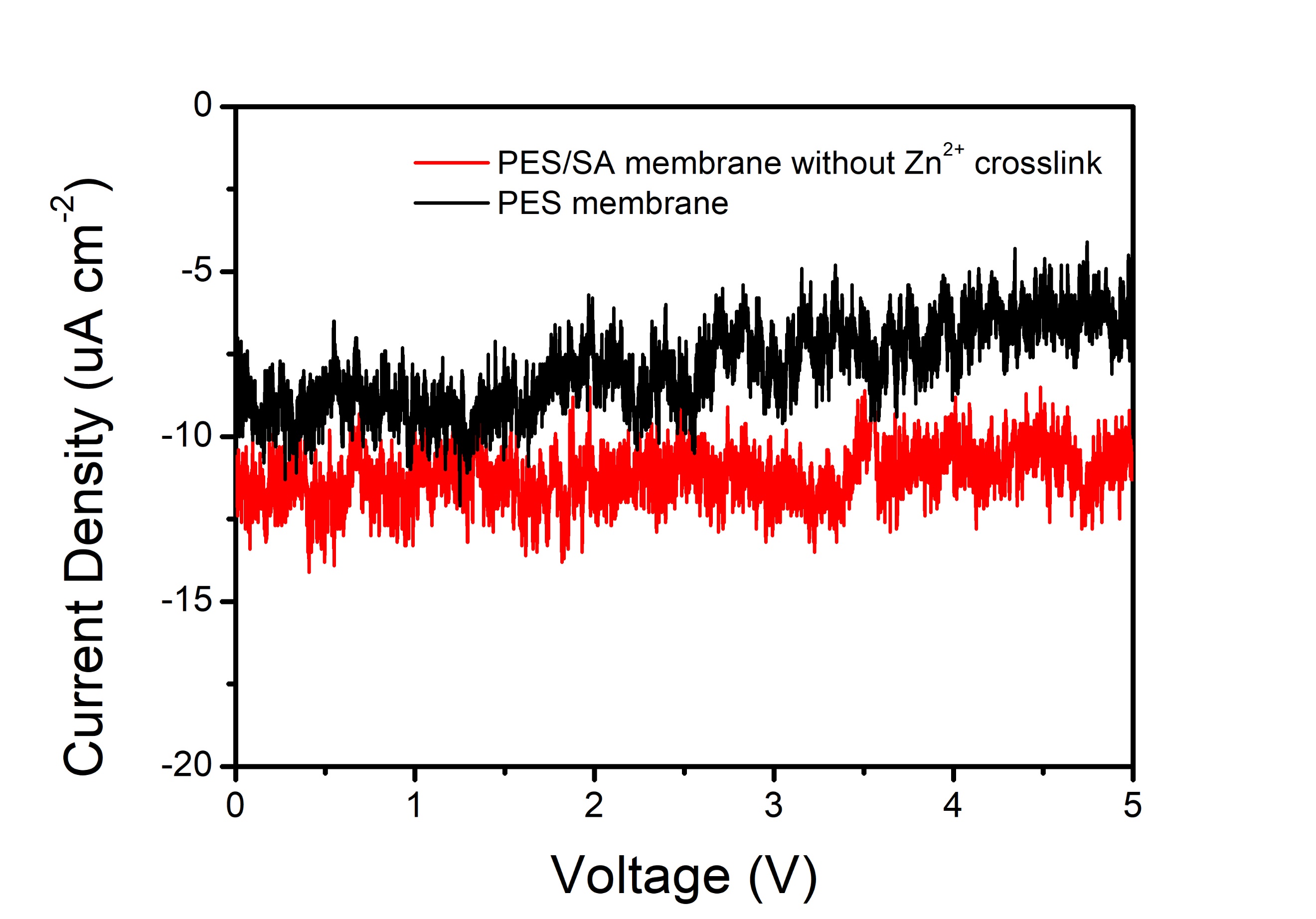


Fig. S6. I-V curves of symmetric test cell (0.5 M H_2_SO_4_) with PES and PES/SA (no Zn^2+^ crosslink) membranes.


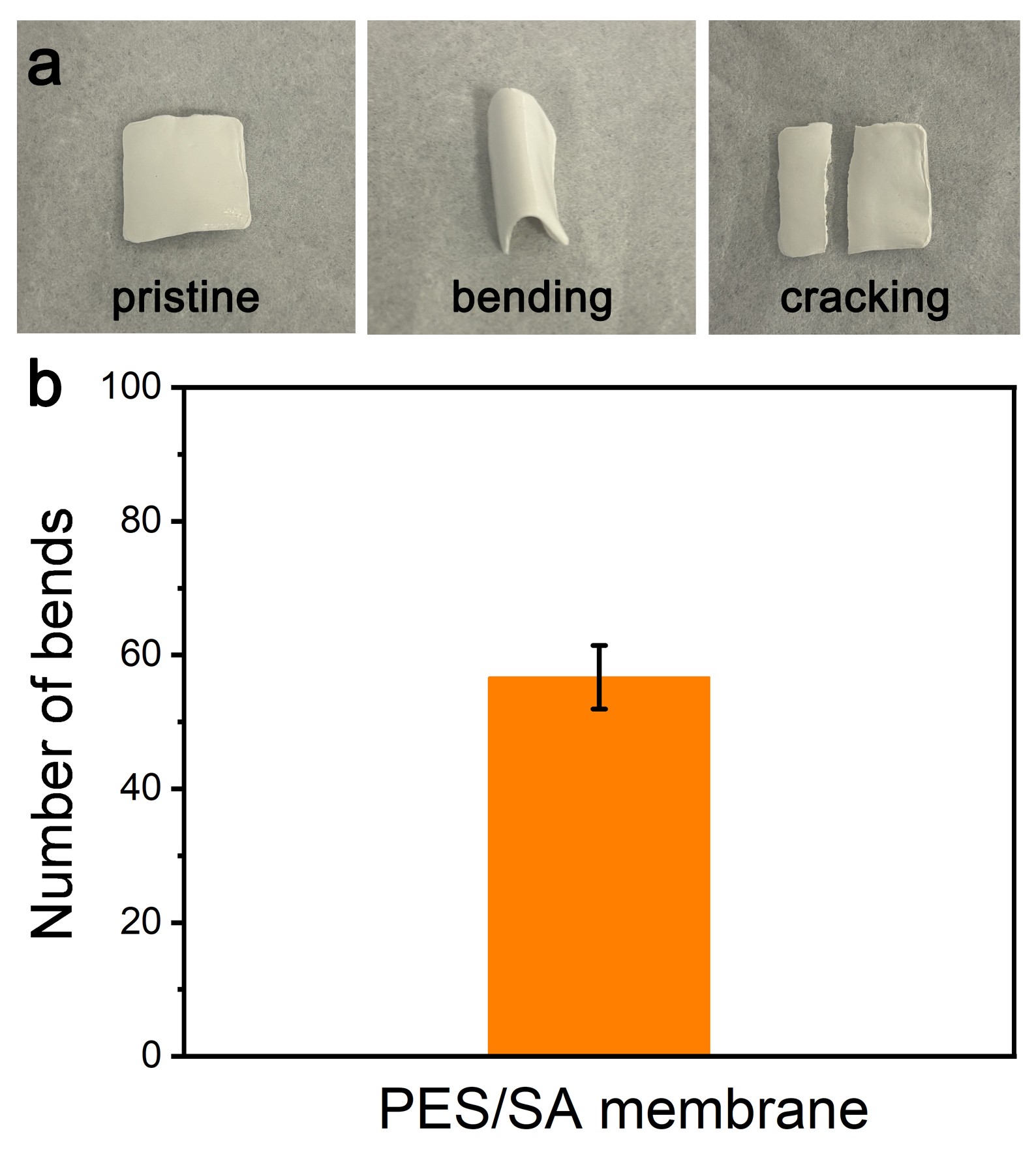


Fig. S7. (a) Membrane under bending test. (b) Average number of bends when membrane is cracked among ten samples. The membrane is 2 cm×2 cm.


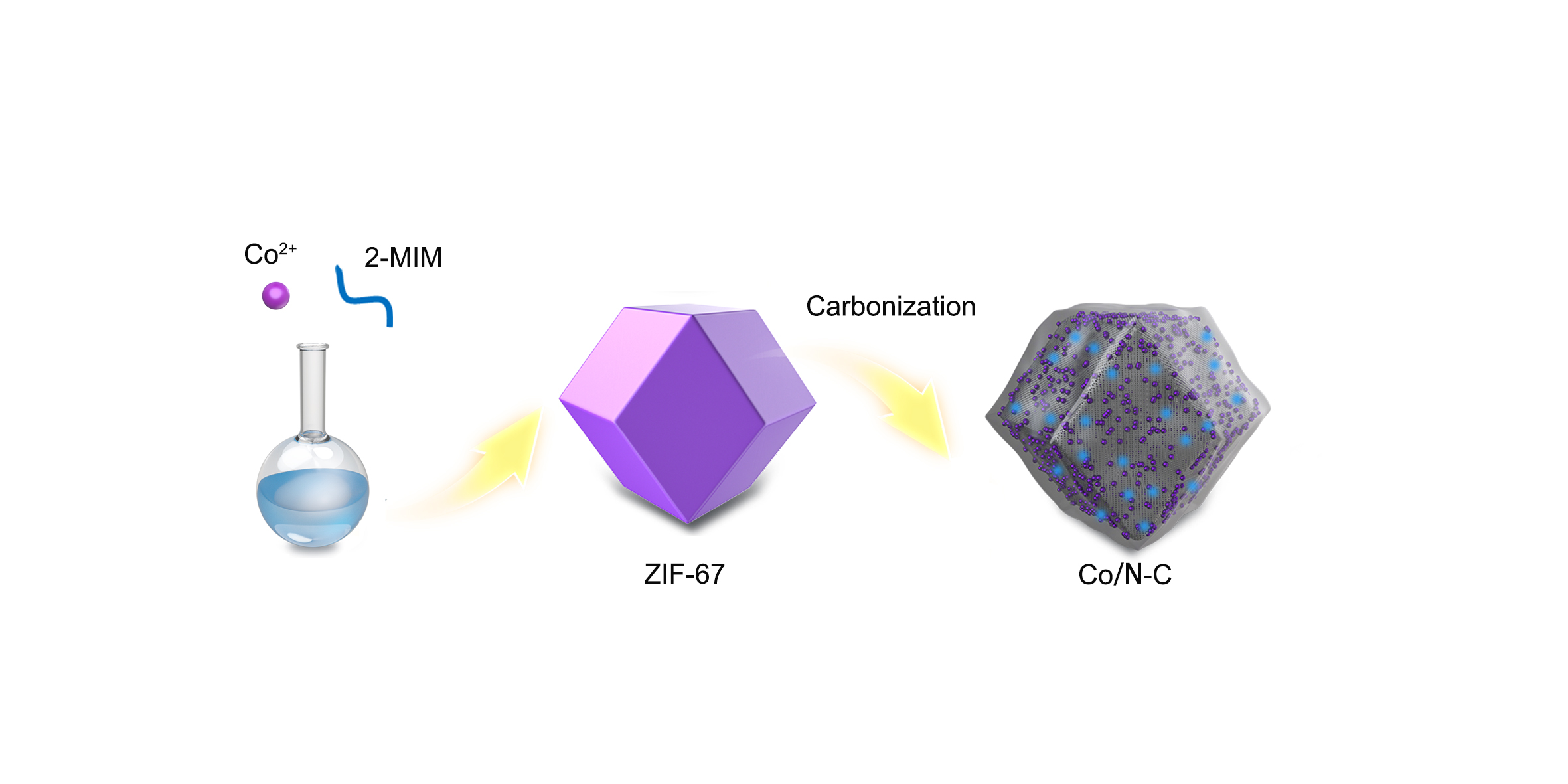


Fig. S8. Synthetic diagram of Co/N-C.


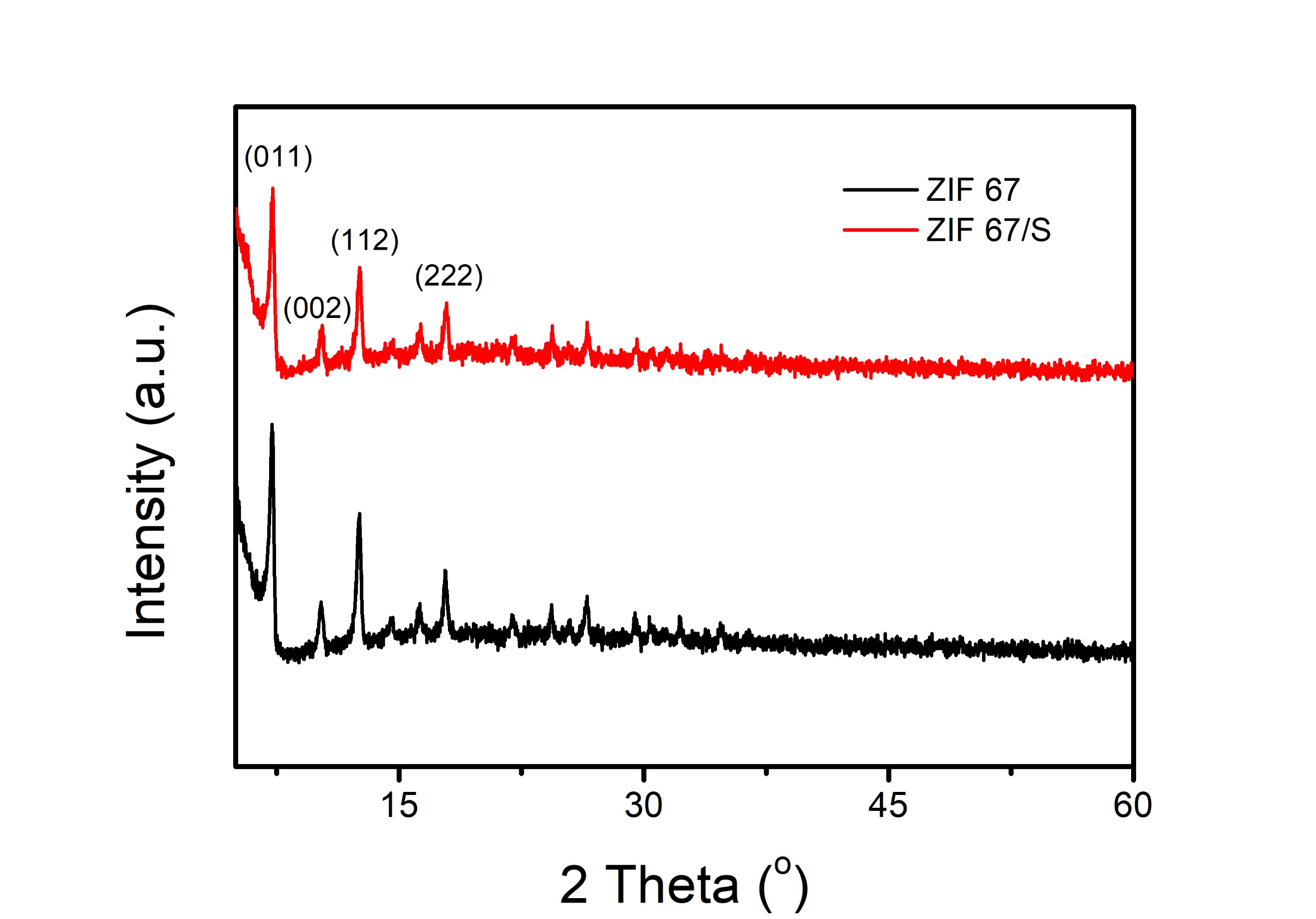


Fig. S9. XRD patterns of ZIF-67 and ZIF-67/S.


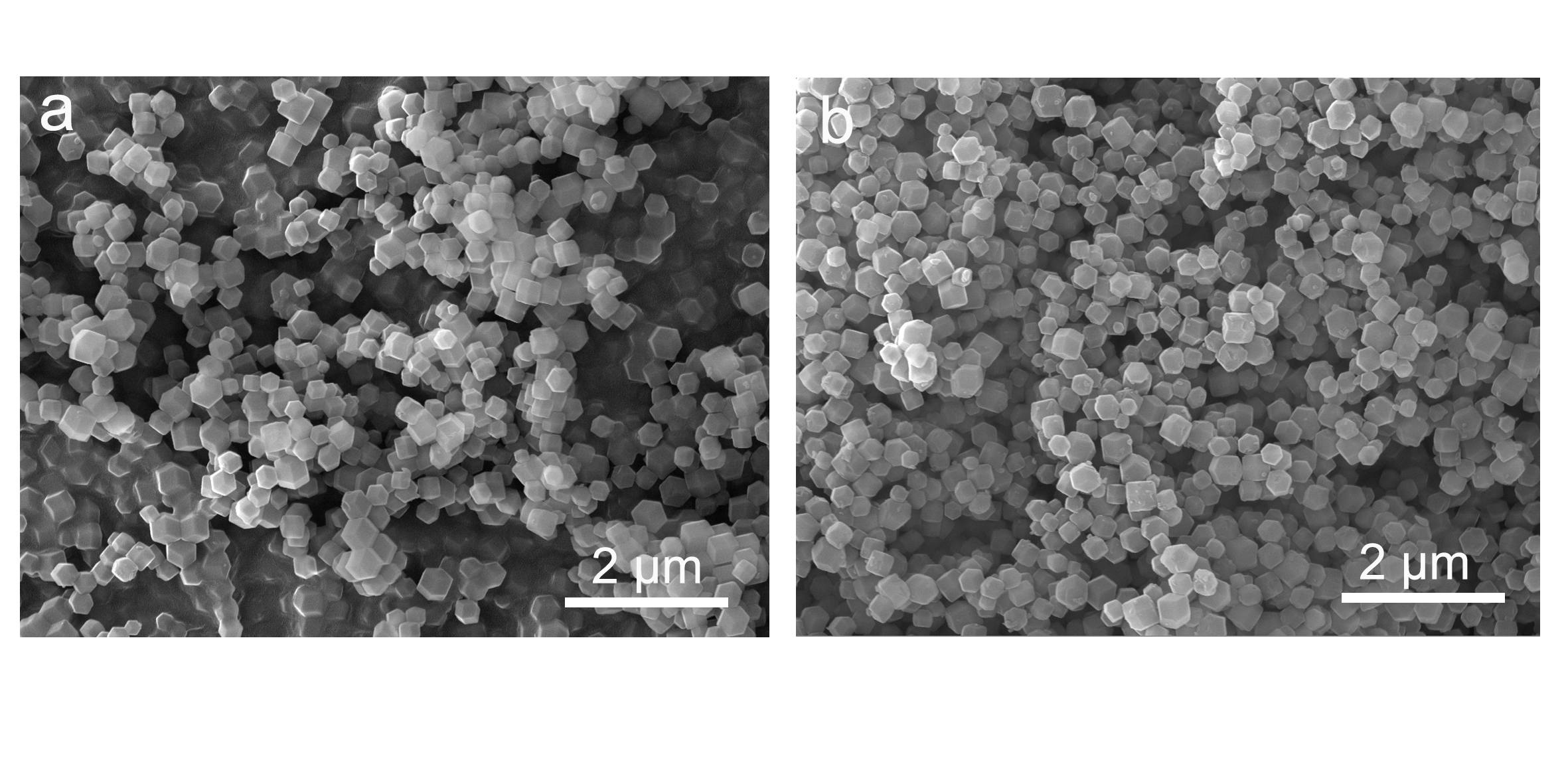


Fig. S10. SEM images of (a) ZIF-67 and (b) ZIF-67/S nanoparticles.


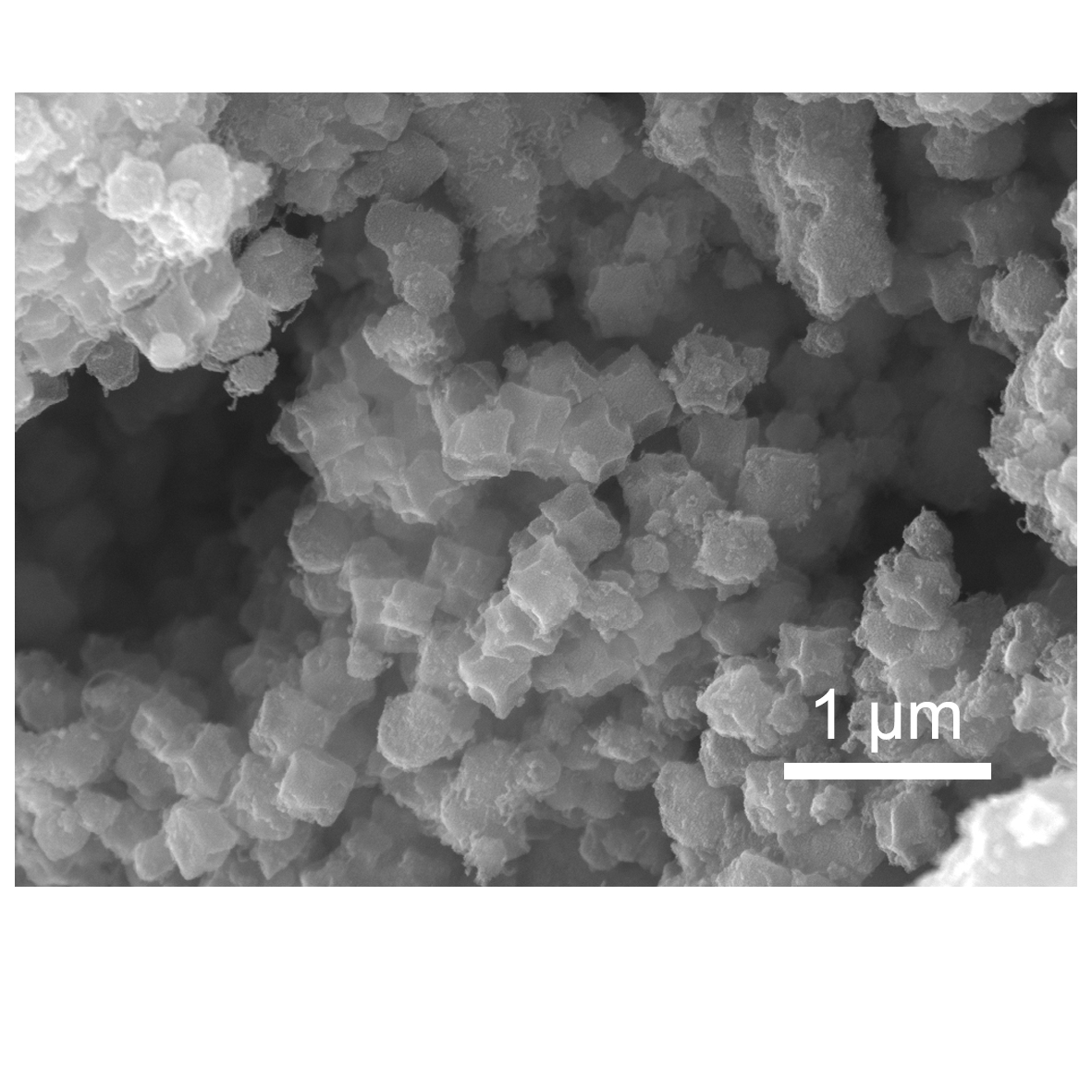


Fig. S11. SEM images of Co/N-C.


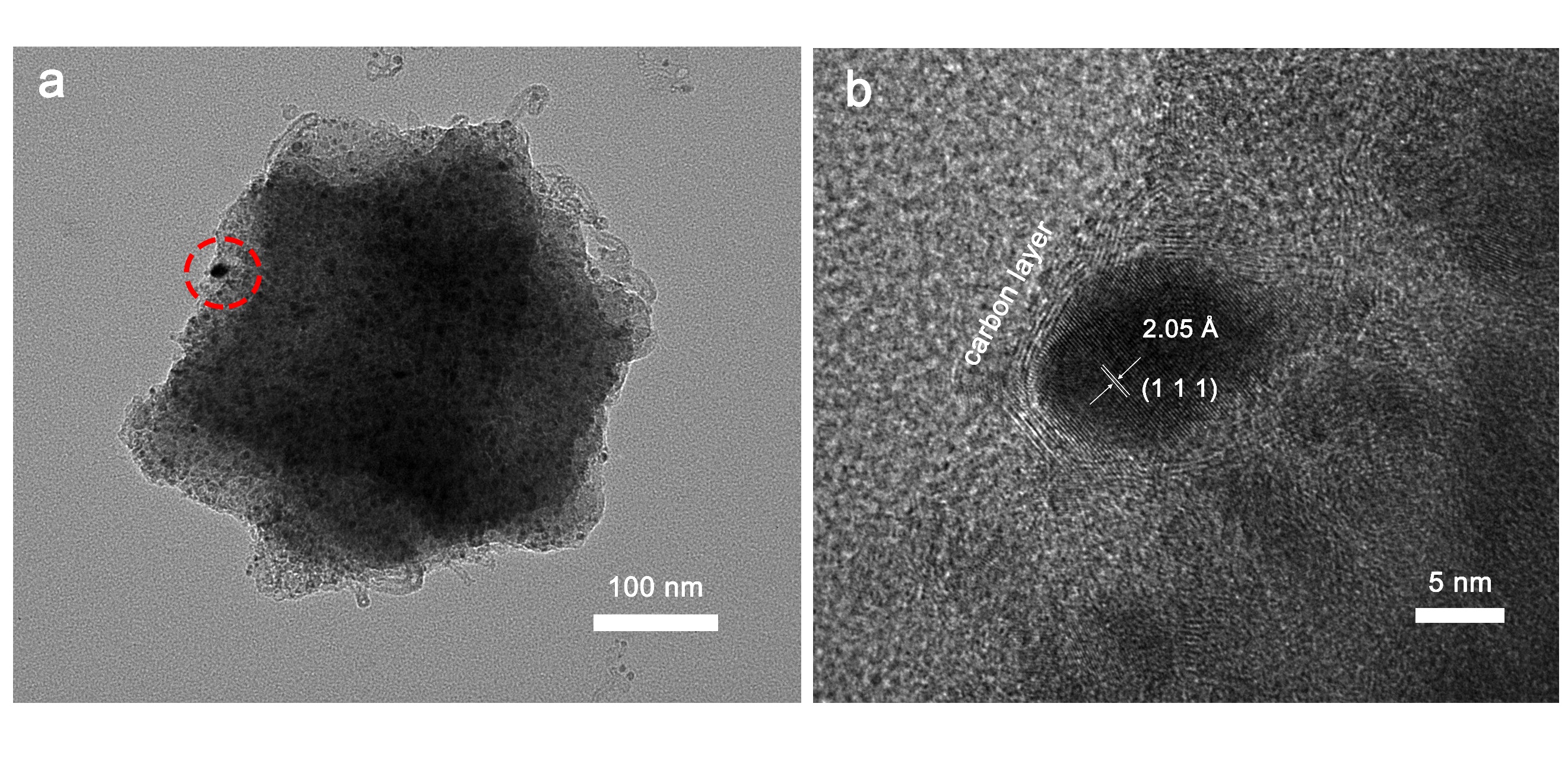


Fig. S12. (a) TEM and (b) HRTEM images of Co/N-C.


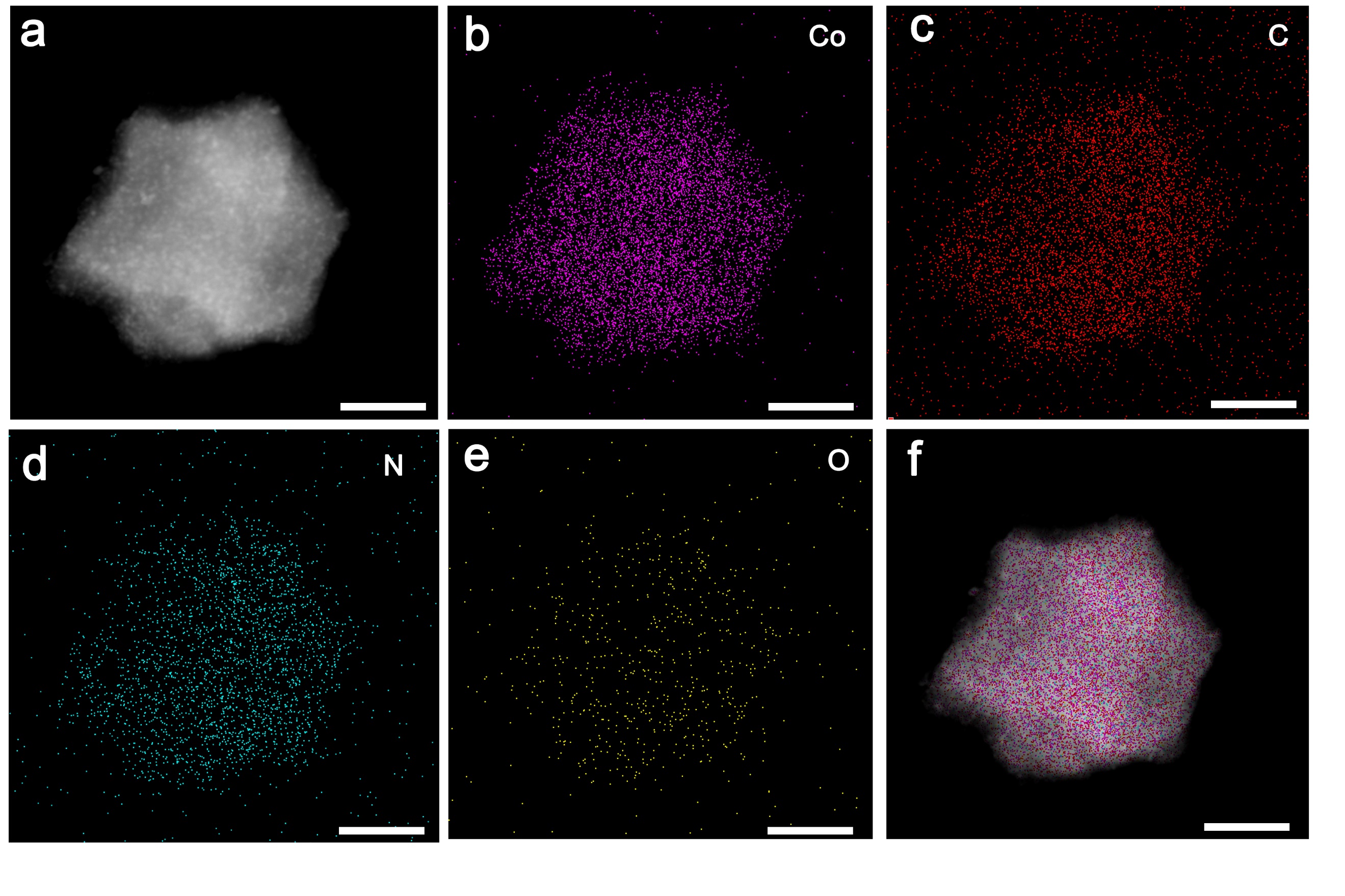


Fig. S13. (a) HAADF image and corresponding EDX mapping image of Co/N-C for element of (b) Co, (c) C, (d) N, (e) O and (f) mixed image. Scale bar is 100 nm.


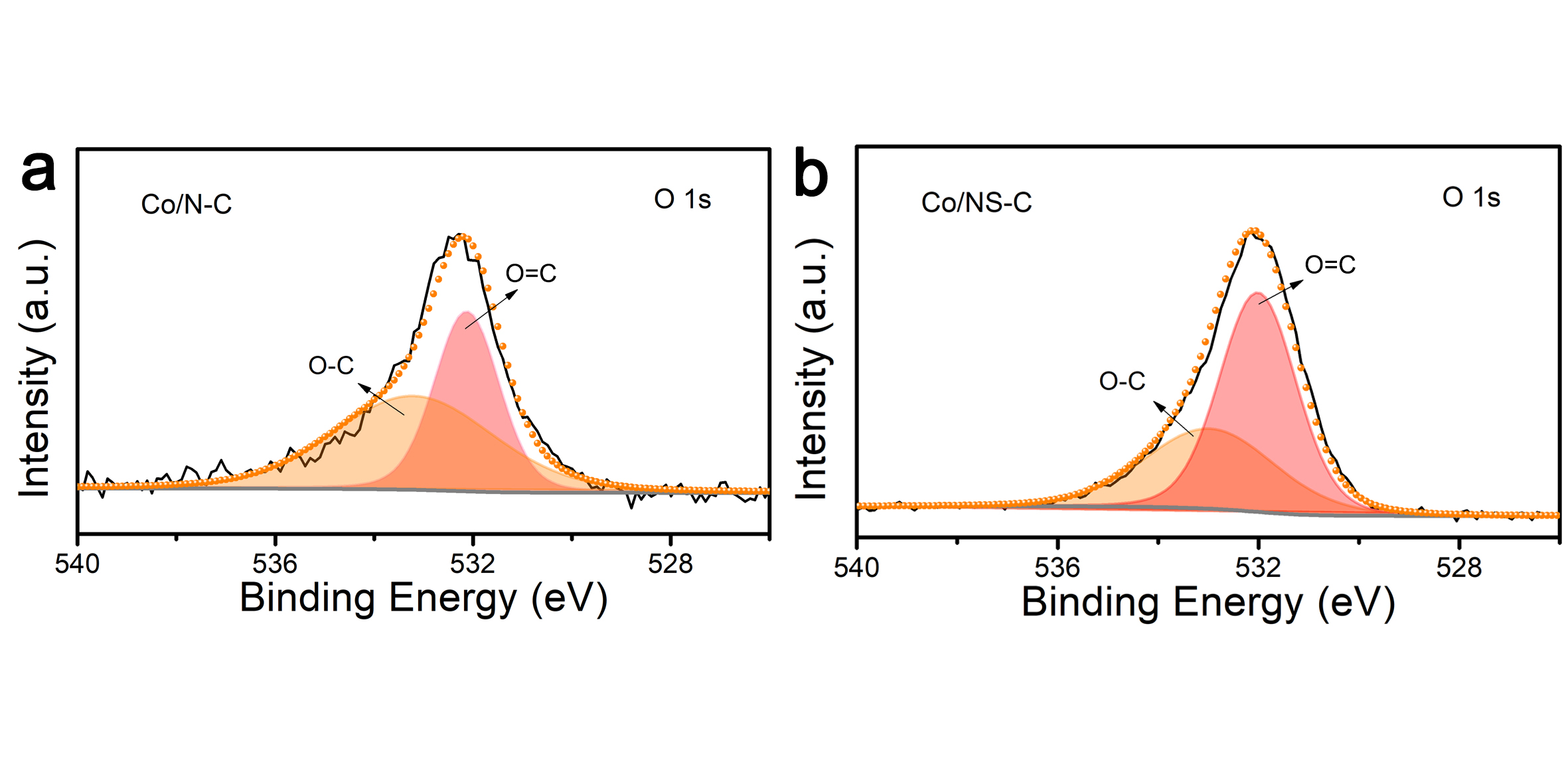


Fig. S14. XPS spectra of O 1s for (a) Co/N-C and (b) Co/NS-C.


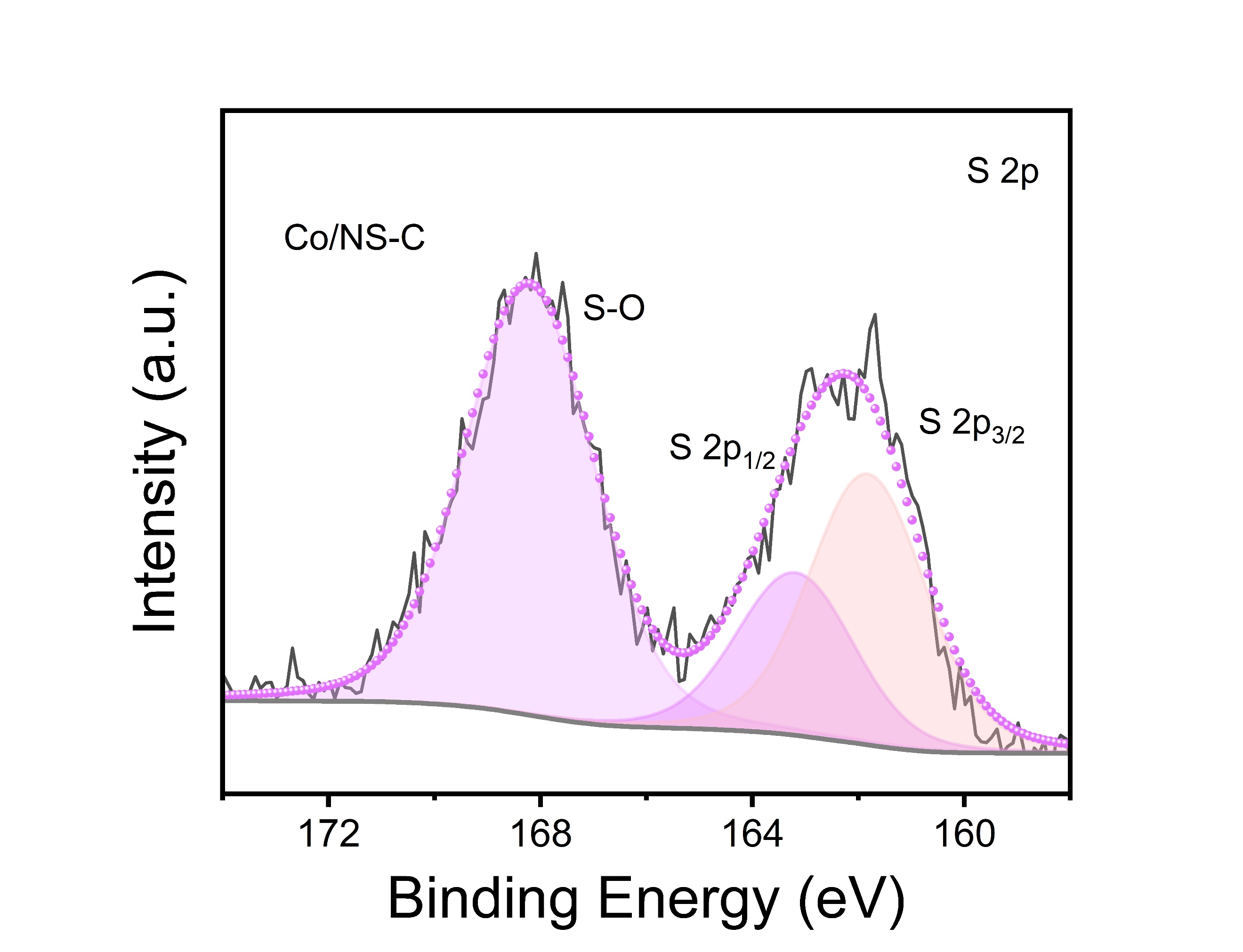


Fig. S15. XPS spectra of S 2p for Co/NS-C.


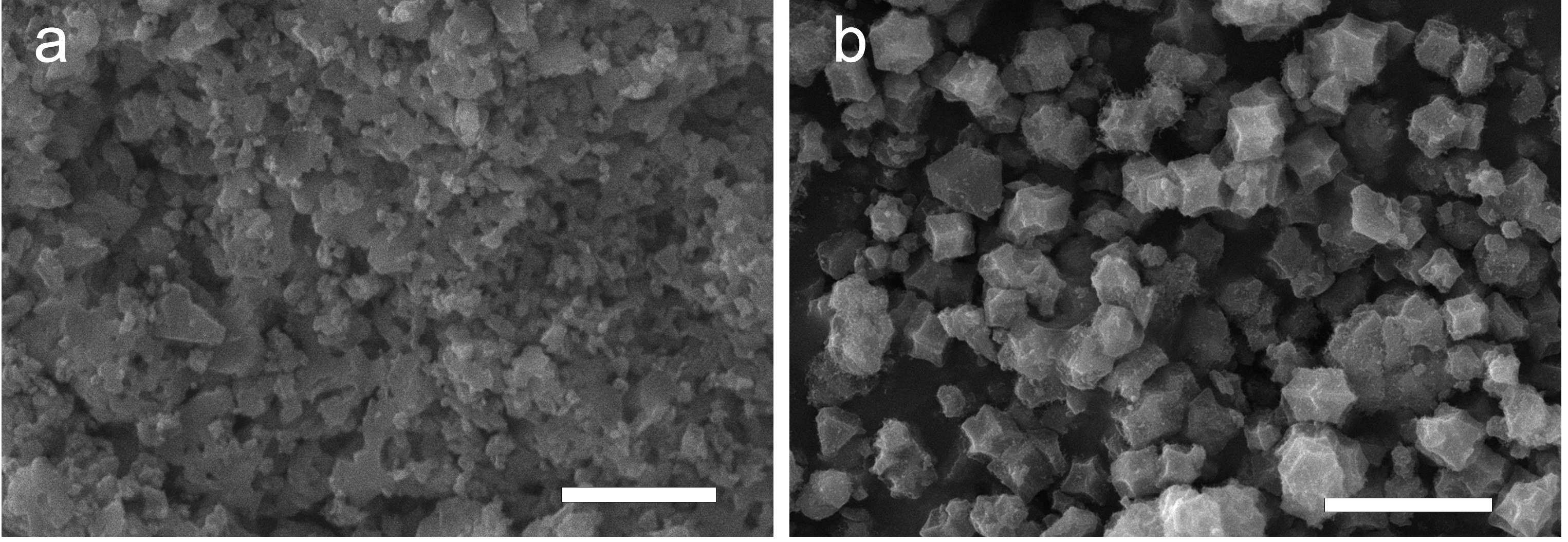


Fig. S16. SEM images of (a) NS-C and (b) Co(mix)/NS-C. Scale bar is 2 μm.


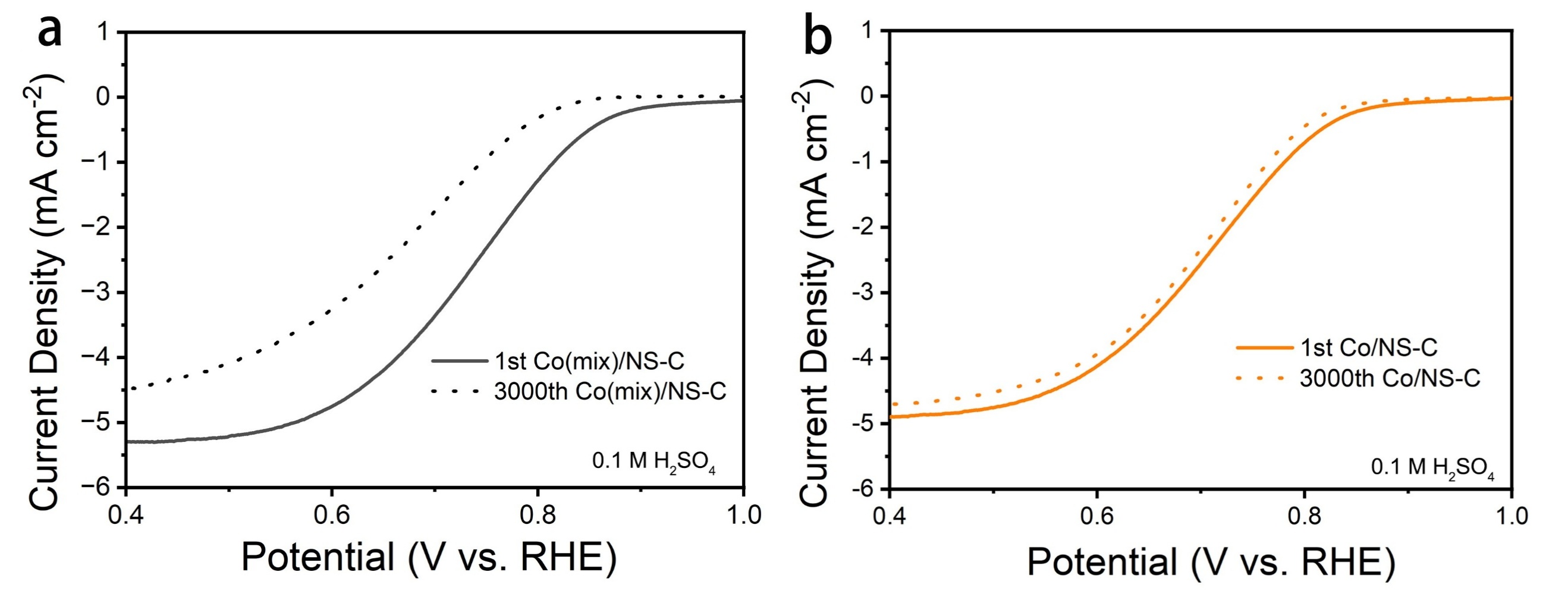


Fig. S17. LSV curves of (a) Co(mix)/NS-C and (b) Co/NS-C before and after 3000 cycles test in 0.1 M H_2_SO_4_.


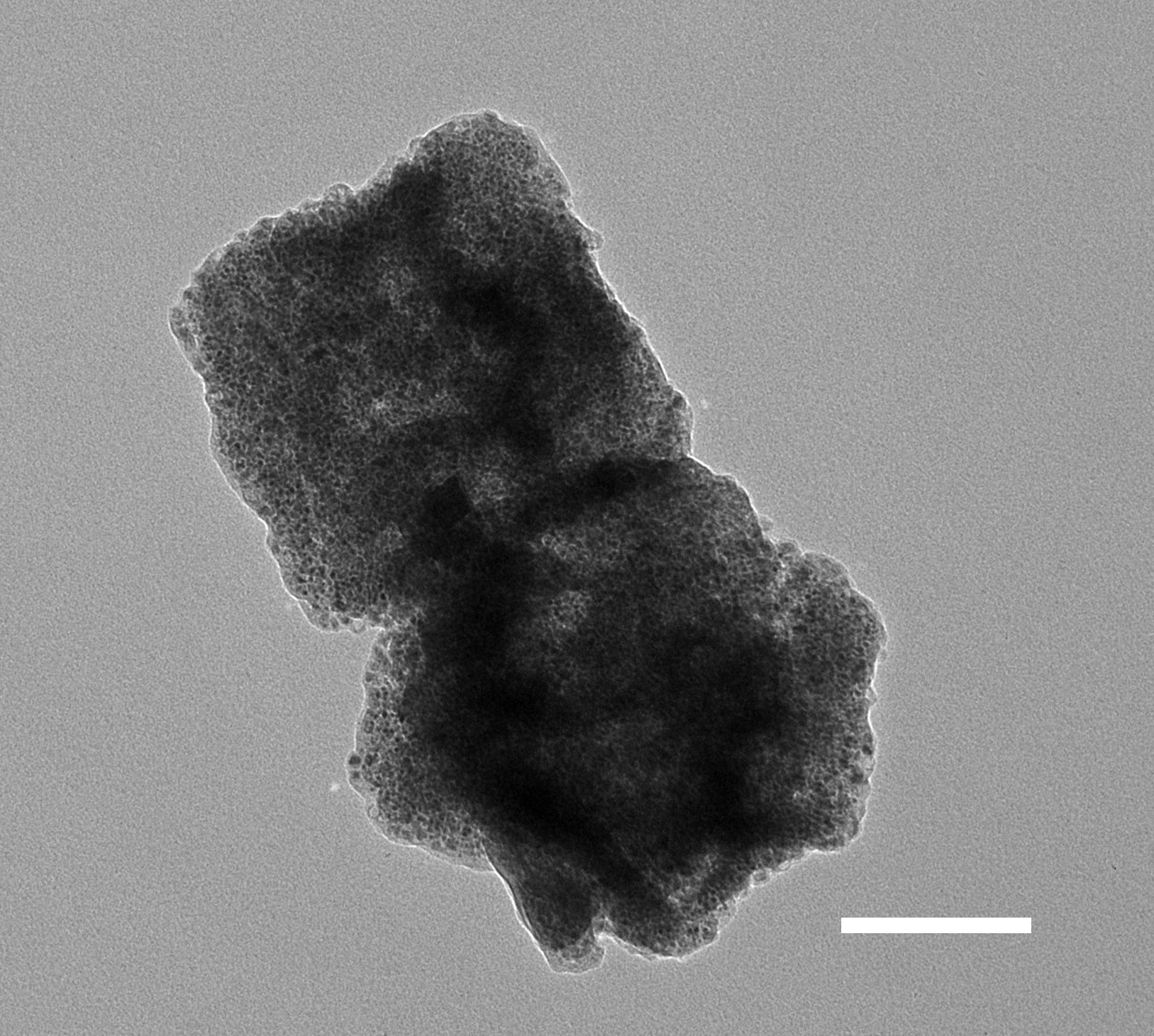


Fig. S18. TEM image of Co/NS-C after 3000 cycles test. Scale bar is 200 nm.


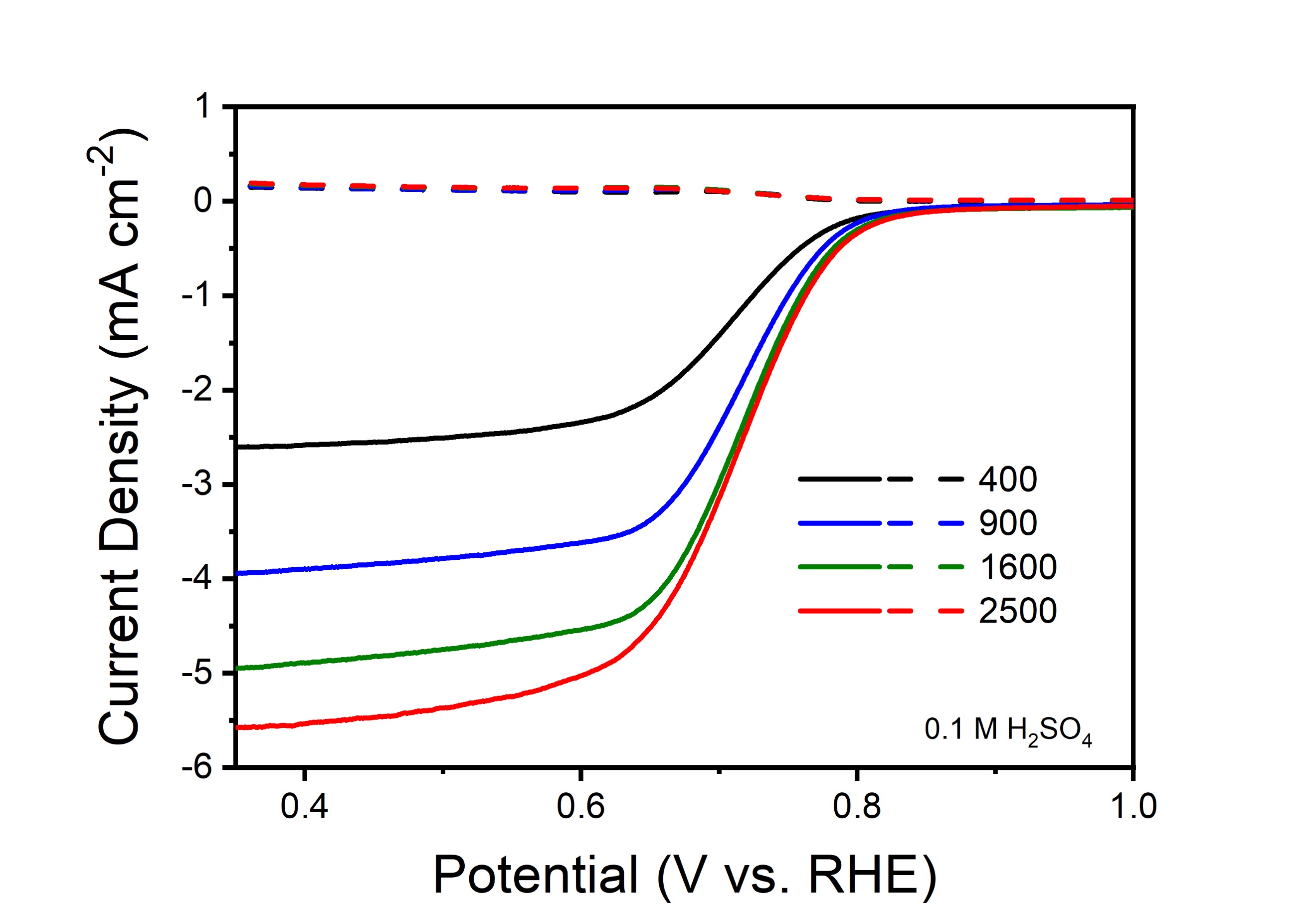


Fig. S19. LSV curves of Co/NS-C under different spin speed of RRDE in O_2_ saturated 0.1 M H_2_SO_4_.


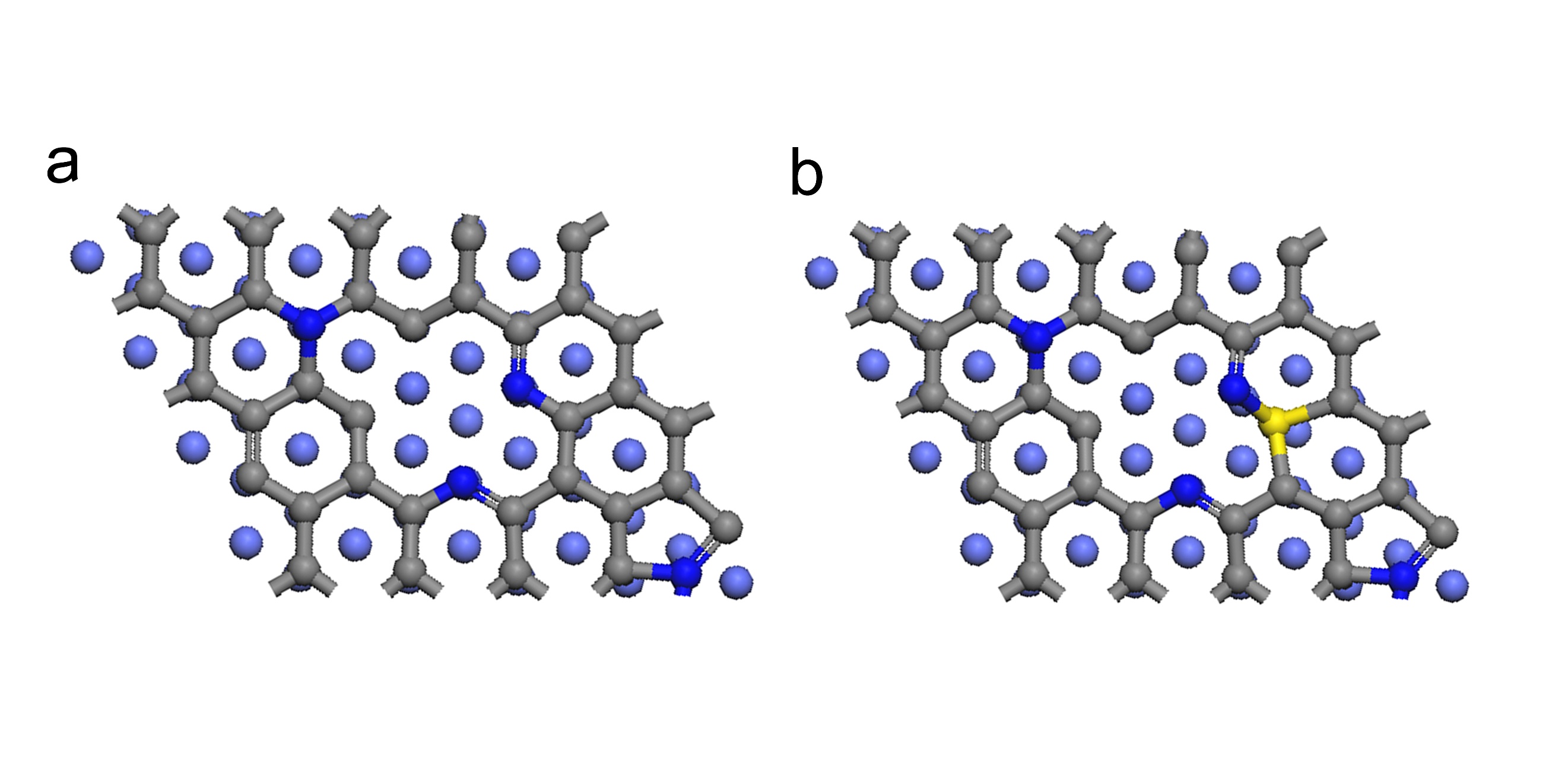


Fig. S20. The structural models of (a) Co/N-C and (b) Co/NS-C from top view. The indigo, blue, yellow and gray balls represent cobalt, nitrogen, sulfur and carbon, respectively.


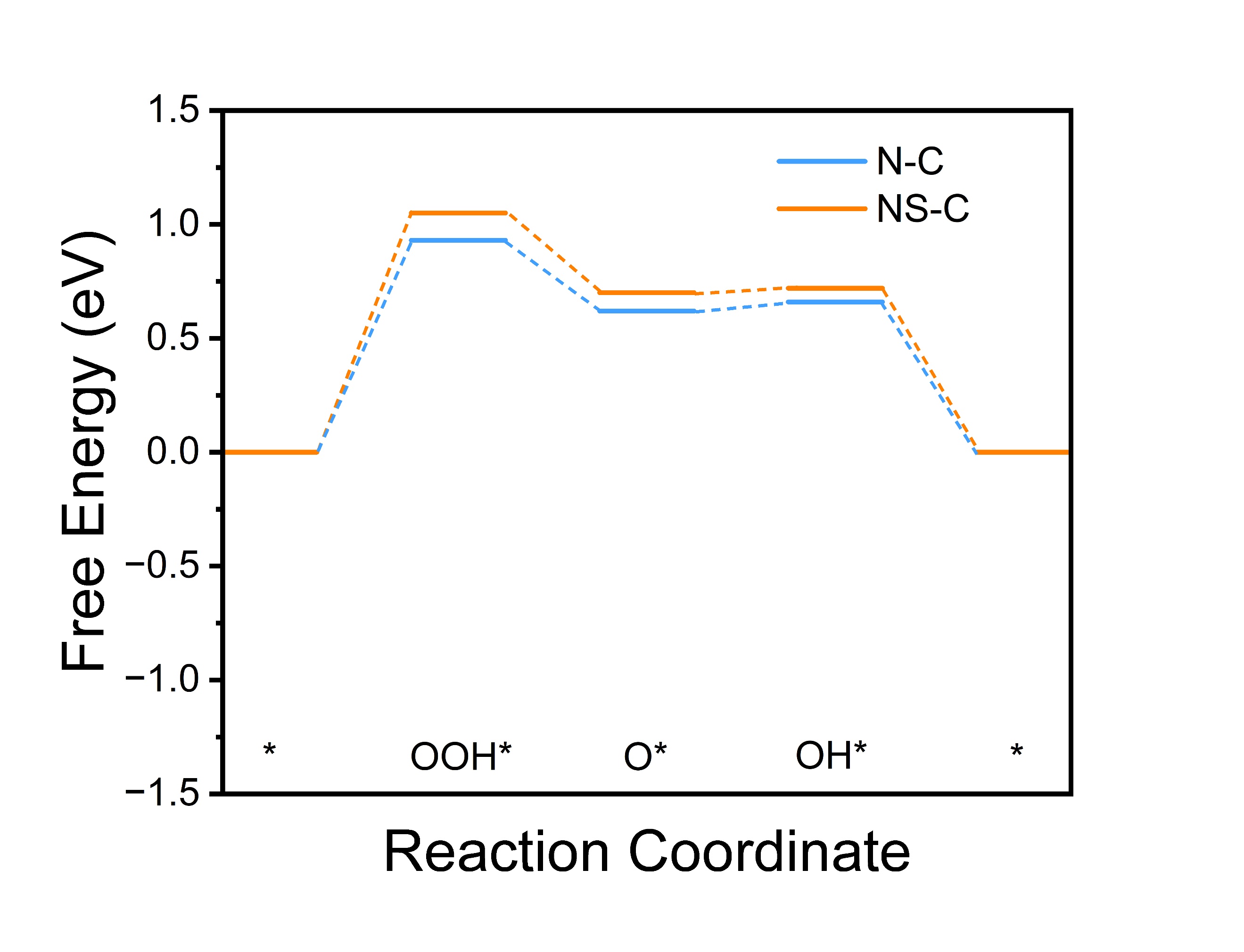


Fig. S21. ORR free energy diagrams of N-C and NS-C.


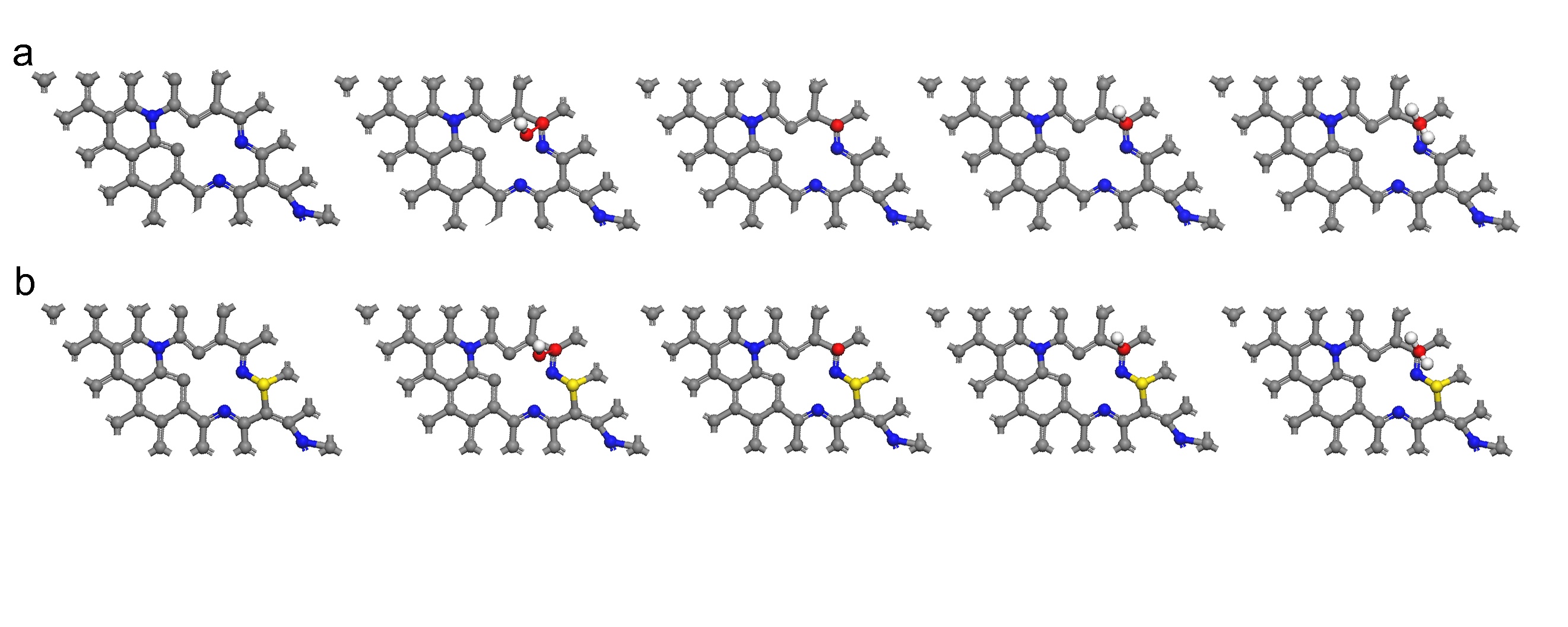


Fig. S22. Schematic diagrams of optimized intermediate models for ORR process of (a) N-C and (b) NS-C. The indigo, blue, gray, yellow, red and white balls represent cobalt, nitrogen, carbon, sulfur, oxygen and hydrogen, respectively.


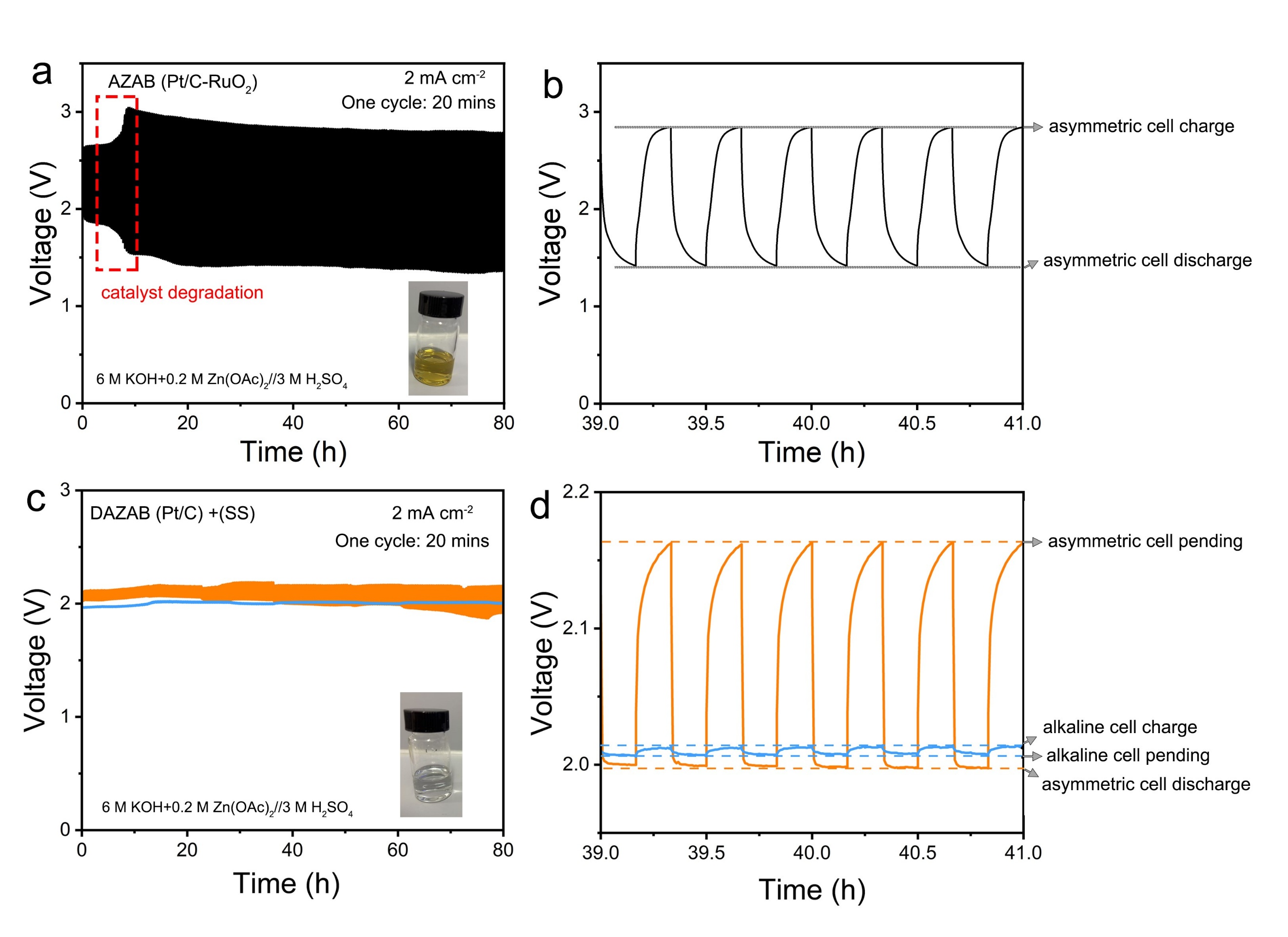


Fig. S23. Battery cyclic performance based on Pt/C for ORR. (a) AZAB performance with (b) Enlarged cycles around 40 h. (c) DAZAB performance with (d) Enlarged cycles around 40 h. Inserted figures show the electrolytes in acidic cell after 80 h.


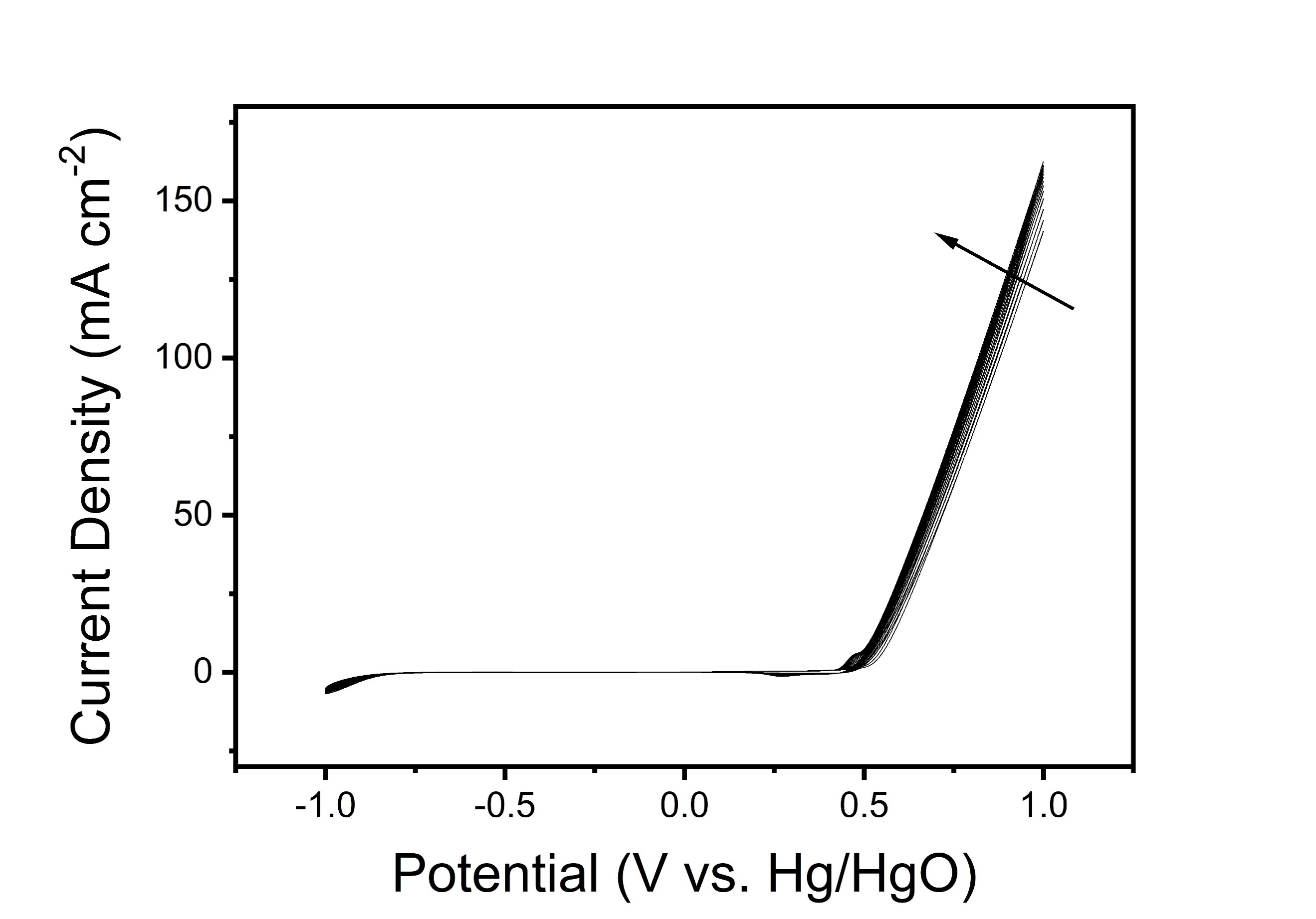


Fig. S24. CV curves of activation of SS mesh for 10 cycles in 1 M KOH.


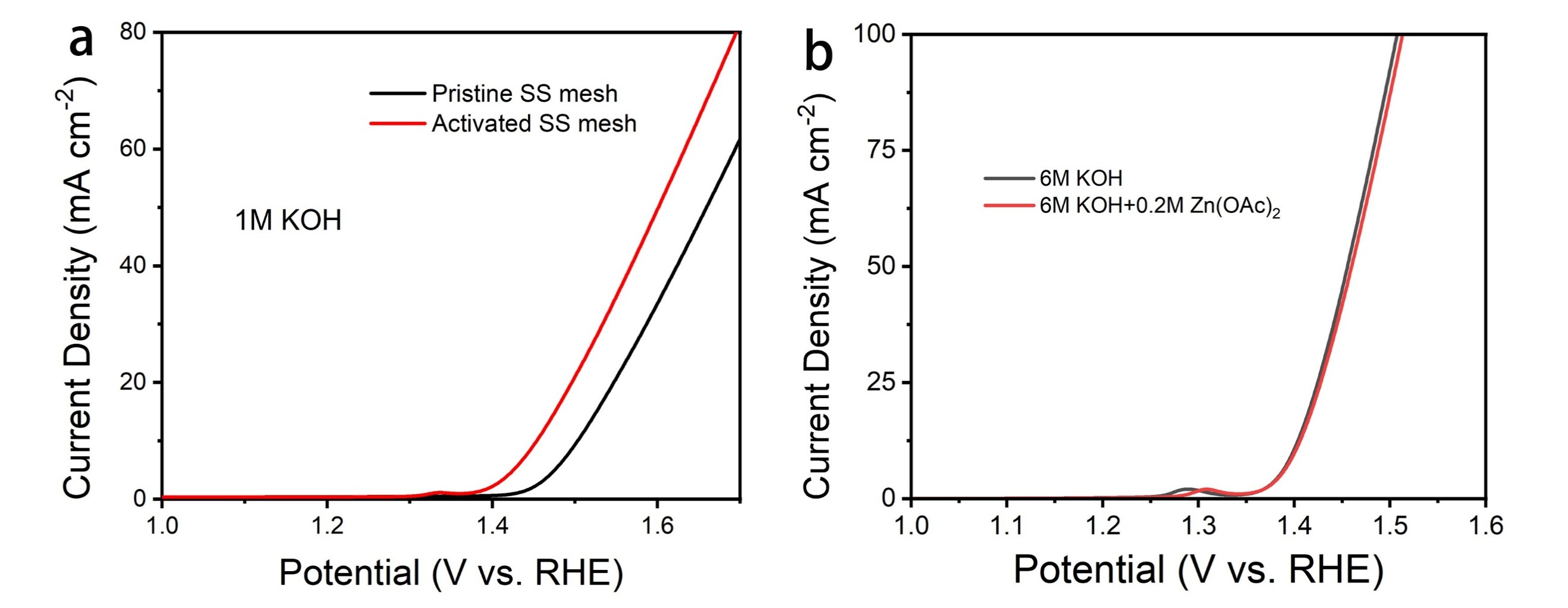


Fig. S25. LSV curves without iR compensation of (a) pristine SS and activated SS in 1 M KOH and (b) activated SS in 6 M KOH and 6 M KOH with 0.2 M Zn(OAc)_2_.


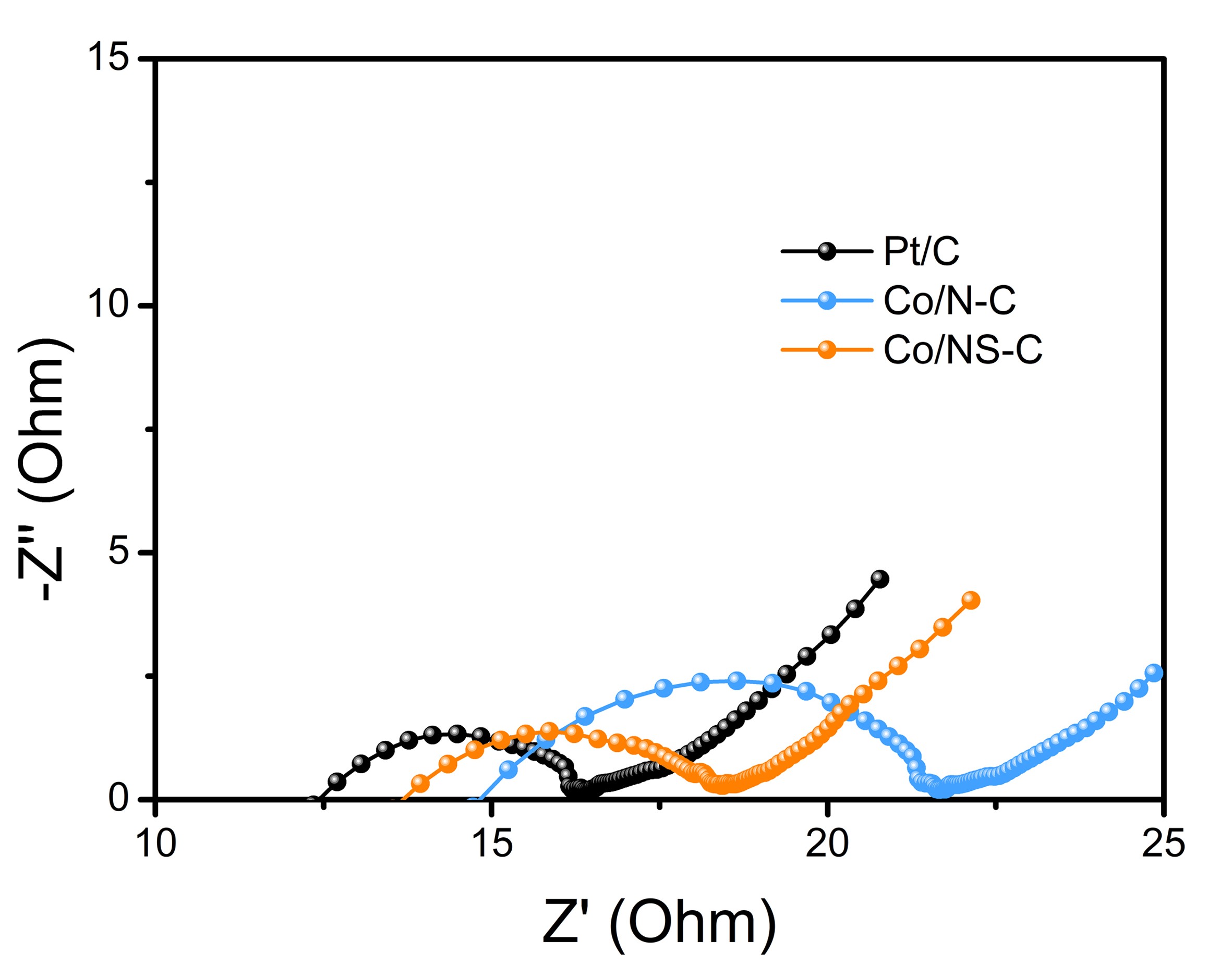


Fig. S26. Nyquist plot curves of DAZAB based on different air electrodes.


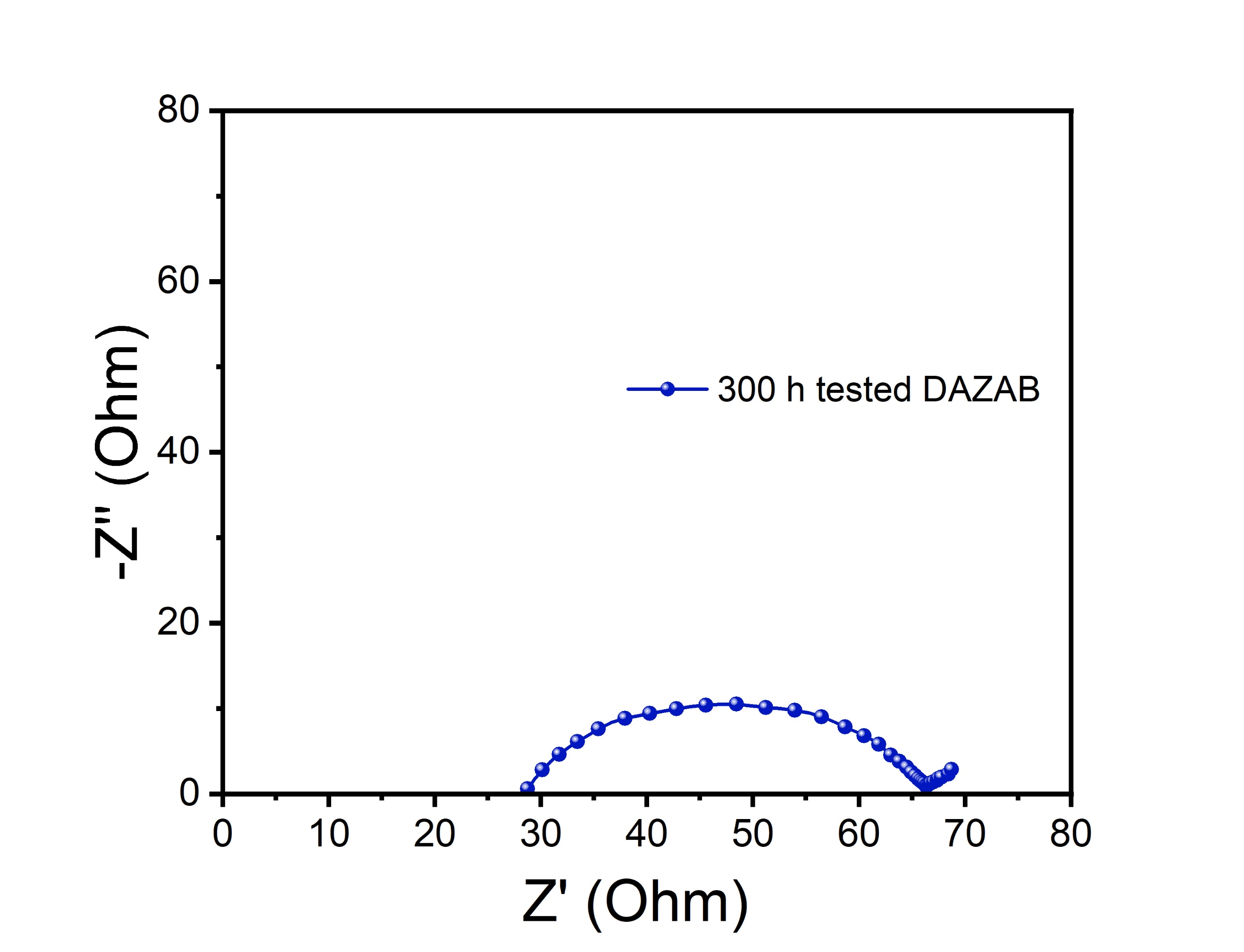


Fig. S27. Nyquist plot curve of DAZAB based on Co/NS-C after 300 h test.


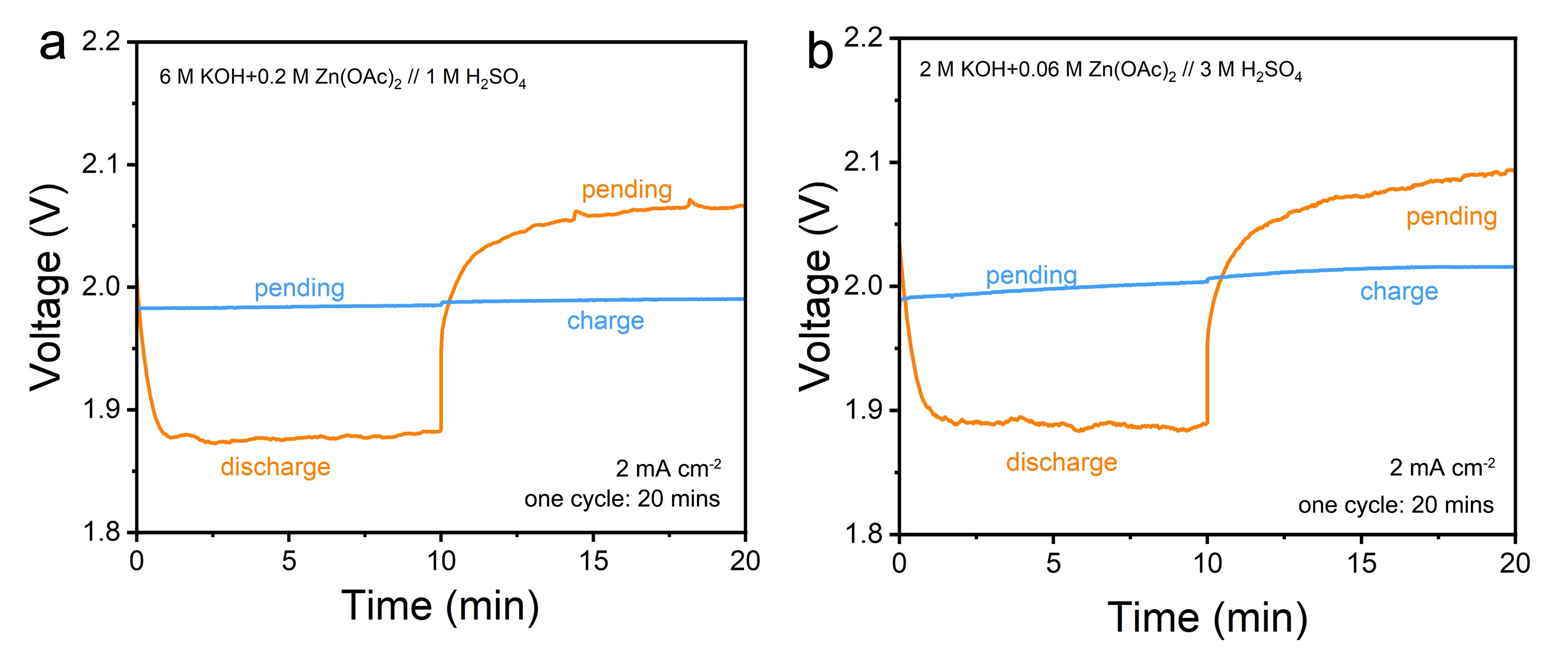


Fig. S28. Battery performance with refresh of two combinations of electrolytes after 300 h test.


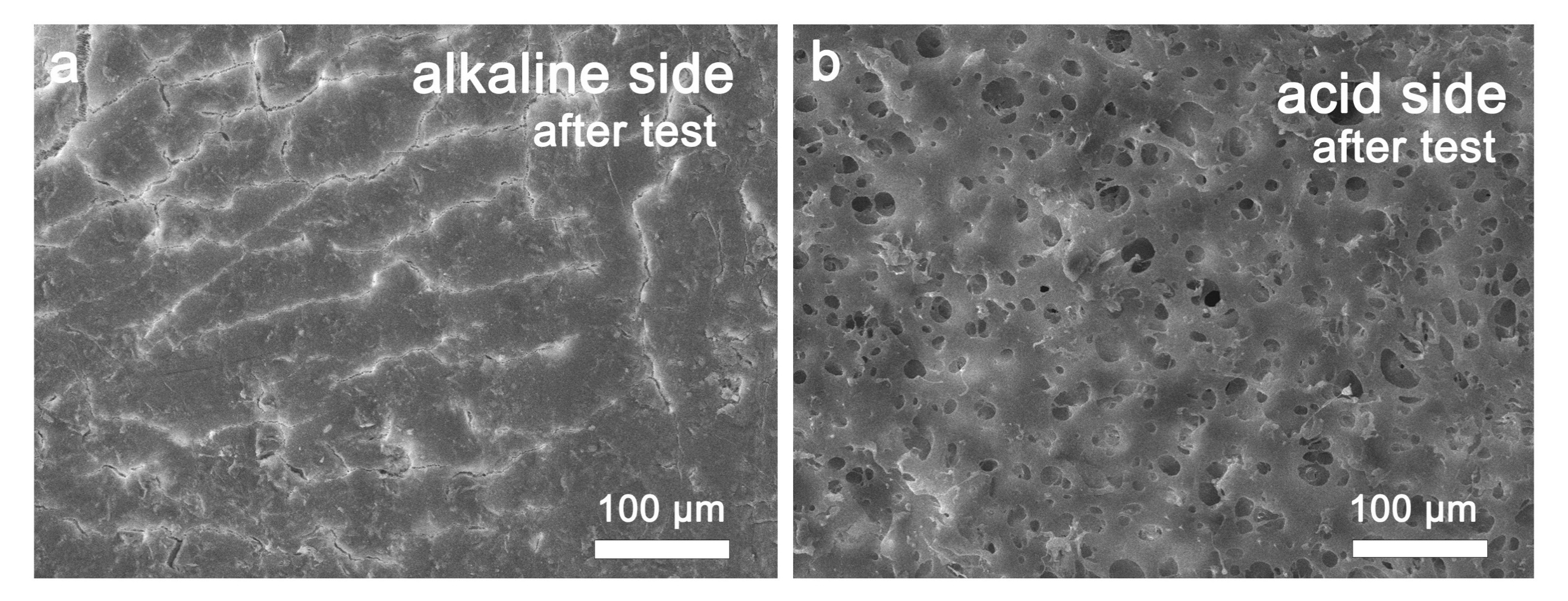


Fig. S29. SEM image of PES/SA membrane (a) alkaline side and (b) acid side after DAZAB test for 300 h.


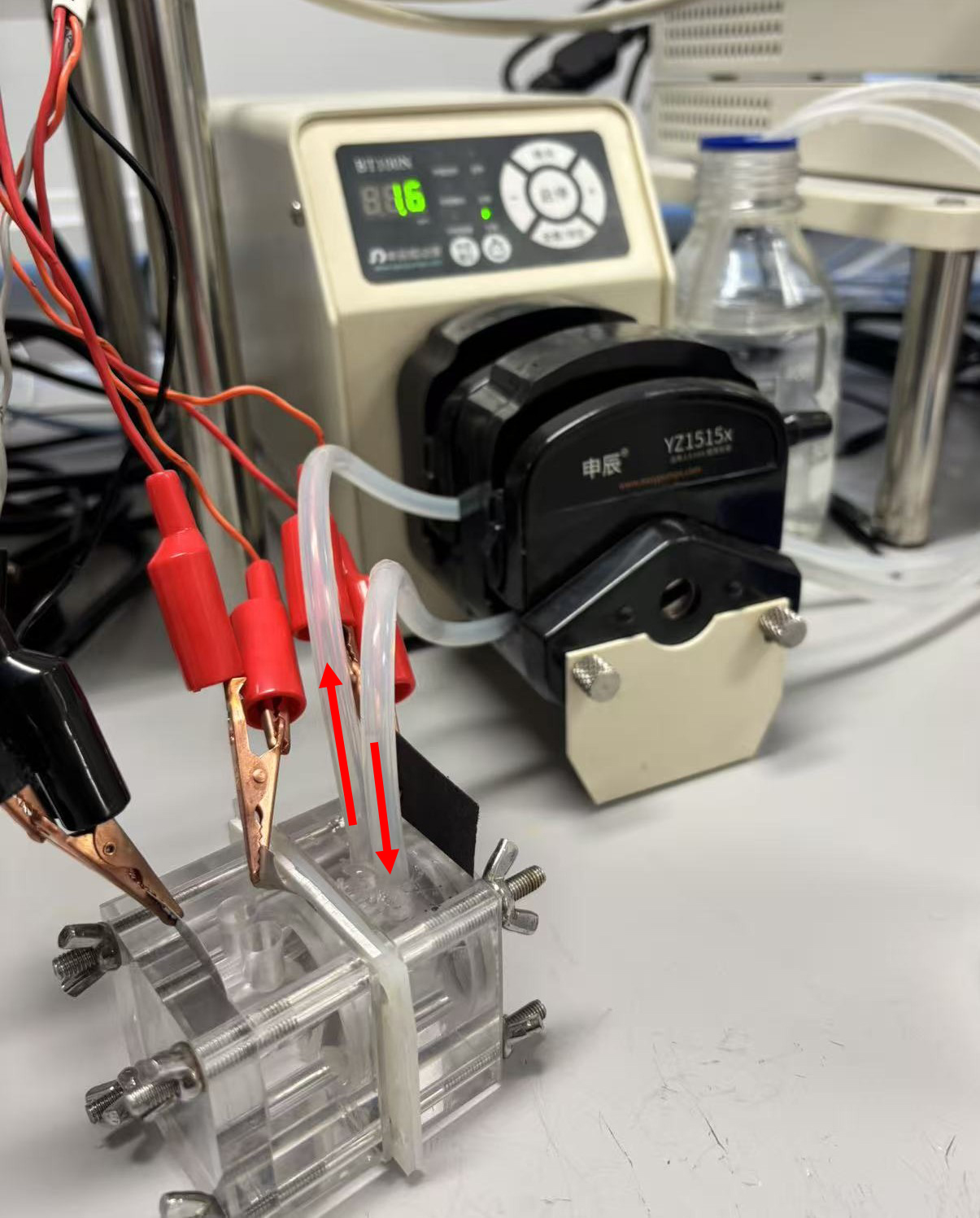


Fig. S30. Illustration of electrolyte exchange.


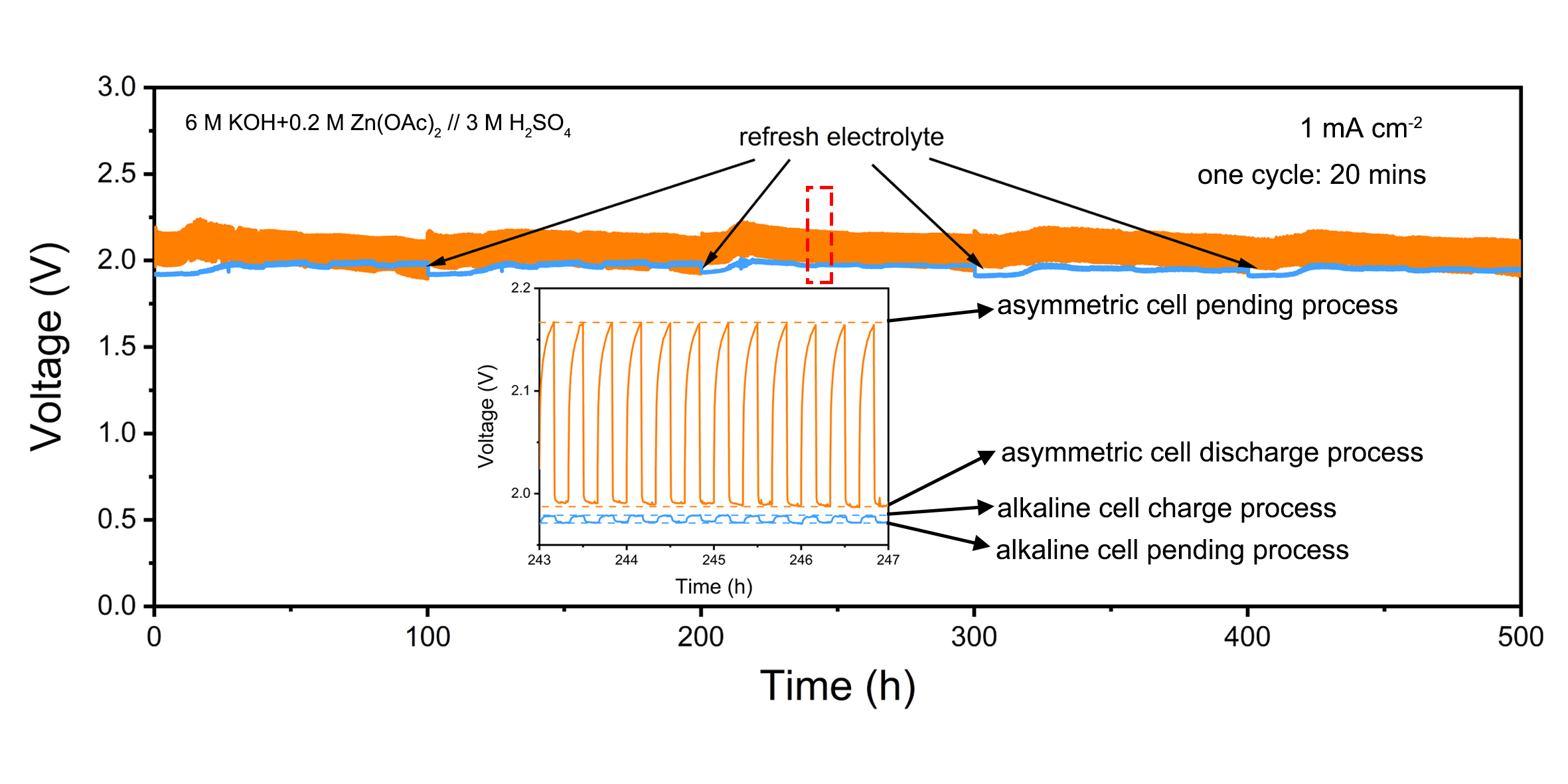


Fig. S31. Cycles performance of DAZAB with Co/NS-C as air electrode under current density of 1 mA cm^-2^.

Table S4. pH values (H^+^) and amount of SO_4_^2-^ for crossover of PES/SA membrane.

| Membrane | pH | | | SO_4_^2-^ (ppm) | | |
| --- | --- | --- | --- | --- | --- | --- |
|  | 0 mA cm^-2^ | 1 mA cm^-2^ | 2 mA cm^-2^ | 0 mA cm^-2^ | 1 mA cm^-2^ | 2 mA cm^-2^ |
| 1 | 2.068 | 1.521 | 1.473 | 277 | 168 | 103 |
| 2 | 2.113 | 1.534 | 1.487 | 256 | 149 | 95 |
| 3 | 2.095 | 1.525 | 1.478 | 261 | 151 | 99 |
| 4 | 2.056 | 1.507 | 1.461 | 293 | 182 | 115 |
| 5 | 2.062 | 1.524 | 1.477 | 284 | 178 | 108 |

Table S5. pH values (OH^-^) and amount of K^+^ and Zn^2+^ for crossover of PES/SA membrane.

| Membrane | pH | | | Zn^2+^ (ppm) | | | K^+^ (ppm) | | |
| --- | --- | --- | --- | --- | --- | --- | --- | --- | --- |
|  | 0 mA cm^-2^ | 1 mA cm^-2^ | 2 mA cm^-2^ | 0 mA cm^-2^ | 1 mA cm^-2^ | 2 mA cm^-2^ | 0 mA cm^-2^ | 1 mA cm^-2^ | 2 mA cm^-2^ |
| 1 | 12.358 | 12.976 | 13.079 | 31.8 | 21.9 | 11.7 | 591 | 457 | 342 |
| 2 | 12.321 | 12.959 | 13.067 | 28.2 | 20.4 | 10.5 | 552 | 432 | 322 |
| 3 | 12.317 | 12.955 | 13.060 | 29.4 | 20.1 | 11.1 | 537 | 425 | 318 |
| 4 | 12.404 | 12.991 | 13.091 | 33.6 | 22.8 | 13.5 | 633 | 482 | 368 |
| 5 | 12.328 | 12.962 | 13.069 | 33.3 | 23.4 | 12.6 | 615 | 468 | 357 |

Table S6. Parameters of DAZAB under different operation time.

| Time (h) | Acid cell  (pH) | Alkaline cell  (pH) | pH difference | Discharge Voltage (V) | Charge Voltage (V) | Modified Round-trip Efficiency (%) |
| --- | --- | --- | --- | --- | --- | --- |
| 0 | -0.665 | 14.771 | 15.436 | 1.84 | 1.93 | 48.15 |
| 20 | -0.597 | 14.72 | 15.317 | 1.95 | 2.03 | 51.54 |
| 40 | -0.533 | 14.676 | 15.209 | 1.95 | 2.01 | 52.37 |
| 60 | -0.458 | 14.629 | 15.087 | 1.93 | 2.02 | 51.48 |
| 80 | -0.369 | 14.576 | 14.945 | 1.90 | 2.03 | 50.16 |
| 100 | -0.279 | 14.524 | 14.803 | 1.88 | 2.05 | 49.10 |
| 120 | -0.193 | 14.481 | 14.673 | 1.85 | 2.06 | 47.78 |
| 140 | -0.121 | 14.449 | 14.57 | 1.80 | 2.07 | 45.43 |
| 160 | -0.053 | 14.420 | 14.473 | 1.73 | 2.08 | 42.12 |
| 180 | 0.018 | 14.391 | 14.373 | 1.65 | 2.08 | 38.56 |
| 200 | 0.092 | 14.362 | 14.27 | 1.61 | 2.09 | 36.75 |
| 300 | 0.893 | 14.196 | 13.303 | 1.34 | 2.14 | 25.94 |

Table S7. Parameters of DAZAB with new electrolytes in efficient range of pH difference

| DAZAB | Alkaline cell | Acid cell | pH difference | Discharge voltage (V) | Charge voltage (V) | Modified Round-trip efficiency |
| --- | --- | --- | --- | --- | --- | --- |
| 1 | 2 M KOH+0.06 M Zn(OAc)_2_  pH:14.257 | 3 M H_2_SO_4_  pH:-0.689 | 14.946 | 1.89 | 2.01 | 50.16% |
| 2 | 6 M KOH+0.2 M Zn(OAc)_2_  pH: 14.756 | 1 M H_2_SO_4_  pH: -0.118 | 14.874 | 1.86 | 1.98 | 49.62% |

Table S8. Parameters of Zn^2+^ amount in DAZAB before and after test.

| DAZAB | Alkaline cell (Zn^2+^) | Acid cell (Zn^2+^) |
| --- | --- | --- |
| pristine | 8 mL, 0.2027 M | 8 mL, 0 |
| 300 h test | 7.08 mL, 0.0305 M | 7.08 mL, 0.0018 M |

Table S9. Parameters of battery configuration and measurements.

| Battery configuration | | |
| --- | --- | --- |
| Discharge cell | ORR electrode | Zinc electrode |
|  | Co/NS-C | Zn foil |
| Electrode area | 1 cm^2^ | 1 cm^2^ |
| Catalyst loading | 1.2 mg cm^-2^ | N/A |
| Current collector | Carbon paper | N/A |
| Binder | Nafion 117 | N/A |
| Electrolyte | 3 M H_2_SO_4_ | 6 M KOH +0.2 M Zn(OAc)_2_ |
| Electrolyte amount | 8 mL | 8 mL |
| Charge cell | OER electrode | Zinc electrode |
|  | Activated SS | Zinc foil |
| Electrode area | 1 cm^2^ | 1 cm^2^ |
| Catalyst loading | N/A | N/A |
| Current collector | N/A | N/A |
| Binder | N/A | N/A |
| Electrolyte | 6 M KOH +0.2 M Zn(OAc)_2_ | 6 M KOH +0.2 M Zn(OAc)_2_ |
| Electrolyte amount | 8 mL | 8 mL |
| Battery measurements and calculations | | |
| Battery system | Half-open system | |
| Gas flow control | No | |
| CO_2_ control | No | |
| Testing temperature | Room temperature (25 degrees) | |
| Cyclic current | 2 mA cm^-2^ / 1 mA cm^-2^ | |
| Cyclic time | 20 mins (10 mins for discharge and 10 mins for charge) | |
| Electrolyte refresh (2 mA cm^-2^) | Every 80 h | |
| Electrolyte refresh times | 4 | |
| Zn amount and utilization for each cycle | 6.22 μmol | |
| Number of cycles tested | 1200 cycles | |
| Electrolyte refresh (1 mA cm^-2^) | Every 100 h | |
| Electrolyte refresh times | 4 | |
| Zn amount and utilization for each cycle | 3.11 μmol | |
| Number of cycles tested | 1500 cycles | |
| Membrane | PES/SA membrane | |
| Separator area | 1.13 cm^2^ | |
| The calculation used to determine the round-trip efficiency (%) | | |
| $\frac{{Discharge voltage}_{modified}}{{Charge voltage}_{modified}}= \frac{{Discharge voltage}_{test}-{pH}_{diff}^{discharge}\times0.059}{{Charge voltage}_{test}-{pH}_{diff}^{charge}\times0.059}$  ${pH}_{diff}^{discharge}$ represents the pH difference value of two electrodes in discharge cell.  ${pH}_{diff}^{charge}$ represents the pH difference value of two electrodes in charge cell. | | |
| Average Round-trip efficiency without changing electrolyte (from 0 to 120 h, every 20 h) | 50.08% | |
| Standard Variability | 1.79% | |

Table S10. Detailed performance parameters of latest reported asymmetric zinc-air batteries.

| **Catalyst** | **Alkaline or Neutral Electrolyte** | **Acidic electrolyte** | **Membrane** | **Current density (mA cm^-2^)** | **Test Round-trip efficiency (%)** | **Modified Round-trip efficiency (%)** | **Reference** |
| --- | --- | --- | --- | --- | --- | --- | --- |
| Co/NS-C | 6M KOH+0.2M Zn(OAc)_2_ | 3M H_2_SO_4_ | PES/SA membrane | 2 | / | 50.08 | This work |
| Co/N carbon | 6M KOH+0.2M Zn(OAc)_2_ | 3M H_2_SO_4_+0.2M Zn(OAc)_2_ | ZnSTM membrane | 0.5 | 76.6 | / | ^[8]^ |
| PolyCo@MXene | 6M KOH+0.5M Zn_3_(PO_4_)_2_ | 3M H_3_PO_4_+  0.5M Zn_3_(PO_4_)_2_ | Zn-MEM  membrane | 0.5 | 79.2 | / | ^[13]^ |
| Pt/C+RuO_2_ | PANa gel + 6M KOH+0.2M Zn(OAc)_2_ | PVA/EGCG/Ag gel | BPM (commercial) | 3 | 72% | / | ^[14]^ |
| Pt/C+IrO_2_ | NaOH | H_3_PO_4_/NaH_2_PO_4_ | Na-SSE | 0.5 | 70% | / | ^[15]^ |
| Pt/C + IrO_2_ | 0.5 M LiOH + 1 m LiNO_3_ | 0.1 M H_3_PO_4_ + 1 M LiH_2_PO_4_ | LTAP | 0.5 | 63.7% | / | ^[16]^ |
| Pt/C+IrO_2_ | 0.5M ZnSO_4_/0.5 MLiTFSI | 0.5M H_2_SO_4_/1M LiTFSI | PILG | 0.5 | 64.5% | / | ^[17]^ |
| NC@Co-HPNC | 6M KOH | 1M H_2_SO_4_ | BPM (commercial) | 1 | 73% | / | ^[18]^ |

**Reference**

[1] F. J. Vidal-Iglesias, J. Solla-Gullón, A. Rodes, E. Herrero, A. Aldaz, *J. Chem. Educ.* **2012**, *89*, 936-939, https://doi.org/10.1021/ed2007179.

[2] C.-N. Chang, H.-B. Cheng, A. C. Chao, *Environ. Sci. Technol.* **2004**, *38*, 1807-1812, https://doi.org/10.1021/es021088e.

[3] J. T. Bender, R. Y. Sanspeur, N. Bueno Ponce, A. E. Valles, A. K. Uvodich, D. J. Milliron, J. R. Kitchin, J. Resasco, *J. Am. Chem. Soc.* **2025**, *147*, 37819-37832, https://doi.org/10.1021/jacs.5c14208.

[4] N. Ramaswamy, S. Mukerjee, *J. Phys. Chem. C* **2011**, *115*, 18015-18026, https://doi.org/10.1021/jp204680p.

[5] D. Xi, A. M. Alfaraidi, J. Gao, T. Cochard, L. C. I. Faria, Z. Yang, T. Y. George, T. Wang, R. G. Gordon, R. Y. Liu, M. J. Aziz, *Nat. Energy* **2024**, *9*, 479-490, https://doi.org/10.1038/s41560-024-01474-1.

[6] B. Tang, Q. Ji, X. Zhang, R. Shi, J. Ma, Z. Zhuang, M. Sun, H. Wang, R. Liu, H. Liu, C. Wang, Z. Guo, L. Lu, P. Jiang, D. Wang, W. Yan, *Angew. Chem. Int. Ed.* **2025**, *64*, e202424135, https://doi.org/10.1002/anie.202424135.

[7] L. Liu, L. Kang, J. Feng, D. G. Hopkinson, C. S. Allen, Y. Tan, H. Gu, I. Mikulska, V. Celorrio, D. Gianolio, T. Wang, L. Zhang, K. Li, J. Zhang, J. Zhu, G. Held, P. Ferrer, D. Grinter, J. Callison, M. Wilding, S. Chen, I. Parkin, G. He, *Nat. Commun.* **2024**, *15*, 4079, https://doi.org/10.1038/s41467-024-48209-0.

[8] C. Lin, S.-H. Kim, Q. Xu, D.-H. Kim, G. Ali, S. S. Shinde, S. Yang, Y. Yang, X. Li, Z. Jiang, J.-H. Lee, *Matter* **2021**, *4*, 1287-1304, https://doi.org/10.1016/j.matt.2021.01.004.

[9] W. Chen, S. Guo, L. Qin, L. Li, X. Cao, J. Zhou, Z. Luo, G. Fang, S. Liang, *Adv. Funct. Mater.* **2022**, *32*, 2112609, https://doi.org/10.1002/adfm.202112609.

[10] J. P. Perdew, K. Burke, M. Ernzerhof, *Phys. Rev. Lett.* **1996**, *77*, 3865-3868, https://doi.org/10.1103/PhysRevLett.77.3865.

[11] Z. Wang, Y. Wang, J. Wang, Y. Song, M. J. Robson, A. Seong, M. Yang, Z. Zhang, A. Belotti, J. Liu, G. Kim, J. Lim, Z. Shao, F. Ciucci, *Nat. Catal.* **2022**, *5*, 777-787, https://doi.org/10.1038/s41929-022-00829-9.

[12] Y. Yin, H. Huang, S. Boulfrad, H. Dai, Y. Gu, S. Yu, L. Bi, *Energy Environ. Sci.* **2025**, *18*, 8130-8141, https://doi.org/10.1039/D5EE00993F.

[13] H. Zhang, M. Zhu, H. Tang, Q. Lu, T. Yang, X. Wang, B. Chen, Z. Qu, X. Wang, M. Yu, D. Karnaushenko, D. D. Karnaushenko, Y. Huang, O. G. Schmidt, K. Zhang, *Energy Storage Mater.* **2023**, *59*, 102791, https://doi.org/10.1016/j.ensm.2023.102791.

[14] Q. Li, Y. Wang, Z. Song, X. Zhu, X. Song, Y. Chen, Y. Zhang, *Angew. Chem. Int. Ed.* **2025**, *137*, e202424318, https://doi.org/10.1002/anie.202424318.

[15] X. Yu, M. M. Gross, S. Wang, A. Manthiram, *Adv. Energy Mater.* **2017**, *7*, 1602454, https://doi.org/10.1002/aenm.201602454.

[16] L. Li, A. Manthiram, *Adv. Energy Mater.* **2016**, *6*, 1502054, https://doi.org/10.1002/aenm.201502054.

[17] Y.-f. Cui, Y.-h. Zhu, J.-y. Du, Y.-l. Zhang, K. Li, W.-q. Liu, G. Huang, X.-b. Zhang, *Joule* **2022**, *6*, 1617-1631, https://doi.org/10.1016/j.joule.2022.05.019.

[18] Z. Chi, Y. Feng, Y. Ma, D. Kong, H. Yin, S. Li, L. Li, Z. Guo, L. Wang, *Chem. Commun.* **2021**, *57*, 11248-11251, https://doi.org/10.1039/D1CC04443E.
